# MDTransport: A Modular, Open-Source, Extensible Python Tool for Computing Transport Properties from GROMACS and LAMMPS Simulations

**DOI:** 10.1021/acs.jcim.6c00558

**Published:** 2026-08-01

**Authors:** Ashutosh Kumar Verma, Amey S. Thorat, Jindal K. Shah

**Affiliations:** School of Chemical Engineering, 7618 Oklahoma State University, Stillwater, Oklahoma 74078, United States

## Abstract

Discovering novel materials for energy-efficient separations, electrochemical reactions, and energy storage requires characterization of thermophysical properties such as diffusivity and ionic conductivity. Over the past several decades, molecular dynamics (MD) simulations have been playing an important role for predictions of properties in a materials-discovery pipeline. In a typical property estimation workflow, trajectories of materials/fluids of interest are generated using MD simulation engines such as GROMACS or LAMMPS, which are then processed to calculate properties such as molar volume, self-diffusion coefficients, ionic conductivity, radial distribution functions, etc. Several postprocessing tools have been developed to facilitate various property calculations. However, many of these tools are geared toward analyzing trajectories produced from a specific MD engine, requiring expertise in multiple postprocessing tools if more than one MD engine is employed, which is very likely given the unique functionalities offered by various MD engines. In addition, these tools are typically limited to routinely computed properties from MD trajectories and do not readily support more advanced analyses, such as cage correlation lifetimes, elucidation of transport mechanisms, or decomposition of ionic conductivity into self- and cross-correlation contributions. To address this research gap, we introduce MDTransport, a comprehensive, open-source, Python-based postprocessing tool, optimized for the estimation of ionic conductivity, self-diffusion coefficient, Onsager transport coefficient, and transference number. It also calculates ion–ion correlation, performs spatial decomposition of cross-correlations, estimates pair and cage correlation lifetimes, performs cluster analysis, and predicts ion-transport mechanisms, thereby providing insights into the structure of the electrolyte and underlying ion dynamics. An intuitive, user-friendly, modular, plug-and-play design, with a prompt-based command-line interface, also allows customization of various input parameters, aiming to serve researchers from diverse research backgrounds and a wide range of programming expertise.

## Graphical abstract

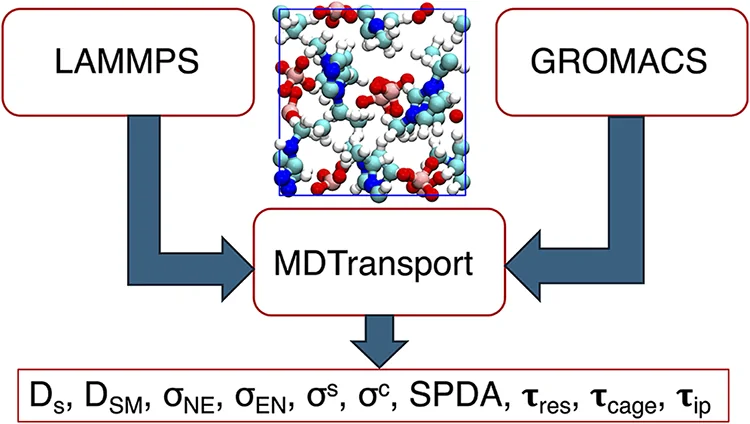

## Introduction

Ionic conductivity in electrolytes is crucial for the efficient operation of energy storage devices such as batteries and supercapacitors, as it governs how quickly and effectively ions can migrate through an electrolyte, directly impacting the overall performance. Molecular dynamics (MD) simulations are extensively used to predict transport properties such as self-diffusion coefficients, ionic conductivity, transference number, etc., for a wide range of electrolytes, including aqueous salts,^1^ ionic liquids,^2^ deep eutectic solvents,^3^ and their mixtures.^4–6^ A variety of molecular simulation software packages such as GROMACS,^7^ LAMMPS,^8^ AMBER,^9^ CHARMM,^10^ NAMD,^11^ DL_POLY,^12^ and OpenMM^13^ generate trajectories of systems under the influence of intramolecular and intermolecular interactions. These trajectories are then processed to extract thermophysical and transport properties of interest. Thus, postprocessing forms an integral part of any computational workflow.

Several postprocessing tools are currently available for extracting useful information from molecular simulation trajectories, including PyLAT,^14^ TRAVIS,^15^ StreaMD,^16^ nMOLDYN,^17^ LiquidLib,^18^ MDAnalysis,^19^ MDTraj,^20^ Chemfiles,^21^ OVITO,^22^ and VMD.^23^ These tools vary significantly with respect to their capabilities, architecture, and implementation, compatibility, robustness to handle large or complex systems, ease of use, and ability to compute different transport properties. While MDTraj and OVITO are powerful tools for parsing MD trajectories, they have limitations regarding topology management. They do not natively extract atomic charges, largely because their primary input formats (e.g., .gro or .pdb) lack complete force-field information. Consequently, these tools often lack direct support for rich topology containers, such as GROMACS .tpr files or LAMMPS data files, which are essential for transport-property analyses like ionic conductivity. In contrast, MDAnalysis utilizes a frame-based iteration approach, which is memory-efficient for large trajectories and provides a data-rich topology, including atomic charges and masses. However, calculating complex transport properties such as Einstein conductivity or ion–ion correlations^24–26^ often remains challenging and requires significant custom scripting, even with these features. Furthermore, packages such as Chemfiles, OVITO, MDTraj, and MDAnalysis demand knowledge of Python programming, presending difficulties for nonexperts. PyLAT, while robust, is constrained by its design to process only LAMMPS trajectories.

As many existing postprocessing tools are designed for specific simulation engines (e.g., GROMACS or LAMMPS), they suffer from overcustomizability, require significant human intervention, or coding experience, and new users can expect a steep learning curve. Additionally, these tools can be difficult to apply for estimating properties when a high-throughput screening is implemented. These limitations suggest that there is room for developing a comprehensive, user-friendly, platform-independent, open-source, scalable postprocessing tool to cater to a diverse user base, which is also suitable for high-throughput screening applications. In this work, we introduce MDTransport, a Python tool that can process trajectories generated with GROMACS and LAMMPS. MDTransport is capable of estimating a wide range of properties including self-diffusion coefficients, Onsager transport coefficients, Einstein and Nernst–Einstein ionic conductivity, and transference numbers.

MDTransport also provides detailed insights into ion–ion correlation, spatial decomposition of cross-correlations, pair and cage correlation lifetimes, cluster analysis, ion-transport mechanism, and radial distribution functions. The implementation of adaptive fitting procedures reduces the uncertainty associated with the manual choice of time windows often required to ensure that the system is, for example, in a diffusive regime. Moreover, MDTransport is highly automated and flexible, capable of handling long simulations and complex systems with minimal manual intervention. These features make it well-suited for high-throughput screening of transport properties across a broad range of materials. To cater to a diverse user base, the MDTransport framework provides three distinct interfaces: a prompt-based command-line interface (CLI) for high-performance computing (HPC) environments, a cross-platform desktop application for local execution, and a cloud-based web interface for seamless, browser-based analysis. This multitiered architecture ensures that advanced trajectory analysis is accessible to users regardless of their programming expertise or computational environment.

## Structure and Implementation

MDTransport is designed to be inherently compatible for use on personal computers as well as high-performance computing (HPC) environments alike, making it accessible to a wide range of users. It implements multiprocessing and can utilize multiple cores to analyze large chunks of data. Furthermore, it employs strategies for efficient memory management, such as snapshot-wise processing of trajectory data and the use of shared memory buffers to minimize data duplication. By utilizing high-performance, machine-readable formats (e.g., GROMACS .xtc or LAMMPS .dcd/.xtc binary files) where supported, MDTransport minimizes memory overhead by loading only the necessary trajectory segments. Moreover, it provides both in-memory and disk-based processing modes for extracting atomic and center-of-mass (COM) data, enabling scalable analysis of large systems by offloading intermediate results to disk when necessary. This can be particularly advantageous in processing long trajectories and/or large systems.

MDTransport uses a modular, class-based approach, where each component of the overall workflow, including transport-property calculation, is defined as a class, allowing seamless exchange of data and parameters internally and externally. It utilizes the *argparse* module to accept input parameters from users through relevant prompts on the CLI. MDTransport contains dedicated Python scripts to handle individual tasks, including input-output operations, validation, and property calculation. It is written using Python, leveraging the capabilities of popular libraries such as pandas, numpy, scipy, matplotlib, and tqdm, making it powerful yet accessible. Extensive documentation is provided to guide users through the workflow. Help can be availed for most functions by using the -h flag, while the full commands for estimating specific properties can be accessed using -e flag.

Figure 1(a) illustrates the file organization of the MDTransport repository. The root directory contains three primary files: the LICENSE.txt file (GPL License), the README.md documentation, and the mdt.py script. The latter serves as a convenient entry point for users to execute the application directly from the source code without requiring a formal installation via PyPI or Conda. The core functionality is implemented within the mdtransport/ subdirectory. The main.py script acts as the central controller, managing the prompt-based interface, parsing user inputs, and validating arguments. The gromacs.py and lammps.py modules contain platform-specific instructions for processing trajectories and extracting the data (atomic/center-of-mass (COM) coordinates, charges, box lengths, etc.) from GROMACS and LAMMPS, respectively. These scripts delegate the numerical computation to the various utility modules, prefixed with utils_, which perform specific property calculations (e.g., utils_msd.py, utils_einstein.py). This modular design ensures that the physical calculation algorithms remain independent of the underlying simulation engines. Finally, utils.py provides shared helper functions (e.g., logger, folder generations) and routines used throughout the package. Example data (GROMACS and LAMMPS) for a 64-ion-pair system of the ionic liquid 1-ethyl-3-methylimidazolium bis­(trifluoromethylsulfonyl)­imide is provided in the sample directory for testing purposes. Larger-scale simulation trajectories and comprehensive validation results are hosted on the project’s GitHub repository.

**Figure 1:**
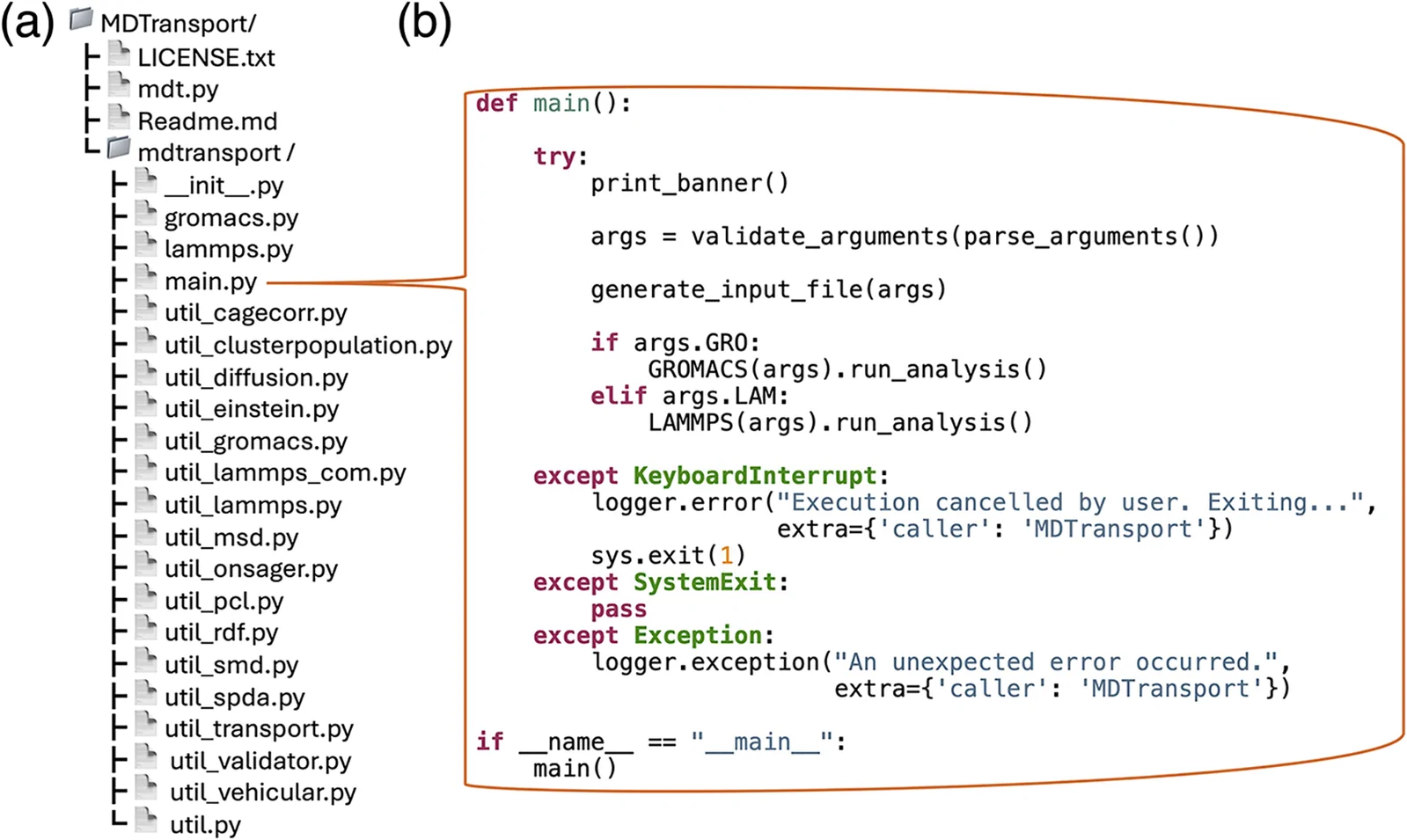
(a) Structure of the MDTransport package, including main script and modules. (b) Main script executing GROMACS and LAMMPS analysis.

## Workflow

The workflow in MDTransport is designed for the optimum use of computational resources, fast results, and ease of use. Intuitive prompts allow the user to select the relevant options and navigate through each step of the workflow stepwise. Expert users may provide all necessary inputs using a single command with appropriate flags for fast implementation. This makes MDTransport accessible to new users and experts alike.

Figure 2 represents the overall workflow implemented in MDTransport. The first step of the workflow is to identify the trajectory file and gather the required topological information. Based on the MD software (GROMACS or LAMMPS), the identity, total number, and net charge on unique chemical species may be extracted from the .*tpr* file for GROMACS or the .*data* file for LAMMPS-based trajectories. Input parameters such as the target transport property, step-size, trajectory length, temperature, etc., are obtained subsequently. If required arguments are missing or invalid, MDTransport interactively prompts the user for correct inputs. Argument validation is implemented in the main script (main.py) and defined in the common validator (validator.py) and utility (util.py) modules (Figure 1(a)). Upon successful validation, MDTransport calls the appropriate analysis module (either gromacs.py or lammps.py depending on the analysis type specified by the user) (Figure 1(b)).

**Figure 2:**
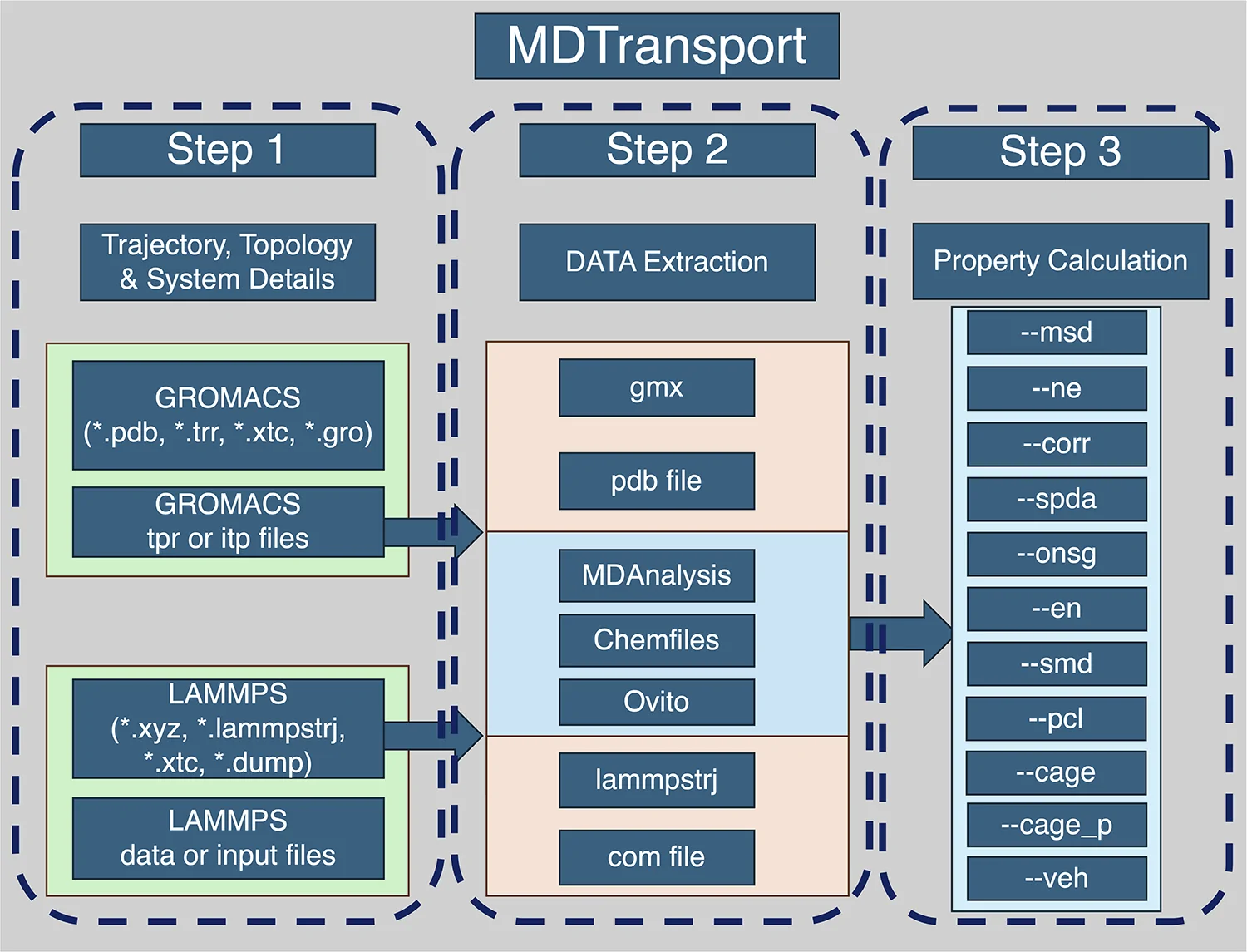
Schematic representation of the MDTransport workflow, illustrating how trajectory data from GROMACS and LAMMPS are processed to extract species center-of-mass, charges, and counts for subsequent transport analyses. Note: gmx uses gmx trjconv to generate pdb files, and then, a custom parser developed in this work is used for data extraction.

After the validation of input parameters, either atomic coordinates are extracted or COM calculations are performed for each molecule over the relevant time of the trajectory dependent on the requested analysis. For example, computing atom–atom RDFs, such as analyzing hydrogen bonding between the imidazolium cation ([C_2_mim]^+^) and the ([BF_4_]^−^) anion (H–F distances) or calculating the mean-squared displacement (MSD) of specific functional groups (e.g., the methyl group of the [C_2_mim]^+^), requires raw atomic coordinates rather than COM representations. On the other hand, for bulk transport properties such as diffusion coefficients, ionic conductivity, and Onsager coefficients, the MDTransport performs COM calculations for each molecule. This selective data extraction ensures computational efficiency while maintaining the physical rigor necessary for each specific property. Moreover, the use of COM coordinates facilitates a several-fold reduction in the size of data, while retaining the essence of system dynamics. This may be achieved in multiple ways such as through explicit calculation of the COMs from a .pdb (providing --pdb flag) or .lammpstrj file (providing --lamp flag) by using the inherent capabilities of GROMACS or LAMMPS or by using tools such as MDAnalysis, Chemfiles, or OVITO, if such tools are installed on the system. Furthermore, extraction of COM can be achieved easily by providing the appropriate flags such as --exe, --mda, --chem, and --ovito. Incorporating these tools in the workflow can offer significant benefits to processing speed and memory requirements due to their capability of processing multiple file formats, including binary files.

Next, simulation cell dimensions are extracted for every snapshot to determine the volume and correctly apply periodic boundary conditions (PBC). During this stage, MDTransport stores simulation box dimensions and COM of species in NumPy arrays, and species charges and their respective counts in a dictionary. Furthermore, a two-step coordinate preprocessing procedure is employed before COM extraction. Initially, molecules fragmented across periodic boundaries are identified and reconstructed using the minimum image convention, ensuring that each molecule is treated as a physically contiguous entity. Subsequently, to maintain trajectory continuity, the tool monitors molecular displacements between consecutive frames. If a displacement exceeds half the simulation box length along any dimension, a correction is applied to eliminate artificial jumps resulting from boundary crossings while reconstructing broken molecules as whole molecules. Further, MDtransport identifies and groups chemical species based on their residue names (resnames). For GROMACS analysis, this information is automatically extracted from files that natively store residue metadata, such as .pdb, .gro, .tpr, or .itp formats. In contrast, LAMMPS data files and trajectories typically identify species using only numeric atom or molecule IDs. Consequently, MDTransport assigns default labels such as species_1 and species_2, which users can override with descriptive names by using the --species flag (e.g., --species EMI BF).

Once the COMs, box dimensions, and volumes are extracted, MDTransport performs essential preprocessing operations to ensure the physical validity of the transport-property calculations. Specifically, drift removal is implemented by calculating the COM of the entire simulation box at each time step and subtracting it from individual molecular coordinates, effectively removing any net translational motion (system-wide drift) from the trajectory. Further, MDTransport triggers a sequence of functions that calculate the specific properties of interest. Each property is implemented in a separate Python file located at MDTransport/util_property.py file (Figure 1(a)), allowing for modularity. These property-specific functions are called through the shared interfaces provided in gromacs.py and lammps.py, enabling developers to incorporate new properties with minimal modification to the existing code structure by creating a new class for that property.

While the calculations run in the background, the user is informed about the progress via on-screen messages and status bars. Appropriate errors and warning messages are displayed if the process is interrupted due to incorrect or missing information. Technical details such as data processing rate and elapsed time are included to assess the computational performance during the calculation. Detailed output is displayed on the screen, and a report is generated to an external text log, containing the summary of user inputs, processing snapshots, system information such as density, type of calculation, status and duration of intermediate steps, total time, and overall performance. Further, outputs, including raw data and final results, are saved as CSV files within dedicated folders. For example, when an Einstein analysis is requested, MDTransport generates a results_einstein folder to store the collective MSD data and final ionic conductivity results. If a folder with the same name already exists in the current working directory, the tool automatically renames the existing directory (e.g., to #results_einstein#) to preserve the old data, logs this action, and then proceeds to generate a new results_einstein folder for the current results, thereby preventing the inadvertent overwriting of data. Moreover, an input file is generated that includes all available flags, where unused flags are left commented out for reference. The user can rerun the same analysis or use the input file as a template for another analysis by providing the input file using --in input.txt.

## Capabilities: Theory and Implementation

This section expands on the capabilities of MDTransport, providing theoretical background and implementation details for each property. Command usage and example outputs are provided in the Supporting Information.

### Mean-Squared Displacement (MSD) and Self-Diffusion Coefficient (D)

MDTransport employs the Einstein formalism to compute the self-diffusion coefficients of species by first computing the mean-squared displacement of the species of interest (atom, group of atoms, or molecules) as a function of the time interval Δ*t*

1 $${\mathrm{MSD}}_{k}=\frac{1}{N_{k}}\sum_{i=1}^{N_{k}}\left\langle {\left[r_{i}^{c}\left(t+\Delta t\right)-r_{i}^{c}\left(t\right)\right]}^{2}\right\rangle$$

The self-diffusion coefficient is calculated by computing the slope of the MSD versus time.^27^

2 $$D_{k}=\lim_{\Delta t\to \infty }\frac{1}{2d\Delta t}\left(\frac{1}{N_{k}}\left\langle \sum_{i=1}^{N_{k}}{\left[r_{i}^{c}\left(t+\Delta t\right)-r_{i}^{c}\left(t\right)\right]}^{2}\right\rangle \right)$$

Here, *N*_*k*_ is the number of the *k* ^th^ group, which can be a specific atom, or the COM for a group of atoms or molecules. *r*_*i*_^c^ is the location of the COM of the molecule or the position of a given atom of interest. The ⟨···⟩ represents time averaging, and *d* is the dimensionality of the system. By default, MSD and self-diffusion coefficients are averaged over the *x*, *y*, and *z* directions (*d* = 3); however, it is possible to compute MSD only along one (*d* = 1) or two directions (*d* = 2) by specifying the direction(s) along which MSD is to be evaluated, e.g., --dir *x* or --dir *x* *y* . This functionality could be useful for analyzing systems with structural constraints, such as transport within nanotubes or diffusion parallel/perpendicular to an interface in slab geometries, where motion is either restricted to a specific direction or anisotropic. To calculate the self-diffusion coefficient (D), MDTransport tracks the trajectories of individual entities over time. By invoking the --msd flag, it calculates the COM-based MSD for each individual molecule within a species (identified via residue names, resnames) and averages them to yield the self-diffusion coefficient. Users can restrict this calculation to a specific species using the --resname flag. Furthermore, users can calculate the self-diffusion coefficient of specific intramolecular fragments (e.g., tracking the COM of just the head or tail group of an individual imidazolium cation) rather than the whole molecule. This is achieved by combining the --msd and --msd_mode flags and defining the specific intramolecular atomic fragment using the --msd_species flag. In all of these cases, the MSD is computed for each individual entity separately before averaging across the system, ensuring the result strictly represents self-diffusion coefficient rather than a collective diffusion coefficient. The right-hand side of MSD in eq 1 can be expanded as

3 $${\left[r_{i}^{c}\left(t+\Delta t\right)-r_{i}^{c}\left(t\right)\right]}^{2}={\left[r_{i}^{c}\left(t+\Delta t\right)\right]}^{2}+{\left[r_{i}^{c}\left(t\right)\right]}^{2}-2\left[r_{i}^{c}\left(t+\Delta t\right)\cdot r_{i}^{c}\left(t\right)\right]$$

eq 3 contains three terms, of which the first two terms are the sum of the squares of the magnitude of the position vectors evaluated for all possible time steps that are separated by Δ*t*. The third term represents twice the time-autocorrelation of the position vector *r*_*i*_^*c*^ at Δ*t*. In MDTransport, it is computed using fast Fourier transform (FFT), as employed in the work by Fong et al.^28^ Thus, the final equation for calculating MSD can be written as

4 $${\mathrm{MSD}}_{k}=\frac{1}{N_{k}}\sum_{i=1}^{N_{k}}\left\langle {\left[r_{i}^{c}\left(t+\Delta t\right)\right]}^{2}\right\rangle +\left\langle {\left[r_{i}^{c}\left(t\right)\right]}^{2}\right\rangle -2\left\langle \left[r_{i}^{c}\left(t+\Delta t\right)\cdot r_{i}^{c}\left(t\right)\right]\right\rangle$$

### Calculation of Self-Diffusion Coefficient

In general, a variety of protocols have been proposed in the literature to estimate uncertainties in diffusion coefficients obtained from MD simulations, including block averaging, time-window variation, and ensemble-based approaches.^27,29–31^ For the calculation of self-diffusion coefficients, MDTransport evaluates the diffusion regime over a user-defined time interval, as determined from the slope of ln MSD versus ln *t* approaching unity (eq 5).^6,32,33^

5 $$\beta_{i}=\frac{\partial \;\ln{\mathrm{MSD}}_{i}}{\partial \;\ln t}$$

By default, the full MSD data is considered, while users may optionally specify a fitting window (--msd_fit) to restrict the analysis to a desired time range. The self-diffusion coefficient is then evaluated over the diffusive regime. This approach mitigates errors introduced by including nondiffusive MSD data, such as ballistic motion at short time scales or finite-size effects at long time scales. In PyLAT^14^ or a recent work by Frömbgen et al.,^34^ the diffusive region is identified by a slicing method to calculate MSD until β_*i*_ is within 1 ± δ, where δ is the user-defined tolerance. We adopt a different approach, which relies on analyzing MSD data over a time window of at least 8 ns for trajectories longer than 10 ns or half the trajectory length for shorter trajectories. Users may also provide their own minimum lengths of time window using --min_window flag. It identifies a region where the cumulative deviation of β_*i*_ from 1 is negligible. To achieve this, β_*i*_ values are calculated for all possible time windows (with an increment of 0.1 ns), and the best time window is selected based on the condition (∑_*i*_^*N*^|β_*i*_ – 1|)_*min*_, where *N* represents the number of species present in the system. This approach ensures the selection of a time window where all of the species are in the diffusive regime, leading to accurate and reliable diffusion coefficient calculations. If ${\left(\frac{1}{N}\sum_{i}^{N}\left|\beta_{i}-1\right|\right)}_{\min}$ is greater than a tolerance value (δ = 0.075),^14^ MDTransport will compute self-diffusion coefficients but display a warning as the average deviation is below the expected threshold.

MDTransport provides three fitting methodologies to estimate self-diffusion coefficients: ordinary least squares (OLS), weighted least squares (WLS), and generalized least squares (GLS). By default, it employs OLS, performing a standard linear regression on the MSD data within the identified diffusive regime. For more advanced statistical analysis, MDTransport implements the binary-based sampling and fitting procedure proposed by Moustafa et al.^35^ and Bullerjahn et al.^36^ When the --fit_method flag is set to wls, MDTransport performs a weighted least squares fit, utilizing the number of independent samples at each interval to weight the residuals as proposed by Moustafa et al.^35^ and Bullerjahn et al.^36^ When set to gls, MDTransport employs the full generalized least squares estimator as given by Moustafa et al.^35^ The syntax for MSD-related analysis is included in S2.1 of Supporting Information.

### Ionic Conductivity Using Nernst–Einstein Formalism

MDTransport provides two options for calculating ionic conductivity via Nernst–Einstein and Einstein formalism. The Nernst–Einstein approach ignores ion–ion correlation and is relevant for solutions containing a dilute concentration of ions. It uses self-diffusion coefficients to estimate ionic conductivity,^24,37,38^ given by

6 $$\sigma_{\mathrm{NE}}=\frac{e^{2}}{Vk_{B}T}\left(N_{1}z_{1}^{2}D_{1}+N_{2}z_{2}^{2}D_{2}+\cdot \cdot \cdot +N_{n}z_{n}^{2}D_{n}\right)=\frac{e^{2}}{Vk_{B}T}\sum_{i=1}^{n}N_{i}z_{i}^{2}D_{i}$$

where *n* is the total number of species, *e*, *k*_*B*_, *V*, and *T* represent the elementary charge, Boltzmann constant, system volume, and temperature, respectively. *z*_*i*_ is the partial charge, and *D*_*i*_ is the self-diffusion coefficient of the *i* ^th^ species. Calculation of the Nernst–Einstein conductivity is straightforward, as it requires the determination of the self-diffusion coefficients of all of the species, which can be accomplished as described above. Users can use the --ne flag to calculate Nernst–Einstein ionic conductivity. The syntax for calculating the Nernst-Einstein ionic conductivity is provided in S2.2 of Supporting Information.

### Ionic Conductivity and Ion–Ion Correlation Using Einstein Formalism

In systems with significant ion–ion correlation, such as pure and mixtures of ionic liquids, and solutions containing high ion concentrations, the Einstein approach is better suited to accurately estimate the ionic conductivity. The expression used for estimating Einstein conductivity is given by^24,38–40^

$$\sigma_{\mathrm{EN}}=\lim_{\Delta t\to \infty }\frac{e^{2}}{2\mathrm{dV}\Delta tk_{B}T}\sum_{i,j}z_{i}z_{j}\left\langle \left[r_{i}^{c}\left(t+\Delta t\right)-r_{i}^{c}\left(t\right)\right]\cdot \left[r_{j}^{c}\left(t+\Delta t\right)-r_{j}^{c}\left(t\right)\right]\right\rangle$$ 7

where the variables have the usual meaning as outlined above. The Einstein conductivity (eq 7) can further be resolved into contributions from self- (*i* = *j*), and cross-correlations (*i* ≠ *j*) to generate insights into dominant ion–ion correlations affecting overall ionic conductivity. The cross-correlations may be further distinguished into intercomponent and intracomponent correlations based on whether the correlation is between ions of the same species or different species, respectively. For example, in a system containing a salt [C]­[A] where [C] represents the cation and [A] the anion, the Einstein conductivity may be expressed as follows^26,28,41–43^

8 $$\sigma_{\mathrm{EN}}=\sigma_{\left[C\right]}^{\mathrm{self}}+\sigma_{\left[A\right]}^{\mathrm{self}}+\sigma_{\left[C\right]-\left[C\right]}^{\mathrm{cross}}+\sigma_{\left[A\right]-\left[A\right]}^{\mathrm{cross}}+\sigma_{\left[C\right]-\left[A\right]}^{\mathrm{cross}}$$

where σ_[C]_^self^ and σ_[A]_^self^ stand for the diffusive contributions due to the cation and anion, respectively, the sum of which is the Nernst–Einstein conductivity (eq 6). σ_[C]‑[C]_^cross^, σ_[A]‑[A]_^cross^, and σ_[C]‑[A]_^cross^ refer to the cross-correlation contributions. MDTransport estimates these contributions by selecting the appropriate values for *i* and *j* in eq 7 for the ions of the respective species. To improve the estimate of the average contained in the summation in eq 7, various time origins are employed; the correlation term is plotted against the time, and the slope obtained over the first 1 ns is used to estimate the Einstein conductivity. This procedure has been adopted from our work^24^ and those of others.^38,39^ The slope calculation over the first 1 ns is based on our experience that the correlation term remains linear over this period for ionic liquids^24,44^ and salt solutions with solvents.^45^ To allow the flexibility to vary the time interval over which the slope is calculated, the user can specify the time window by supplying --min_window flag. The --corr flag enables both ion–ion correlation calculations (self- and cross-correlations) and Einstein ionic conductivity calculations in a single run. Syntax for computing the Einstein conductivity and ion-ion correlations can be found in S2.3 of Supporting Information.

### Ionic Conductivity and Ion–Ion Correlation Using Einstein-Helfand Formalism

MDTransport also computes the Einstein conductivity using the dipole moment of the simulation box, following the Einstein–Helfand (EH) formalism, the derivation for which can be found in previous work.^24,40^

9 $$\sigma_{\mathrm{EN}}=\lim_{\Delta t\to \infty }\frac{\left\langle {\left|M\left(t+\Delta t\right)-M\left(t\right)\right|}^{2}\right\rangle }{2\mathrm{dV}\Delta tk_{B}T}$$

Here,

10 $$M\left(t\right)=\sum_{i=1}^{N_{\mathrm{ions}}}q_{i}\cdot r_{i}\left(t\right)$$

MDTransport uses an expanded form of the Einstein-Helfand equation to allow vectorization from *O*(*N* ^2^) to *O*(*N* log *N*) and uses FFT for autocorrelation terms, similar to computing MSD (eq 3).

11 $$\left|M\left(t+\Delta t\right)-M\left(t\right){|}^{2}=\right|M\left(t+\Delta t\right){|}^{2}+|M\left(t\right){|}^{2}-2M\left(t+\Delta t\right)\cdot M\left(t\right)$$

The EH formalism may be more suitable than other methods, such as the Green–Kubo method, for systems with a large number of ions, as the Green–Kubo method requires the velocity of each ion. On the other hand, the Einstein-Helfand method requires the dipole moment of the system, which significantly reduces computational requirements for analysis. Users can use the --en flag to calculate Einstein ionic conductivity. Note that MDTransport computes ionic conductivity strictly based on fixed-charge (point-charge) models.

Further, the EH formalism can also be utilized to compute ion–ion correlations by considering the collective dipole displacements of the species. The net dipole displacement for all ions of a specific species is defined as

$$\Delta M_{\left[C\right]}=\underset{i\in \left[C\right]}{\sum }q_{i}\left[r_{i}^{c}\left(t+\Delta_{t}\right)-r_{i}^{c}\left(t\right)\right],\Delta M_{\left[A\right]}=\underset{j\in \left[A\right]}{\sum }q_{j}\left[r_{j}^{c}\left(t+\Delta t\right)-r_{j}^{c}\left(t\right)\right]$$ 12

The self-conductivity contribution, representing the uncorrelated motion of individual ions, is given by

$$\sigma_{\left[C\right]}^{\mathrm{self}}=\underset{\Delta t\to \infty }{\lim}\frac{e^{2}}{2\mathrm{dV}\Delta tk_{B}T}\underset{i\in \left[C\right]}{\sum }\left\langle {\left[q_{i}\left(r_{i}^{c}\left(t+\Delta t\right)-r_{i}^{c}\left(t\right)\right)\right]}^{2}\right\rangle$$ 13

The intraspecies cross-correlation terms (e.g., cation–cation) represent the distinct part of the correlation between different ions of the same species

$$\sigma_{\left[C\right]-\left[C\right]}^{\mathrm{cross}}=\underset{\Delta t\to \infty }{\lim}\frac{e^{2}}{2\mathrm{dV}\Delta tk_{B}T}\left[\left\langle {\left(\Delta M_{\left[C\right]}\right)}^{2}\right\rangle -\underset{i\in \left[C\right]}{\sum }\left\langle {\left[q_{i}\left(r_{i}^{c}\left(t+\Delta t\right)-r_{i}^{c}\left(t\right)\right)\right]}^{2}\right\rangle \right]$$ 14

The interspecies cross-correlation terms (cation–anion) account for the interaction between the two different species

15 $$\sigma_{\left[C\right]-\left[A\right]}^{\mathrm{cross}}=\lim_{\Delta t\to \infty }\frac{e^{2}}{\mathrm{dV}\Delta tk_{B}T}\left\langle \Delta M_{\left[C\right]}\cdot \Delta M_{\left[A\right]}\right\rangle$$

where the variables have the usual meaning as outlined above. The total ionic conductivity is obtained by summing these contributions as shown in eq 8. While both methods are mathematically equivalent and yield identical results for any given time window, they differ significantly in their computational scaling. The standard Einstein formalism calculates cross-correlations by explicitly evaluating pairwise displacements between ions. Because this pairwise summation is computationally expensive, the sliding time window is typically restricted to a shorter, user-defined range (--n_delta) to maintain practical computation times while still capturing the linear diffusive regime. In contrast, the EH formalism calculates the correlation using the dipole displacement of the entire species as a single variable, completely bypassing the expensive pairwise summations. Due to this massive reduction in computational overhead, the EH method evaluates the correlations much faster. This efficiency gives users the practical ability to compute correlation over much longer sliding windows up to the entire trajectory length without facing high computational costs. This accelerated approach can be enabled in MDTransport by providing the --eh flag alongside the --corr option. Syntax for calculating the ionic conductivity using this approach is given in S2.4 of Supporting Information.

### Spatial Decomposition Analysis of Ion–Ion Correlations (SPDA)

Movement of ions is strongly influenced by the electrostatic and LJ interactions with its immediate neighbors. However, due to the strong long-range effects of these forces, the movement of ions may also be influenced by ions located beyond the first and second solvation shells. Understanding how ions in various solvation shells are responsible for ion–ion correlations (eq 8) can provide molecular-level insight that can be leveraged to tune ionic conductivity of electrolytes through a rational choice of the cations, anions, and solvents. These effects can be uncovered by estimating the variation in the magnitude of the ion–ion correlations as a function of distance from the reference ion. In MDTransport, this is achieved by categorizing ions into two subgroups: ions found within a specified distance *r*_cut_ with respect to the reference ion and those that occupy positions beyond *r*_cut_. Ionic conductivity arising from the correlations within the ions in *r*_cut_ is termed as σ_proximal_ (eq 16) while the contributions due to the ions beyond *r*_cut_ is designated as σ_distant_ (eq 17). This yields three subsets of *j* ions around every *i* ^*th*^ ion (ions stay within the cutoff radius, ions stay outside the cutoff radius, and ions jumping around the cutoff radius). These are then used to estimate eq 7 around the ion.^26,28,41–43^

$$\sigma_{\mathrm{proximal}}^{c}=\underset{\Delta t\to \infty }{\lim}\frac{e^{2}}{2\mathrm{dV}\Delta tk_{B}T}\underset{\begin{array}{l} i\neq j\\ r_{\mathrm{ij}}<r_{\mathrm{cut}} \end{array}}{\sum }z_{i}z_{j}\left\langle \Delta r_{i}^{c}\left(\Delta t\right)\cdot \Delta r_{j}^{c}\left(\Delta t\right)\right\rangle$$ 16

$$\sigma_{\mathrm{distant}}^{c}=\underset{\Delta t\to \infty }{\lim}\frac{e^{2}}{2\mathrm{dV}\Delta tk_{B}T}\underset{\begin{array}{l} i\neq j\\ r_{\mathrm{ij}}\geq r_{\mathrm{cut}} \end{array}}{\sum }z_{i}z_{j}\left\langle \Delta r_{i}^{c}\left(\Delta t\right)\cdot \Delta r_{j}^{c}\left(\Delta t\right)\right\rangle$$ 17

MDTransport defines the first and second solvation shells as the intermolecular distances corresponding to the first and second minima of the RDF, respectively. To streamline the analysis workflow, MDTransport automatically computes the required RDF, identifies the position of the second minimum, and dynamically sets it as the default r_*cut*_ for the spatial decomposition analysis. Users can manually override this behavior and specify the first solvation shell via --shell flag or any other arbitrary distance using the --rcut flag. The slopes of the collective MSD, defined in eqs 16 and 17, are calculated over a 1 ns time window (user may change it using --t_window flag). In MDTransport, the spatial decomposition analysis may be performed in two ways depending on how the locality of ions is defined. In the fixed approach,^26^ identities of ions in each of the subgroups are obtained based on their initial positions and maintained throughout the analysis, irrespective of the relative distances at a later time step. In the variable approach, subgroups are updated at every time step. The sum of the contributions from proximal and distant subgroups equals the total cross-correlation contributions in the fixed approach. On the other hand, additional cross-correlations emerge due to the exchange of particles across the solvation boundary during the course of a simulation. By default, MDTransport outputs SPDA based on fixed and variable approaches when --spda flag is supplied. The reader is referred to S2.5 of Supporting Information for syntax to conduct spatial decomposition analysis of ion-ion correlations.

### Onsager Transport Coefficients

MDTransport estimates the Onsager transport coefficients for species using COM and employs an approach analogous to the Einstein formalism, such as one given by various studies.^28,46,47^ It may be expressed using the equation

18 $$L_{\mathrm{ij}}=\lim_{\Delta t\to \infty }\frac{1}{2\mathrm{dV}\Delta tk_{B}T}\sum_{i,j}\left\langle \left[r_{i}^{c}\left(t+\Delta t\right)-r_{i}^{c}\left(t\right)\right]\cdot \left[r_{j}^{c}\left(t+\Delta t\right)-r_{j}^{c}\left(t\right)\right]\right\rangle$$

Equation 18 can be further decomposed into self- and cross-terms by choosing the appropriate values for *i* and *j*

19 $$L_{\mathrm{ii}}^{\mathrm{self}}=\lim_{\Delta t\to \infty }\frac{1}{2\mathrm{dV}\Delta tk_{B}T}\sum_{i}\left\langle [r_{i}^{c}\left(t+\Delta t\right)-r_{i}^{c}\left(t\right){]}^{2}\right\rangle$$

20 $$L_{\mathrm{ij}}^{\mathrm{cross}}=\lim_{\Delta t\to \infty }\frac{1}{\mathrm{dV}\Delta tk_{B}T}\sum_{i>j}\left\langle \left[r_{i}^{c}\left(t+\Delta t\right)-r_{i}^{c}\left(t\right)\right]\cdot \left[r_{j}^{c}\left(t+\Delta t\right)-r_{j}^{c}\left(t\right)\right]\right\rangle$$

The ionic conductivity can be obtained using Onsager transport coefficients as given below

21 $$\kappa ={\left(N_{A}e\right)}^{2}\sum_{i,j}z_{i}z_{j}L_{\mathrm{ij}}$$

Here, *N*_*A*_ is the Avogadro constant. Moreover, Onsager transport coefficients are dependent on the chosen reference frame. While standard molecular dynamics simulations typically extract these coefficients in the global COM or simulation box frame, formulating experimental macroscopic transport equations requires coefficients defined relative to a specific reference species, such as a solvent.^48,49^ To account for this, MDTransport calculates Onsager coefficients beyond the global COM frame. Using the --ref_species flag, molecular displacements are transformed relative to the mass-weighted center-of-mass displacement of the targeted reference species

22 $$\Delta r_{i}^{\mathrm{rel}}=\Delta r_{i}-\frac{\sum_{k\in \mathrm{ref}}m_{k}\Delta r_{k}}{\sum_{k\in \mathrm{ref}}m_{k}}$$

where Δ*r* is *r* ^*c*^(*t* + Δ*t*) – *r* ^*c*^(*t*), *m*_*k*_ and Δ*r*_*k*_ are the mass and displacement of the *k* ^th^ molecules belonging to the reference species, respectively. To reduce computational cost, one can select a time window for time origin shifting (sliding window) by setting --n_delta to 1000 or 2000 ps (default: 1000 ps) and n_delta ≥ t_window, where t_window is the initial time window for slope calculations. MDTransport also computes Onsager transport coefficients using EH formalism and uses the similar method as discussed in the Ion–Ion Correlation Section. Similar to the calculation of ion–ion correlations, both the standard Einstein and EH methods yield identical results for Onsager transport coefficients within any given time window. However, the standard approach requires the explicit calculation of pairwise cross-displacements between all molecules, which is computationally demanding. Consequently, the sliding time window is practically restricted to the user-defined --n_delta parameter to maintain reasonable computation times. In contrast, the EH approach computes these coefficients by first summing the COM displacements of all particles within a given species (without charge weighting) to create a single collective displacement variable. By correlating these collective variables rather than individual pairs, the computational overhead is drastically reduced. This efficiency provides users with the practical flexibility to evaluate transport coefficients over much longer sliding windows up to the entire trajectory length without facing prohibitive computational costs. This accelerated algorithm is enabled by providing the --eh flag in conjunction with the --onsg option. The command line options for computing Onsager transport coefficients are given in S2.6 of Supporting Information.

### Transference Numbers

The transference number provides details on the contribution from each ionic species toward the overall charge transport. Like ionic conductivity, transference numbers may be estimated using the Nernst–Einstein and Einstein formalisms, yielding ideal transference number and transference number taking into account ion–ion correlations, respectively.^46,50,51^ The ideal transference number is estimated using the following equation

23 $$t_{i}^{\mathrm{ideal}}=\frac{N_{i}z_{i}^{2}D_{i}}{\sum N_{i}z_{i}^{2}D_{i}}$$

where *N*_*i*_ is the number of charge carriers of species, *z*_*i*_ is the partial charge, and *D*_*i*_ is the self-diffusion coefficient of the *i* ^th^ species. The real transference number is given by

24 $$t_{i}^{\mathrm{real}}=\frac{\sigma_{i}^{\mathrm{self}}+\sigma_{i-i}^{\mathrm{cross}}+0.5\sum_{i\neq j}\sigma_{i-j}^{\mathrm{cross}}}{\sigma_{\mathrm{EN}}}$$

where, σ_i_^self^ and σ_i–i_^cross^ represent the contributions due to self-diffusion and intracomponent interaction in the *i* ^th^ species, while σ_i–j_^cross^ represents contributions from the intercomponent correlations between the *i* ^th^ and *j* ^th^ species, respectively. Transference numbers are computed along with the Einstein ionic conductivity. Please refer to S2.4 of Supporting Information.

### Stefan-Maxwell Diffusivity

Stefan-Maxwell (SM) diffusion coefficient is a key quantity in multicomponent systems. MDTransport computes SM-MSD for the pair of species (*i*, *j*) as^52,53^

$${\mathrm{MSD}}_{\mathrm{ij}}=\left\langle \frac{1}{N_{i}N_{j}}\overset{N_{i}}{\underset{i=1}{\sum }}\overset{N_{j}}{\underset{j=1}{\sum }}{\left|x_{j}\Delta {\mathrm{r⃗}}_{i}^{c}\left(\Delta t\right)-x_{i}\Delta {\mathrm{r⃗}}_{j}^{c}\left(\Delta t\right)\right|}^{2}\right\rangle$$ 25

where ⟨···⟩ is an average over all possible time origins, Δ*r⃗*_*i*_ and Δ*r⃗*_*j*_ represent the displacements over time interval Δ*t*, *x*_*i*_ and *x*_*j*_ denote the mole fractions, and *N*_*i*_ and *N*_*j*_ denote the number of molecules of *i* ^*th*^ and *j* ^*th*^ species, respectively. Expanding eq 25 as

$${\mathrm{MSD}}_{\mathrm{ij}}=x_{j}^{2}\left\langle \frac{1}{N_{i}}\overset{N_{i}}{\underset{i=1}{\sum }}|\Delta {\mathrm{r⃗}}_{i}^{c}\left(\Delta t\right){|}^{2}\right\rangle +x_{i}^{2}\left\langle \frac{1}{N_{j}}\overset{N_{j}}{\underset{j=1}{\sum }}|\Delta {\mathrm{r⃗}}_{i}^{c}\left(\Delta t\right){|}^{2}\right\rangle -\frac{2x_{i}x_{j}}{N_{i}N_{j}}\left\langle \left(\overset{N_{i}}{\underset{i=1}{\sum }}\Delta {\mathrm{r⃗}}_{i}^{c}\left(\Delta t\right)\right)\cdot \left(\overset{N_{j}}{\underset{j=1}{\sum }}\Delta {\mathrm{r⃗}}_{j}^{c}\left(\Delta t\right)\right)\right\rangle$$ 26

The Stefan-Maxwell diffusivity is obtained as

27 $$D_{\mathrm{ij}}=\lim_{\Delta t\to \infty }\frac{{\mathrm{MSD}}_{\mathrm{ij}}}{2d\Delta t}$$

*d* signifies the number of dimensions over which the Stefan-Maxwell diffusivity is calculated. To calculate ionic conductivity from the SM diffusivities, MDTransport constructs and numerically solves a generalized, multicomponent resistance matrix. The complete mathematical derivation of this framework, which relates macroscopic ionic fluxes to applied electrochemical potential gradients, has been rigorously established and is detailed elsewhere.^52,53^ Users can use the --smd flag (S2.7 of Supporting Information) to calculate SM diffusivity and ionic conductivity using D_ij_.

### Cluster Analysis/Cage Population

The dynamics of a species within the first solvation shell around it can provide valuable insights concerning the mobility and correlation of the molecules in its neighborhood. This tends to vary significantly based on the choice of solvent and concentration.^4^ The statistical distribution of the cluster population can reveal trends in agglomeration and degree of solvation. For example, in an electrolyte containing ionic species and a molecular solvent, a large population of ionic species within the first solvation shell can indicate agglomeration, while a solvation shell predominantly populated with solvent molecules around an ionic reference is indicative of a well-solvated system. MDTransport estimates the cluster population by counting the number of molecular or ionic species of choice located within a spherical region of radius *r*_cut_ around a reference species (Figure S12­(c)). To perform this analysis, users provide the --cage_p flag alongside the --resname flag (S2.8 of Supporting Information). If the cutoff radius is not explicitly specified via --rcut, the tool automatically sets *r*_cut_ to the distance of the first minimum in the RDF of the first two species listed in the --resname sequence. In such a case, the cage population is equal to the coordination number. In addition to the average population, the fluctuation in the mean, in the form of the standard deviation, is also produced, which can be related to the ionic conductivity of ionic systems and solutions.^4^

### Cage Correlation Lifetimes

The cluster population analysis can be extended further by tracking the identity of specific species present within the first solvation shell around a reference species. The dynamics of these identities, such as how fast they exchange with the bulk, has provided unique insight into the self-diffusion coefficients of ionic liquids^54^ and ionic conductivity maximum in ionic solutions.^4^ MDTrasnport supports quantification of the dynamics through intermittent cage correlation lifetimes. For this calculation, a cage around a central species *i* is defined using the identities of all molecules or ions of the target species *j* located within the first solvation shell of radius *r*_cut_ inferred from the RDF between *i* and *j*. Persistence of a cage is tracked over time by monitoring the identity of molecules in the cage via Heaviside function, *h*(*t*). At the start *t* = 0, identities of molecules of species *j* are obtained. The Heaviside function is assigned a value of unity for any subsequent time for which the identity of the cage is maintained. If an ion or molecule enters or exits the cage, *h*(*t*) assumes a value of zero. Note that a cage that is broken at time *t* is allowed to reform at a later time. The cage correlation function is obtained by averaging *h*(*t*) over all particles of the reference species at each time step.

28 $$C_{\mathrm{cage}}\left(t\right)=\frac{\left\langle h\left(0\right)\cdot h\left(t\right)\right\rangle }{\left\langle h\left(0\right)\right\rangle }=\frac{N\left(t\right)}{N\left(t_{0}\right)}$$

where *N*(*t*_0_) and *N*(*t*) are the number of cages at time *t* = 0 and *t*, respectively. The cage correlation lifetime τ_*cage*_ is then determined by direct integration of the correlation function over time

29 $$\tau_{\mathrm{cage}}=\int_{0}^{\infty }C_{\mathrm{cage}}\left(t\right)\mathrm{dt}$$

This may be achieved numerically or by fitting the correlation function to an exponential function

30 $$C_{\mathrm{cage}}=a_{1}\exp\left(-t/b_{1}\right)+a_{2}\exp\left(-t/b_{2}\right)+a_{3}\exp\left(-t/b_{3}\right)$$

where *a*_3_ = 1 – (*a*_1_ + *a*_2_) and τ_*cage*_ is obtained as

31 $$\tau_{\mathrm{cage}}=a_{1}b_{1}+a_{2}b_{2}+a_{3}b_{3}$$

To perform a cage correlation lifetime analysis, users must provide the --cage flag alongside the --ref_species and --cage_species flags. If a cutoff radius is not explicitly defined by the user via the --rcut flag, MDTransport automatically determines this parameter. By default, it computes *r*_cut_ by evaluating the RDF between the first species listed in the --ref_species and --cage_species flags. S2.9 of Supporting Information contains the syntax for calculating cage correlation lifetimes.

### Pair Correlation Lifetimes

Similar to the dynamics of a cage, dynamics associated with a pair of molecules can also be obtained in MDTransport. For every reference molecule *i*, a pairing molecule *j* of the target species is identified such that the COM distance between *i* and *j* is the minimum. The dynamics of this pair is tracked over time, and the average time over which this pairing is maintained is the pair correlation time. A Heaviside step function *h*(*t*) is assigned a value of 1 as long as the identity of the nearest target species is maintained. If any other ion or molecule moves closer, *h*(*t*) is changed to 0. Note that a pair that is broken at time *t* is allowed to reform at a later time. The pair correlation function is obtained by averaging *h*(*t*) over all molecules or ions of the reference species at each time step.^54^

32 $$C_{\mathrm{pair}}\left(t\right)=\frac{\left\langle h\left(0\right)\cdot h\left(t\right)\right\rangle }{\left\langle h\left(0\right)\right\rangle }=\frac{N\left(t\right)}{N\left(t_{0}\right)}$$

where *N*(*t*_0_) and *N*(*t*) are the number of pairs at time *t* = 0 and *t*, respectively. The pair correlation lifetime τ_pair_ is then determined as outlined for the cage correlation lifetime.

$$\tau_{\mathrm{pair}}=\int_{0}^{\infty }C_{\mathrm{pair}}\left(t\right)\mathrm{dt}$$ 33

This may be achieved numerically or by fitting the correlation function to an exponential function

34 $$C_{\mathrm{pair}}=a_{1}\;\exp\left(-t/b1\right)+a_{2}\;\exp\left(-t/b_{2}\right)+a_{3}\;\exp\left(-t/b_{3}\right)$$

where *a*_3_ = 1 – (*a*_1_ + *a*_2_) and τ_pair_ is obtained as

35 $$\tau_{\mathrm{pair}}=a_{1}b_{1}+a_{2}b_{2}+a_{3}b_{3}$$

Pair correlation lifetimes (PCL) are typically shorter than cage correlation lifetimes. The correlation function (eq 32) decays rapidly in the first few time steps, which can be problematic when an exponential function (eq 34) is used for fitting. To overcome this issue, the first data point is excluded from the fitting process, which allows for a more balanced fit without any significant impact on the final value of the pair correlation lifetime extracted from the data. As the pair can reform, MDTransport reports intermittent pair correlation lifetimes. PCL calculations (S2.10 of Supporting Information) are carried out by invoking the --pcl and --resname flags.

### Ion-Transport Mechanism

Understanding the transport mechanism in ionic solutions provides vital information regarding whether ions move via vehicular diffusion or structural hopping. These regimes can be distinguished by comparing the characteristic diffusional length to the radius of the solvation shell. For a central species *i* surrounded by coordinating molecules of species *j*, the diffusional length, *l*_D,ij_, represents the average distance species *i* migrates before its original solvation shell relaxes. This length is derived from the self-diffusion coefficient *D*_*i*_ and the residence time τ_ij_, using the relation $l_{D,\mathrm{ij}}^{d}=\sqrt{6\tau_{\mathrm{ij}}D_{i}}$ .^55,56^ By comparing *l*_D,ij_ to the radius of the first solvation shell, *l*_s_ (derived from the first minimum of the radial distribution function or explicitly provided via --rcut), the dominant transport mechanism can be classified as follows

36 $$\mathrm{mechanism}=\{\begin{array}{ll} l_{D,\mathrm{ij}}^{d}>l_{s} & \mathrm{vehicular}\;\mathrm{motion}\\ l_{D,\mathrm{ij}}^{d}\approx l_{s} & \mathrm{hopping}\;\mathrm{diffusion}\\ l_{D,\mathrm{ij}}^{d}<l_{s} & \mathrm{structural}\;\mathrm{motion} \end{array}$$

To accurately evaluate when the solvation shell relaxes, MDTransport calculates an intermittent pairwise correlation function, *C_ij_*. Rather than requiring the multiparticle geometry of the entire initial solvation shell to remain perfectly rigid, a condition that is overly restrictive and tracked separately via cage correlation, the vehicular analysis evaluates the residency of independent *i* – *j* pairs. In this framework, the Heaviside step function *h*(*t*) is defined such that *h*(*t*) = 1 as long as a specific target molecule *j* remains within the defined solvation shell (*l*_s_) of the reference ion *i*. By independently tracking whether individual target species remain bound to the reference species, regardless of the transient fluctuations of other surrounding molecules, the integral of *C_ij_* yields τ*_ij_*.^55,56^ To evaluate the vehicular transport mechanism, the --veh flag must be specified in conjunction with the --resname flag. The spatial boundary of the solvation shell can be defined manually using the --rcut parameter; otherwise, MDTransport employs a built-in routine to dynamically determine *r*_cut_ from the calculated RDF of the targeted species pair specified in the --resname flag. Please refer to S2.11 of Supporting Information for the command line reference.

### Radial Distribution Function Calculation (RDF)

MDTransport computes the radial distribution function using the following expression^14^

37 $$g_{\mathrm{ij}}\left(r\right)=\frac{V}{N_{i}N_{j}4\pi r^{2}\Delta r}\sum_{i=1}^{N_{i}}\sum_{j=1}^{N_{j}}\delta \left(r-r_{\mathrm{ij}}\right)$$

where Δ*r* represents the radial bin width, *N*_*i*_ and *N*_*j*_ denote the number of *i* ^th^ and *j* ^th^ species, respectively, and *r*_*ij*_ signifies the distance between molecules *i* and *j*. The Kronecker δ function takes a value of unity if *r*_*ij*_ falls within the bin *r*, *r* + Δ*r*; it is zero otherwise. MDTransport is capable of computing COM-COM RDFs as well as those between atoms and groups. To compute RDFs, it is initiated using the --rdf flag. By default, MDTransport evaluates the COM-COM RDFs across all possible combinations of species present in the system. To reduce computational overhead or isolate specific intermolecular interactions, users can restrict the calculation to targeted pairs by providing the --resname argument (e.g., --resname EMI BF or --resname EMI BF EMI NSC). Beyond standard COM-based calculations, it also supports atom–atom and atom–COM RDFs. These specific interaction modes can be executed by invoking the --rdf_mode flag in conjunction with the --rdf command, requiring the user to explicitly define the target atomic or molecular sites using the --rdf_pairs flag. Furthermore, users can append the --ana flag to automatically analyze the generated RDF curves. This feature seamlessly extracts key structural metrics for each specified pair, including the first peak radius, peak height, the first solvation shell radius, and the coordination number. S2.12 of Supporting Information provides a number of options for RDF calculations.

## Usage and Performance

### Download and Installation

To maximize accessibility for diverse user bases, MDTransport offers multiple deployment environments. A cloud-based web interface, a desktop application, and the source code are accessible through the Shah Research Group website at https://shahresearchgroup.org, enabling both browser-based and local execution. For development and version tracking, the source code is also hosted at https://github.com/ShahResearchGroup/MDTransport. The core library can be installed via standard package managers using PyPI (pip install MDTransport) or Conda (conda install -c conda-forge mdtransport). For development purposes, it can be compiled from the local directory (pip install -e.). Comprehensive setup instructions are detailed in Section S3 of the Supporting Information and the official documentation.

### Prerequisites

MDTransport is developed in Python and requires scientific libraries to function, specifically numpy, scipy, pandas, matplotlib, and tqdm. When processing LAMMPS trajectories, users must supply a trajectory file (e.g., lammpstrj) generated in real units. The MDTransport assumes an orthorhombic simulation box with periodic boundary conditions, computing the volume as *V* = *L*_*x*_ *L*_*y*_ *L*_*z*_. System topology and atomic charges are primarily extracted from the LAMMPS data file when utilizing the --mda, --chem, or --ovito parsers. Alternatively, if the native --lamp parser is invoked, charges can be extracted directly from the lammpstrj file, provided the q variable, which stores atomic charges in lammpstrj file, was included in the LAMMPS dump command. Further, to account for instances where charges in the data file are missing such as when charges are defined via the set command in the input script, MDTransport features an optional input script parser. This utility scans for user-defined charges and maps them to the respective atom types, ensuring accurate calculations of charge-dependent transport properties in automated workflows.

Users can also override partial charges defined in the force-field potential by using --species_charges flag if they need to use different theoretical frameworks for charge transport, such as the principle of topological quantization reported by Grasselli and Baroni.^57^ When enabled, the software maps all nonzero species charges to a user-defined magnitude (e.g., --species_charges 1) while preserving the original signs or specifying species charges individually (e.g., --species_charges EMI 1 BF −1), effectively bypassing the partial charges defined in the force field. Moreover, the calculation of integrated correlation times (e.g., eq 30 or eq 34) is sensitive to the temporal resolution of the stored trajectory. If trajectories are generated at large time intervals, fast dynamical modes may not be captured, which can lead to an artificial increase in the estimated correlation times. To minimize this effect, trajectory data used for time-correlation and residence time analyses should be recorded at intervals of 1 ps or smaller.

### Usage

Users can either type the full command with all input parameters or simply run mdt or python mdt.py and follow the on-screen options. This allows users to choose between executing specific commands directly or navigating through the guided prompts for a more interactive experience. We encourage users to start with interactive features of MDTransport in the beginning and transition to the command-based interface as the user gains experience working with MDTransport. When executing analyses, it is critical to properly define the time intervals. All parameters associated with time or trajectory frames must be specified in ps (see Table 1).

**Table 1:** Summary of Temporal Input Flags and Their Physical Meanings

flag	physical meaning and application
--t_steps	**Time step:** The time interval between consecutive trajectory frames for GROMACS in ps, or the integration time step for LAMMPS in fs.
--n_steps	**Trajectory range:** Defines the specific temporal portion of the simulation trajectory to be processed by MDTransport.
--t_window	**Slope evaluation window:** The specific duration over which the slope is calculated for transport properties (e.g., Einstein conductivity, Onsager transport coefficients, ion–ion correlations, spatial decomposition analysis).
--n_delta	**Maximum sliding window:** The maximum time interval (Δ*t*) used to perform time averaging across multiple time origins.
--min_window	**Minimum fitting window:** The minimum time window used to evaluate the log–log plot of MSD versus *t*, aiding in the isolation of the linear diffusive regime for diffusion calculations.

#### Running with LAMMPS

The --t_steps flag represents the actual time integration step used in the LAMMPS simulation when running the LAMMPS analysis. When using MDAnalysis, Chemfiles, or OVITO for parsing a LAMMPS trajectory, the user needs to provide the value of --dump_frame as it may fail to obtain the information regarding the time interval between the frames directly from the trajectory. Because MDTransport processes the output trajectory, the user simply provides the LAMMPS time step (--t_steps) and the dump output frequency (--dump_frame), and MDTransport internally calculates dt by multiplying these two values. For instance, an input time integration of 1 fs and a dump interval of 1000 yields an effective dt of 1 ps for the analysis.

#### Running with GROMACS

For GROMACS analyses, --t_steps is equivalent to the -dt flag used in GROMACS gmx commands. MDTransport uses the nearest dt value if the snapshots interval is not available in the GROMACS trajectory, which occurs when the value supplied for the --t_steps argument is greater than the time interval between two successive frames in the trajectory file. For example, if --t_steps is 30 ps, while the trajectory is available at a 20 ps interval, then MDTransport will read snapshots spaced 60 ps apart, i.e., every third configuration.

#### Memory, Storage, and Multiprocessing

To reduce memory and storage requirements, MDAnalysis, Chemfiles, or OVITO can be used to process GROMACS binary trajectory files such as .trr, .xtc, and binary input files (.tpr) and LAMMPS .lammpstrj, .xtc, and .data, eliminating the need to generate or convert files into ASCII format potentially requiring extra storage and resulting in loss of precision. In addition, MDTransport provides an optional disk-based processing mode (enabled via --disk flag), which significantly reduces memory usage by streaming intermediate atomic and COM data directly to the disk. This can lower peak memory consumption significantly, particularly for large-scale trajectory analysis. However, this mode may introduce a moderate performance overhead depending on the underlying storage hardware (e.g., HDD, SSD, or NVMe), and thus it represents a trade-off between memory efficiency and computational speed. MDTransport also features the capability to utilize multiple cores, if available. By default, the analysis utilizes all available CPU cores on the system and stores atomic or COM data in memory. The number of cores can be manually specified by the user via the --n_cores argument. MDTransport is also flexible in the way the process initialization method is controlled (e.g., spawn, fork, or forksever) via --mp_mode. This feature may be useful in a resource-constrained environment to optimize memory usage and minimize overheads associated with the initialization method.

### Feature Comparison across Tools

To highlight the capabilities of MDTransport, we compare its property estimation capabilities with popular MD packages. MDTransport supports all major transport-property calculations as shown in Table 2. While Table 2 highlights few prominent analysis packages, the field has recently expanded with various specialized tools and improved algorithms for transport-property estimation. For instance, tools such as Kinisi^27,58^ and STACIE^59^ provide advanced statistical frameworks for calculating diffusion and ionic conductivity with a focus on rigorous error estimation and automated convergence analysis. Similarly, tidynamics^17,60^ and freud^61^ offer high-performance implementations of fundamental dynamic correlations using fast Fourier transforms (FFT) for optimized scaling. Furthermore, specialized packages like KUTE,^62^ SporTran,^63^ and OTCP^64^ introduce sophisticated techniques for Green–Kubo and Einstein relations, specifically targeting noise reduction, spectral analysis, and improved convergence for challenging properties like thermal conductivity and shear viscosity. While these existing tools offer excellent performance and algorithmic depth in their respective domains, MDTransport distinguishes itself by providing a unified, user-accessible framework. Instead of requiring users to switch between disparate codes for different properties, MDTransport integrates these varied calculations into a single, high-throughput workflow. Furthermore, by leveraging established trajectory parsers (e.g., MDAnalysis, Chemfiles, and Ovito), MDTransport ensures seamless compatibility with almost all file types generated from both GROMACS and LAMMPS, making advanced transport analysis more accessible to the broader computational materials science community.

**Table 2:** Comparison of Transport-Property Calculation Capabilities between Mdtransport and Common MD Packages Including GROMACS,^7^ PyLAT,^14^ STACIE,^59^ and Travis^15^

property	MDTransport	GROMACS	MDAnalysis	PyLAT	STACIE	Travis
MSD and Self-diffusion coefficient	√	√	√	√	√	√
Ionic conductivity (Green–Kubo)		√		√	√	
Ionic conductivity (Nernst–Einstein)	√			√		
Ionic conductivity (Einstein)	√				√	
Viscosity (Green–Kubo)		√		√	√	
Onsager transport coeff.	√					
Spatial decomposition of correlations	√					
Ion-ion correlations	√					
Stefan-Maxwell diffusivity	√					
Vehicular Transport Mechanism	√					
Dielectric constant		√	√	√		
Pair correlation lifetime	√			√		√
Cage correlation lifetime	√					
Cluster population analysis	√	√				√
Density	√	√	√			√
RDF	√	√	√	√		√

### Computational Performance

Figure 3 illustrates the comparison of the postprocessing performance tested across various hardware and software environments. A trajectory of 100 ns for the system comprising 500 ion pairs of 1-ethyl-3-methylimidazolium [C_2_mim] tetrafluoroborate [BF_4_] was analyzed for the calculation of the Einstein ionic conductivity. Simulation details are provided in section S4 of Supporting Information. Figure 3(a) depicts the comparison of postprocessing times of a trajectory file containing 20001 snapshots in the .xtc format with one and eight cores. As expected, the performance is typically faster with higher number of cores, but the exact factor of improvement may vary based on the tools used, specifically across MDAnalysis, Chemfiles, or OVITO. Figure 3(b) illustrates the comparison of postprocessing times for a trajectory file containing 20001 snapshots in the .pdb format. It may be observed that, generally, analysis performed on the files in the pdb format is slower than that on the files in the xtc format. Further, the improvement in processing speed with higher number of cores is also slightly correspondingly less when pdb file format is employed. Figure 3(c) represents the comparison of processing times between LAMMPS and GROMACS trajectories containing 100001 snapshots. Postprocessing of LAMMPS and GROMACS files shows comparable performance, with only minor differences arising from the initial setup of the trajectory data structures, such as the MDAnalysis Universe object. Figure 3(d) presents a two-way comparison in the postprocessing times of trajectories with different numbers of snapshots on different hardware platforms. Further, it was observed that the calculations ran substantially faster on MACs as compared to Intel and AMD-based processors with --n_cores = 8 for a large file. Notably, .pdb files can be efficiently processed using the PDB mode, which is optimized for handling snapshots with multiprocessing.

**Figure 3:**
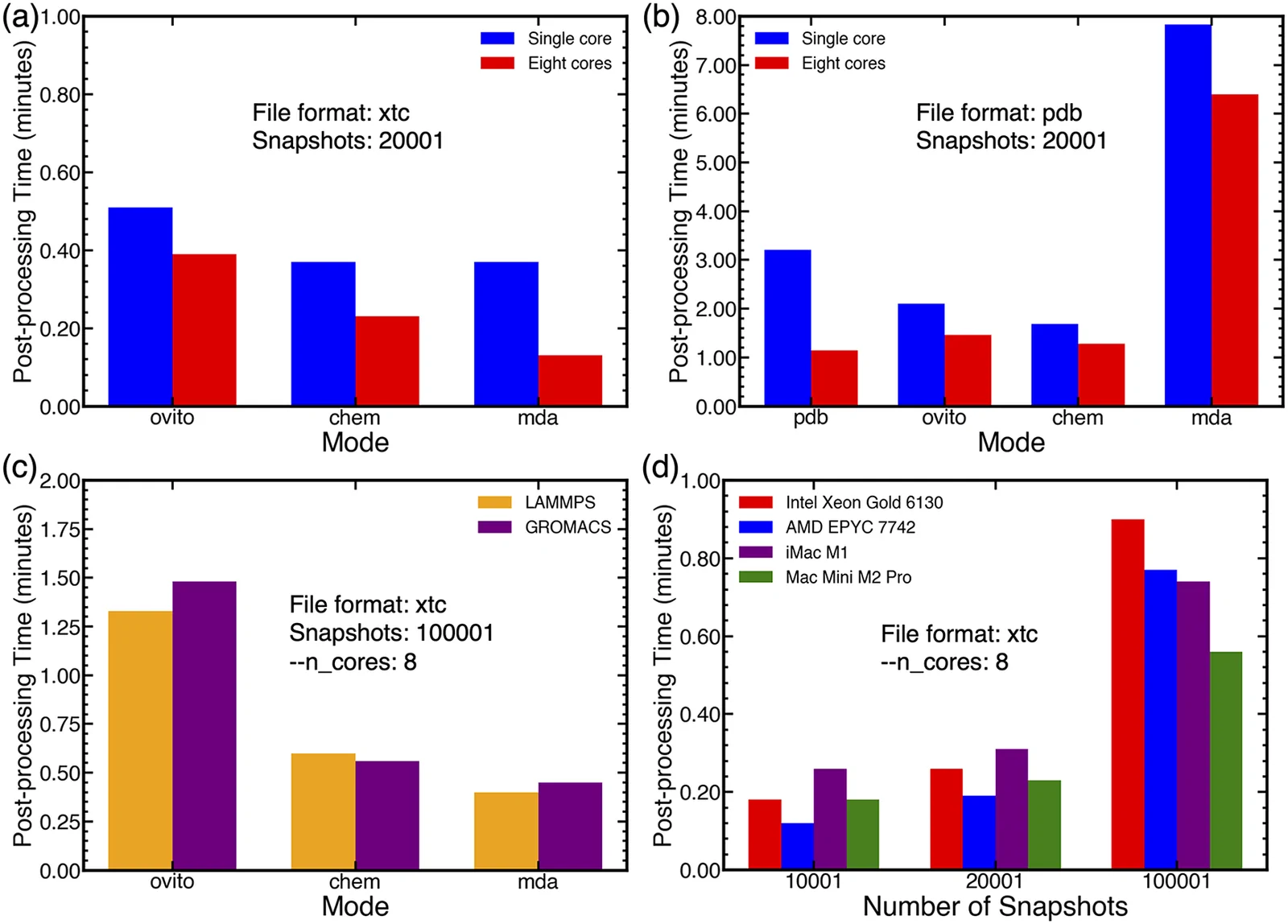
Comparison of postprocessing time for calculating the Einstein ionic conductivity of a 500 ion-pair [C_2_mim]­[BF_4_] system across a 100 ns simulation trajectory. To execute this analysis, MDTransport utilizes various backend parsers to read the trajectory files and extract data (e.g., COM) prior to property calculations. Performance is compared across different file formats, parsers, and hardware: (a) total 20001 snapshots stored at an interval of 5 ps, (b) pdb file with 20001 snapshots stored at an interval of 5 ps, (c) xtc file with 20001 snapshots stored at an interval of 5 ps, and (d) evaluating time scaling by varying the number of snapshots utilizing the mda mode. The backend parsers include pdb (a custom-built-in parser developed specifically for PDB files), chem (the Chemfiles library), mda (the MDAnalysis library), and ovito (the OVITO Python module).

Trajectory files e.g., .xtc from GROMACS or LAMMPS are best handled with MDAnalysis. Benchmark results (Figure 4) demonstrate that for text-based formats such as .lammpstraj and .pdb, Chemfiles and OVITO offer the most efficient processing. Chemfiles provides the lowest memory footprint for both .pdb (0.50 GB) and .lammpstrj (0.43 GB) files, while also delivering the fastest parsing for .pdb (1.52 min). OVITO remains the most time-efficient for .lammpstrj trajectories (2.14 min). In contrast, binary formats (.xtc and .trr) are processed significantly faster by Chemfiles and MDAnalysis, with Chemfiles achieving the leading time of 0.14 min. However, MDAnalysis achieves the lowest peak memory usage for these binary trajectories (0.55 GB), outperforming both Chemfiles and OVITO in memory efficiency for compressed formats. Further, disk-backed storage option offloads extracted coordinate data to temporary disk storage rather than maintaining the entire data in RAM, reducing peak memory requirements by approximately 50% compared to standard in-memory processing (Figure 4(c,d)). While execution times remain comparable for typical systems, the increased I/O overhead may result in longer processing times for exceptionally large systems due to disk read latency as shown in Figure 4(c). Furthermore, as memory overhead scales with the number of parallel workers during process initialization, users in extremely resource-constrained environments can further minimize the RAM footprint by opting for single-core execution. Irrespective of the mode, number of cores, or the architecture adopted for the analysis, MDTransport takes only a fraction of a minute to a couple of minutes for analysis.

**Figure 4:**
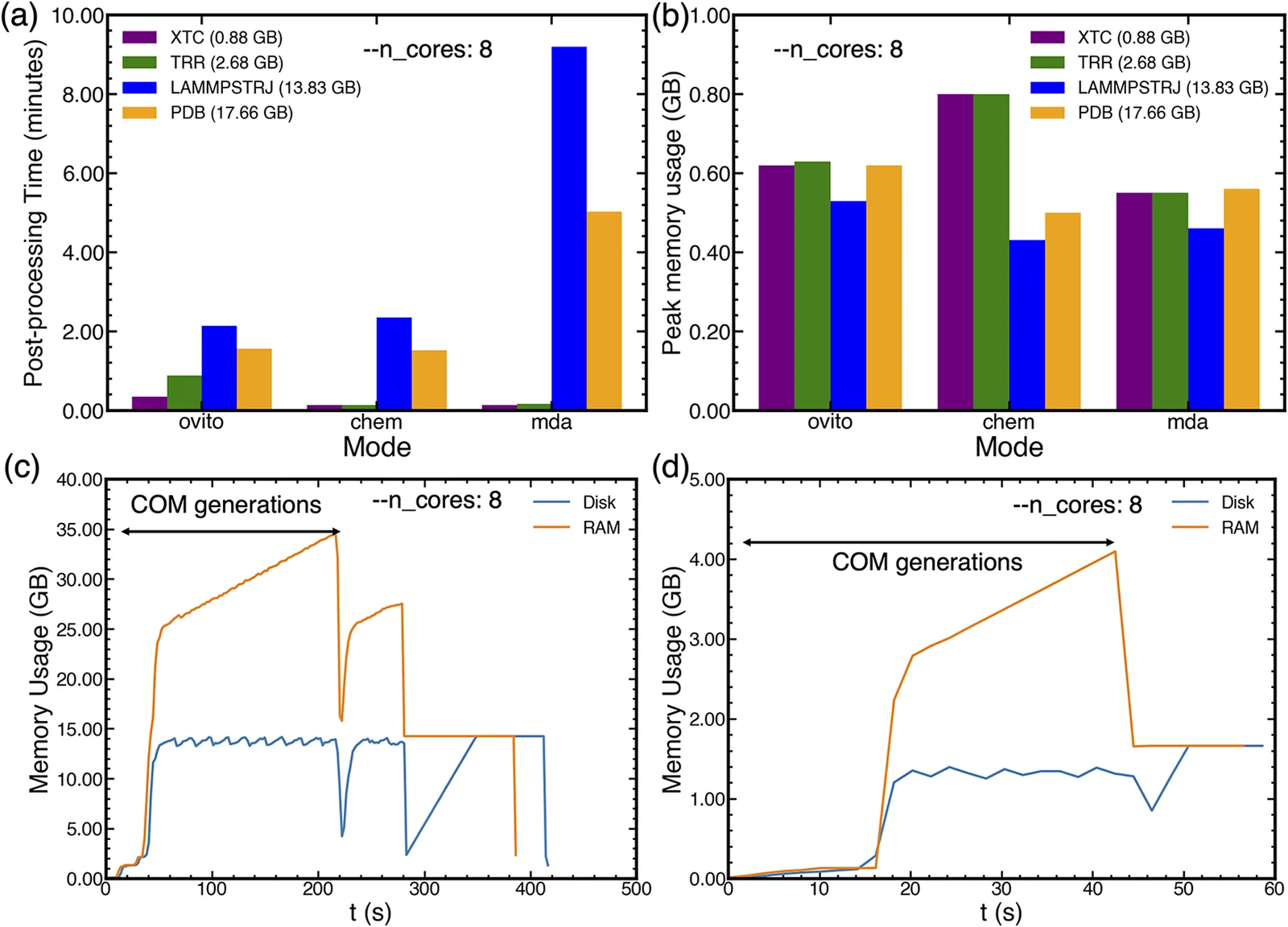
Computational performance benchmarks across different trajectory file formats: (a) Total postprocessing execution time (20001 frames) in minutes for OVITO, Chemfiles, and MDAnalysis, (b) peak memory consumption (20001 frames) in GB, (c) total memory usage with postprocessing ionic liquids system consisting of 1 million atoms (10001 frames), showing both in-memory and disk-based modes, and (d) total memory usage with postprocessing ionic liquids system consisting of 12500 atoms (100001 frames), comparing in-memory and disk-based modes. Benchmarks shown in panels (a) and (b) were performed on a Mac Mini M2 Pro, whereas the analyses in panels (c) and (d) were executed on an Intel Xeon Gold 6130 processor.

## Conclusion

MDTransport tool is an open-source, Python-based framework designed for the analysis of molecular dynamics trajectories generated by GROMACS and LAMMPS. It facilitates the computation of various transport properties such as self-diffusion coefficients, Onsager transport coefficients, Nernst–Einstein and Einstein conductivity, transference numbers, self- and cross-correlations, spatial decomposition of cross-correlations, pair and cage correlations lifetime, cluster analysis, and transport mechanism. MDTransport implements standardized best-practice methodologies to minimize user bias, thereby enhancing the reproducibility and reducing the uncertainty in results. This feature makes it particularly well-suited for high-throughput screening studies of a wide range of materials. Further development is underway to incorporate alternative methods to compute transport properties efficiently. Additionally, future work will involve introducing functionality by integrating additional transport properties and extending postprocessing capabilities to *ab initio* molecular dynamics packages such as CP2K.

## Supplementary Material







## Data Availability

MDTransport is an open-source code released under the GNU General Public License and is provided in Supporting Information. Sample trajectories and input files are uploaded in the Supporting Information. The code can also be accessed via github.com/ShahResearchGroup/MDTransport.

## Notes

MDTransport source code (ZIP)
Details of the installation guide, argument flags, commands usage, simulation details, validation, and transport properties (PDF)
Sample trajectories and input files (ZIP)

## References

[1] Brodholt J. P. (1998). Molecular
dynamics simulations of aqueous NaCl solutions at high pressures and
temperatures. Chem. Geol..

[2] Köddermann T., Paschek D., Ludwig R. (2007). Molecular
dynamic simulations of
ionic liquids: A reliable description of structure, thermodynamics
and dynamics. ChemPhysChem.

[3] Doherty B., Acevedo O. (2018). OPLS force field for
choline chloride-based deep eutectic
solvents. J. Phys. Chem. B.

[4] Thorat A., Verma A. K., Chauhan R., Sartape R., Singh M. R., Shah J. K. (2025). Identifying High
Ionic Conductivity Compositions of
Ionic Liquid Electrolytes Using Features of the Solvation Environment. J. Chem. Theory Comput..

[5] Verma A. K., Thorat A. S., Shah J. K. (2025). Predicting Ionic
Conductivity of
Imidazolium-Based Ionic Liquid Mixtures Using Quantum-Mechanically
Derived Partial Charges in the Condensed Phase. J. Phys. Chem. B.

[6] Maginn E. J., Messerly R. A., Carlson D. J., Roe D. R., Elliot J. R. (2019). Best practices
for computing transport properties 1. Self-diffusivity and viscosity
from equilibrium molecular dynamics [article v1. 0]. Living Journal of Computational Molecular Science.

[7] Abraham M. J., Murtola T., Schulz R., Páll S., Smith J. C., Hess B., Lindahl E. (2015). GROMACS: High
performance
molecular simulations through multi-level parallelism from laptops
to supercomputers. SoftwareX.

[8] Thompson A. P., Aktulga H. M., Berger R., Bolintineanu D. S., Brown W. M., Crozier P. S., In’t
Veld P. J., Kohlmeyer A., Moore S. G., Nguyen T. D. (2022). LAMMPS-a
flexible simulation tool for particle-based materials modeling at
the atomic, meso, and continuum scales. Comput.
Phys. Commun..

[9] Case D. A., Cheatham T. E., Darden T., Gohlke H., Luo R., Merz K. M., Onufriev A., Simmerling C., Wang B., Woods R. J. (2005). The Amber biomolecular simulation
programs. J. Comput. Chem..

[10] Brooks B. R., Brooks C. L., Mackerell A. D., Nilsson L., Petrella R. J., Roux B., Won Y., Archontis G., Bartels C., Boresch S. (2009). CHARMM:
the biomolecular simulation program. J. Comput.
Chem..

[11] Phillips J.
C., Braun R., Wang W., Gumbart J., Tajkhorshid E., Villa E., Chipot C., Skeel R. D., Kale L., Schulten K. (2005). Scalable molecular dynamics with NAMD. J. Comput. Chem..

[12] Smith W., Yong C., Rodger P. (2002). DL_POLY: Application to molecular
simulation. Mol. Simul..

[13] Eastman P., Galvelis R., Peláez R. P., Abreu C. R., Farr S. E., Gallicchio E., Gorenko A., Henry M. M., Hu F., Huang J. (2024). OpenMM 8: molecular dynamics simulation with machine
learning potentials. J. Phys. Chem. B.

[14] Humbert M. T., Zhang Y., Maginn E. J. (2019). PyLAT:
Python LAMMPS analysis tools. J. Chem. Inf.
Model..

[15] Brehm M., Kirchner B. (2012). TRAVIS-a free analyzer and visualizer for Monte Carlo
and molecular dynamics trajectories. J. Cheminf..

[16] Ivanova A., Mokshyna O., Polishchuk P. (2024). StreaMD: the
toolkit for high-throughput
molecular dynamics simulations. J. Cheminf..

[17] Róg T., Murzyn K., Hinsen K., Kneller G. R. (2003). nMoldyn:
a program
package for a neutron scattering oriented analysis of molecular dynamics
simulations. J. Comput. Chem..

[18] Walter N. P., Jaiswal A., Cai Z., Zhang Y. (2018). LiquidLib: A comprehensive
toolbox for analyzing classical and ab initio molecular dynamics simulations
of liquids and liquid-like matter with applications to neutron scattering
experiments. Comput. Phys. Commun..

[19] Michaud-Agrawal N., Denning E. J., Woolf T. B., Beckstein O. (2011). MDAnalysis:
a toolkit for the analysis of molecular dynamics simulations. J. Comput. Chem..

[20] McGibbon R. T., Beauchamp K. A., Harrigan M. P., Klein C., Swails J. M., Hernández C. X., Schwantes C. R., Wang L.-P., Lane T. J., Pande V. S. (2015). MDTraj: a modern open library for the analysis of molecular
dynamics trajectories. Biophys. J..

[21] Fraux, G. ; Fine, J. ; Barletta, G. P. ; Scalfi, L. ; Dimura, M. chemfiles/chemfiles: Version 0.9. 3. Zenodo, 2020.

[22] Stukowski A. (2010). Visualization
and analysis of atomistic simulation data with OVITO–the Open
Visualization Tool. Modell. Simul. Mater. Sci.
Eng..

[23] Humphrey W., Dalke A., Schulten K. (1996). VMD: visual
molecular dynamics. J. Mol. Graphics.

[24] Verma A. K., Thorat A. S., Shah J. K. (2024). Estimating
ionic conductivity of
ionic liquids: Nernst–Einstein and Einstein formalisms. Journal of Ionic Liquids.

[25] Hakim L., Ishii Y., Matubayasi N. (2021). Spatial-decomposition
analysis of
electrical conductivity in mixtures of ionic liquid and sodium salt
for sodium-ion battery electrolytes. J. Phys.
Chem. B.

[26] Tu K.-M., Ishizuka R., Matubayasi N. (2014). Spatial-decomposition analysis of
electrical conductivity in concentrated electrolyte solution. J. Chem. Phys..

[27] McCluskey A. R., Coles S. W., Morgan B. J. (2025). Accurate
estimation of diffusion
coefficients and their uncertainties from computer simulation. J. Chem. Theory Comput..

[28] Fong K. D., Self J., McCloskey B. D., Persson K. A. (2021). Ion correlations
and their impact on transport in polymer-based electrolytes. Macromolecules.

[29] Wang J., Hou T. (2011). Application of molecular dynamics
simulations in molecular property
prediction II: diffusion coefficient. J. Comput.
Chem..

[30] Li Y., Ni H. (2023). MD2D: A python module
for accurate determination of diffusion coefficient
from molecular dynamics. Comput. Phys. Commun..

[31] Ghaffarizadeh S. A., Wang G. J. (2024). A Picture is Worth
a Thousand Timesteps: Excess Entropy
Scaling for Rapid Estimation of Diffusion Coefficients in Molecular-Dynamics
Simulations of Fluids. J. Chem. Theory Comput..

[32] Baba H., Urano R., Nagai T., Okazaki S. (2022). Prediction of self-diffusion
coefficients of chemically diverse pure liquids by all-atom molecular
dynamics simulations. J. Comput. Chem..

[33] dos
Santos T. J., Abreu C. R., Horta B. A., Tavares F. W. (2020). Self-diffusion
coefficients of methane/n-hexane mixtures at high pressures: An evaluation
of the finite-size effect and a comparison of force fields. J. Supercrit. Fluids.

[34] Frömbgen T., Zaby P., Alizadeh V., Da Silva J. L., Kirchner B., Lourenço T. C. (2025). Lessons learned on obtaining reliable dynamic properties
for ionic liquids. ChemPhysChem.

[35] Moustafa S. G., Schultz A. J., Douglas J. F. (2024). Efficient single-run implementation
of generalized Einstein relation to compute transport coefficients:
A binary-based time sampling. J. Chem. Phys..

[36] Bullerjahn J. T., Von Bülow S., Hummer G. (2020). Optimal estimates of self-diffusion
coefficients from molecular dynamics simulations. J. Chem. Phys..

[37] Gullbrekken Ø., Røe I. T., Selbach S. M., Schnell S. K. (2023). Charge transport
in water–NaCl electrolytes with molecular dynamics simulations. J. Phys. Chem. B.

[38] France-Lanord A., Grossman J. C. (2019). Correlations from ion pairing and
the Nernst-Einstein
equation. Phys. Rev. Lett..

[39] Kubisiak P., Eilmes A. (2020). Estimates of electrical
conductivity from molecular
dynamics simulations: how to invest the computational effort. J. Phys. Chem. B.

[40] Blazquez S., Abascal J. L., Lagerweij J., Habibi P., Dey P., Vlugt T. J., Moultos O. A., Vega C. (2023). Computation of electrical
conductivities of aqueous electrolyte solutions: Two surfaces, one
property. J. Chem. Theory Comput..

[41] Tong J., Liang X., von Solms N., Huo F., Zhuang B. (2024). Ion correlations
in quaternary ionic liquids electrolytes. Journal
of Physics: Materials.

[42] Vargas-Barbosa N. M., Roling B. (2020). Dynamic ion correlations in solid and liquid electrolytes:
how do they affect charge and mass transport?. ChemElectroChem..

[43] Matubayasi N. (2019). Spatial-Decomposition
Analysis of Electrical Conductivity. Chem. Rec..

[44] Kapoor U., Shah J. K. (2016). Preferential ionic
interactions and microscopic structural
changes drive nonideality in binary ionic liquid mixtures as revealed
from molecular simulations. Ind. Eng. Chem.
Res..

[45] Thorat A., Chauhan R., Sartape R., Singh M. R., Shah J. K. (2024). Effect
of K^+^ Force Fields on Ionic Conductivity and Charge Dynamics
of KOH in Ethylene Glycol. J. Phys. Chem. B.

[46] Singh M. R., Singh P. G., Gande V. V., Chauhan R., Minocha N. (2024). Simplified
Universal Equations for Ionic Conductivity and Transference Number. J. Electrochem. Soc..

[47] Fong K. D., Self J., McCloskey B. D., Persson K. A. (2020). Onsager transport
coefficients and transference numbers in polyelectrolyte solutions
and polymerized ionic liquids. Macromolecules.

[48] Shao Y., Gudla H., Mindemark J., Brandell D., Zhang C. (2024). Ion transport
in polymer electrolytes: Building new bridges between experiment and
molecular simulation. Acc. Chem. Res..

[49] Gouverneur M., Schmidt F., Schönhoff M. (2018). Negative effective
Li transference
numbers in Li salt/ionic liquid mixtures: does Li drift in the “Wrong”
direction?. Phys. Chem. Chem. Phys..

[50] Zhang Z., Wheatle B. K., Krajniak J., Keith J. R., Ganesan V. (2020). Ion mobilities,
transference numbers, and inverse Haven ratios of polymeric ionic
liquids. ACS Macro Lett..

[51] Fong K. D., Self J., Diederichsen K. M., Wood B. M., McCloskey B. D., Persson K. A. (2019). Ion transport and
the true transference number in nonaqueous
polyelectrolyte solutions for lithium ion batteries. ACS Cent. Sci..

[52] Mistry A., Yu Z., Cheng L., Srinivasan V. (2023). On relative
importance of vehicular
and structural motions in defining electrolyte transport. J. Electrochem. Soc..

[53] Liu X., Vlugt T. J., Bardow A. (2011). Maxwell–stefan diffusivities
in binary mixtures of ionic liquids with dimethyl sulfoxide (dmso)
and h2o. J. Phys. Chem. B.

[54] Zhang Y., Maginn E. J. (2015). Direct correlation
between ionic liquid transport properties
and ion pair lifetimes: A molecular dynamics study. J. Phys. Chem. Lett..

[55] Self J., Fong K. D., Persson K. A. (2019). Transport in superconcentrated
LiPF_6_ and LiBF_4_/propylene carbonate electrolytes. ACS Energy Lett..

[56] Singh N., Kashyap H. K. (2025). Nonmonotonic Ionic Transport and
Its Correlation with
Structural and Dynamic Heterogeneities in NaTFSI and NaFSI-Based Aqueous
Electrolytes: Salt-in-Water versus Water-in-Salt. J. Phys. Chem. B.

[57] Grasselli F., Baroni S. (2019). Topological quantization
and gauge invariance of charge
transport in liquid insulators. Nat. Phys..

[58] Sasaki R., Tateyama Y., Searles D. J. (2025). Constant-current
nonequilibrium molecular
dynamics approach for accelerated computation of ionic conductivity
including ion-ion correlation. PRX Energy.

[59] Toraman G., Fauconnier D., Verstraelen T. (2025). STable AutoCorrelation Integral Estimator:
Robust and Accurate Transport Properties from Molecular Dynamics Simulations. J. Chem. Inf. Model..

[60] Kraft D. J., Wittkowski R., Ten Hagen B., Edmond K. V., Pine D. J., Löwen H. (2013). Brownian motion
and the hydrodynamic friction tensor
for colloidal particles of complex shape. Phys.
Rev. E.

[61] Ramasubramani V., Dice B. D., Harper E. S., Spellings M. P., Anderson J. A., Glotzer S. C. (2020). freud: A software
suite for high
throughput analysis of particle simulation data. Comput. Phys. Commun..

[62] Otero-Lema M., Lois-Cuns R., Boado M. A., Montes-Campos H., Mendez-Morales T., Varela L. M. (2025). KUTE: Green–Kubo uncertainty-based
transport coefficient estimator. J. Chem. Inf.
Model..

[63] Ercole L., Bertossa R., Bisacchi S., Baroni S. (2022). SporTran: A code to
estimate transport coefficients from the cepstral analysis of (multivariate)
current time series. Comput. Phys. Commun..

[64] Jamali S. H., Wolff L., Becker T. M., De Groen M., Ramdin M., Hartkamp R., Bardow A., Vlugt T. J., Moultos O. A. (2019). OCTP: A
tool for on-the-fly calculation of transport properties of fluids
with the order-n algorithm in LAMMPS. J. Chem.
Inf. Model..

