# Supplementary material for: MDTransport: A Modular, Open-Source, Extensible Python Tool for Computing Transport Properties from GROMACS and LAMMPS Simulations
Source: J Chem Inf Model. 2026 Aug 1;66(16):9989–10003. doi: 10.1021/acs.jcim.6c00558 (PMC13508777; doi:10.1021/acs.jcim.6c00558)
Supplement: Supplementary file 1 [file ci6c00558_si_002.pdf]

Supporting Information

MDTransport: A Modular, Open-Source, Extensible Python Tool for Computing Transport Properties from GROMACS and LAMMPS Simulations

Ashutosh Kumar Verma, Amey S. Thorat, and Jindal K Shah*

School of Chemical Engineering, Oklahoma State University, Stillwater, OK 74078, United States

E-mail: jindal.shah@okstate.edu

Contents

S1 Argument Flags **S4**

S1.1 General Analysis Modes S4

S1.2 GROMACS Trajectory Parser S4

S1.3 LAMMPS Trajectory Parser S4

S1.4 File & Path Arguments S4

S1.5 General Parameters S5

S1.6 Properties Calculations S5

S1.7 Analysis-Specific Parameters S6

S1.8 Analysis Type and Flag Requirements S7

S1.9 Common Parser S7

S2 Commands Usage **S8**

S2.1 MSD and Diffusion Analysis S8

S2.2 Nernst-Einstein Conductivity S10

S2.3 Ion-Ion Correlations S10

S2.4 Einstein Conductivity Using Einstein-Helfand Equation S10

S2.5 Spatial Decomposition Analysis of Ion-Ion Correlations S11

S2.6 Onsager Transport Coefficients S11

S2.7 Ionic Conductivity Using Stefan-Maxwell Diffusivity S11

S2.8 Cluster Analysis S12

S2.9 Cage Correlation Lifetime S12

S2.10 Pair Correlation Lifetime S12

S2.11 Transport Mechanism of Species S13

S2.12 Radial Distribution Function Calculation S13

S3 Installation Guide **S14**

S3.1 Create a Virtual Environment and Run MDTransport Directly S15

S3.2 Build From Sources S15

S3.3 Installation via Package Managers S16

S4 Simulation Details **S16**

S5 Validation **S17**

S6 Properties **S19**

S6.1 Mean Square Displacement S19

S6.2 Log MSD vs Log t curves S20

S6.3 Self-Diffusion Coefficients Calculations S21

S6.4 Collective Mean Square Displacement S22

S6.5 Ionic Conductivity Calculations S23

S6.6 Maxwell–Stefan Diffusion and Ionic Conductivity S25

S6.7 Cluster, cage and Ion-pairs Lifetime S26

S1 Argument Flags

S1.1 General Analysis Modes

```
[--gro] : Run GROMACS analysis.
[--lam] : Run LAMMPS analysis.
```

S1.2 GROMACS Trajectory Parser

```
[--pdb]   : Use PDB file to extract pecies data (e.g., --pdb).
[--exe]   : Use gmx to extract species data from GROMACS files (e.g., --exe gmx).
[--chem]  : Use Chemfiles to extract species data from GROMACS files (e.g., --chem).
[--mda]   : Use MDAnalysis to extract species data from GROMACS files (e.g., --mda).
[--ovito] : Use Ovito to extract species data from GROMACS files (e.g., --ovito).
```

S1.3 LAMMPS Trajectory Parser

```
[--lamp]  : Use lammpstrj file to extract species data (e.g., --traj /path/to/trajectory/file).
[--com]   : Use LAMMPS generated com.txt to extract species data (e.g., --com /path/to/com/file).
[--chem]  : Use Chemfiles to extract species data from LAMMPS files (e.g., --chem).
[--mda]   : Use MDAnalysis to extract species data from LAMMPS files (e.g., --mda).
[--ovito] : Use Ovito to extract species data from LAMMPS files (e.g., --ovito).
```

S1.4 File & Path Arguments

```
[--in]       : Path to MDTransport input file (e.g., --in /path/to/input/file).
[--itp]      : Directory containing GROMACS ITP files (e.g., --itp /path/to/itp/directory).
[--tpr]      : Path to GROMACS TPR file (e.g., --tpr /path/to/tpr/file).
[--traj]     : Path to trajectory file (e.g., --traj /path/to/trajectory/file).
[--data]     : Path to LAMMPS data file (e.g., --traj /path/to/data/file).
[--lam_in]   : Path to LAMMPS input file (e.g., --traj /path/to/input/file).
[--gro_path] : Path to GROMACS files directory (e.g., --traj /path/to/gromacs/files).
[--lam_path] : Path to LAMMPS files directory (e.g., --traj /path/to/lammps/files).
```

S1.5 General Parameters

```
[--temp]        : Temperature in Kelvin (e.g., --temp 298.0).
[--t_steps]     : Time steps in ps (e.g., --t_steps 2).
[--n_delta]     : Time window for time origins shifting in ps (e.g., --n_delta 1000).
[--t_window]    : Time window for slope calculations in ps (e.g., --t_window 1000).
[--min_window]  : Minimum time window for diffusivity calculations in ps (e.g., --min_window 1000).
[--n_steps]     : Snapshots range in ps (e.g., --n_steps 0 20000).
[--fit_method]  : MSD fitting method for diffusion calculations (e.g., --fit_method gls).
[--msd_fit]     : Time window (in ps) for MSD fitting (e.g., --msd_fit 10000 40000).
[--steps]       : Steps for time origine shifting (e.g., --steps 20).
[--dir]         : Specify directions for msd/diffusion (e.g., --dir x y z).
[--n]           : process every N-th frame (e.g., --n 5).
[--dump_frame]  : LAMMPS trajectory dumping frame (e.g., --dump_frame 1000).
[--n_cores]     : Number of CPU cores to use for parallel processing (e.g., --n_cores 8).
[--mp_mode]     : Multi-processing initialization (e.g., --mp_mode fork).
```

S1.6 Properties Calculations

```
[--msd]         : Compute mean squared displacement and diffusion (e.g., --msd).
[--corr]        : Calculate ion-ion correlations and Einstein conductivity (e.g., --corr).
[--onsg]        : Calculate Onsager transport coefficients (e.g., --onsg).
[--spda]        : Spatial decomposition of ion-ion correlations (e.g., --spda).
[--en]          : Calculate Einstein conductivity (e.g., --en).
[--ne]          : Calculate Nernst-Einstein conductivity (e.g., --ne).
[--smd]         : Run Stefan-Maxwell diffusivity analysis (e.g., --smd).
[--pcl]         : Calculate pair correlation lifetime (e.g., --pcl).
[--cage_p]      : Cage population analysis (e.g., --cage_p).
[--cage]        : Calculate cage correlation lifetime (e.g., --cage).
[--veh]         : Run transport mechanism analysis (e.g., --veh).
[--rdf]         : Calculate radial distribution functions (e.g., --rdf).
[--get_csv]     : Parse and save trajectories data. (e.g., --get_csv).
```

S1.7 Analysis-Specific Parameters

```
[--species]      : Define species names and counts for LAMMPS (e.g., --species EMI 500 BF4 500)
[--resname]      : Define species names (e.g., --resname EMI BF)
[--skip_species] : Exclude Resnames from COM generation (e.g., --skip_species SOL).
[--rcut]         : Cutoff radius for analysis (e.g., --rcut 7.6).
[--rbin]         : RDF bin size (e.g., --rbin 0.2)
[--ref_species]  : Reference species for cage correlation (e.g., --ref_species EMI).
[--cage_species] : Cage species for cage correlation (e.g., --cage_species BF).
[--ana]          : Run optional RDFs analysis (e.g., --ana).
[--eh]           : Enable Einstein-Helfand for CORR and ONSG analysis (e.g., --eh).
[--rdf_mode]     : Specify the RDF calculation mode (e.g., --rdf_mode com com)
[--rdf_pairs]    : Specify the RDF pairs.
                   (e.g., --rdf_pairs resname EMI type HR resname BF type F)
                   (e.g., --rdf_pairs type 1-6 type 7-12)
[--msd_mode]     : Specify the MSD calculation mode (e.g., --msd_mode com)
[--msd_species]  : Specify the MSD species.
                   (e.g., --msd_species resname EMI type HR resname BF type F)
                   (e.g., --msd_species type 1-6 type 7-12)
[--shell]        : Solvation shell choice for SPDA (e.g., --shell first).
[--species_charges] : Override net charges on each species (e.g., --species_charges 1 or
                    --species_charges EMI 0.8).
```

S1.8 Analysis Type and Flag Requirements

Analysis Type	Required Flags	Optional Flags
**MSD & Diffusion**	--msd, Parser, --t_steps	--dir, --species, --msd_fit, --fit_method, --msd_mode, --msd_species, --skip_species, --min_window, --n_steps
**Einstein, Self- & Cross- Correlations**	--corr, Parser, --t_steps, --temp	--dir, --eh, --skip_species, --n_delta, --t_window, --n_steps, --steps
**Onsager Transport Coefficient**	--onsg, Parser, --t_steps	--dir, --eh, --skip_species, --n_delta, --t_window, --n_steps, --steps
**Spatial Decomposition Analysis**	--spda, Parser, --t_steps, --temp	--dir, --rcut, --skip_species, --n_delta, --t_window, --n_steps, --steps
**Einstein conductivity**	--en, Parser, --t_steps, --temp	--dir, --skip_species, --t_window, --n_steps
**Nernst-Einstein conductivity**	--ne, Parser, --t_steps, --temp	--dir, --skip_species, --msd_fit, --fit_method, --min_window, --n_steps
**Stefan-Maxwell Diffusivity**	--smd, Parser, --t_steps, --temp	--dir, --skip_species, --min_window, --n_steps, --steps
**Pair-correlation Lifetime**	--pcl, Parser, --t_steps, --resname	--dir, --skip_species, --rcut, --n_steps, --steps
**Cage Population**	--cage_p, Parser, --t_steps, --resname	--skip_species, --rcut, --n_steps
**Cage-correlation Lifetime**	--cage, Parser, --t_steps, --ref_species/cage_species	--skip_species, --rcut, --n_steps, --steps
**Transport Mechanism**	--veh, Parser, --t_steps, --resname	--skip_species, --rcut, --n_steps, --min_window, --steps, --dir
**Radial Distribution Function**	--rdf, Parser, --t_steps	--resname, --skip_species, --n_steps, --steps, --msd_mode, --msd_pairs

S1.9 Common Parser

GROMACS

```
[Parser]  :[--pdb / --exe <gmx or gmx_mpi> / --mda / --chem / --ovito]
```

LAMMPS

```
[Parser]  :[--lamp / --com <com file> / --mda / --chem / --ovito]
```

S2 Commands Usage

S2.1 MSD and Diffusion Analysis

COM-based MSD for Selected Groups

GROMACS

```
mdt [--gro] [--msd] [Parser]
    [--t_steps <time steps>] [--min_window <min. time window>] [--dir <x, y, z>]
    [--msd_mode com --msd_species resname <resname> type <atom type> <atom type>]
    [--n_steps <start frame> <end frame>]
mdt [--gro] [--msd] [Parser]
    [--t_steps <time steps>] [--min_window <min. time window>] [--dir <x, y, z>]
    [--msd_mode com --msd_species resname <resname> name <atom name> <atom name>]
    [--n_steps <start frame> <end frame>]
mdt [--gro] [--msd] [Parser]
    [--t_steps <time steps>] [--min_window <min. time window>] [--dir <x, y, z>]
    [--msd_mode com --msd_species resname <resname> type <atom type> <atom type>
    resname <resname> type <atom type> <atom type>] [--n_steps <start frame> <end frame>]
mdt [--gro] [--msd] [Parser]
    [--t_steps <time steps>] [--min_window <min. time window>] [--dir <x, y, z>]
    [--msd_mode com --msd_species resname <resname> name <atom name> <atom name>
    resname <resname> name <atom name> <atom name>] [--n_steps <start frame> <end frame>]
```

LAMMPS

```
mdt [--lam] [--msd] [Parser]
    [--dump_frame <steps>] [--t_steps <time steps>] [--n <frame stride>] [--dir <x, y, z>]
    [--n_steps <start frame> <end frame>] [--min_window <min. time window>]
    [--msd_mode com --msd_species resname <resname> type 1 2]
mdt [--lam] [--msd] [Parser]
    [--dump_frame <steps>] [--t_steps <time steps>] [--n <frame stride>] [--dir <x, y, z>]
    [--n_steps <start frame> <end frame>] [--min_window <min. time window>]
    [--msd_mode com --msd_species resname <resname> type <atom type> <atom type> resname <resname>]
mdt [--lam] [--msd] [Parser]
    [--dump_frame <steps>] [--t_steps <time steps>] [--n <frame stride>] [--dir <x, y, z>]
    [--n_steps <start frame> <end frame>] [--min_window <min. time window>]
    [--msd_mode com --msd_species resname <resname> type <atom type> <atom type>
    resname <resname> type <atom type> <atom type>]
mdt [--lam] [--msd] [Parser]
    [--dump_frame <steps>] [--t_steps <time steps>] [--n <frame stride>] [--dir <x, y, z>]
    [--n_steps <start frame> <end frame>] [--min_window <min. time window>]
    [--msd_mode com --msd_species resname <resname> resname <resname>]
```

Atom-based MSD for Selected Atoms

GROMACS

```
mdt [--gro] [--msd] [Parser]
    [--t_steps <time steps>] [--min_window <min. time window>] [--dir <x, y, z>]
    [--msd_mode atom --msd_species resname <resname> type <atom type>]
    [--n_steps <start frame> <end frame>]
mdt [--gro] [--msd] [Parser]
    [--t_steps <time steps>] [--min_window <min. time window>] [--dir <x, y, z>]
    [--msd_mode atom --msd_species resname <resname> name <atom name>]
    [--n_steps <start frame> <end frame>]
mdt [--gro] [--msd] [Parser]
    [--t_steps <time steps>] [--min_window <min. time window>] [--dir <x, y, z>]
    [--msd_mode atom --msd_species resname <resname> type <atom type>
    resname <resname> type <atom type>] [--n_steps <start frame> <end frame>]
mdt [--gro] [--msd] [Parser]
    [--t_steps <time steps>] [--min_window <min. time window>] [--dir <x, y, z>]
    [--msd_mode atom --msd_species resname <resname> name <atom name>
    resname <resname> name <atom name>] [--n_steps <start frame> <end frame>]
```

LAMMPS

```
mdt [--lam] [--msd] [Parser]
    [--dump_frame <steps>] [--t_steps <time steps>] [--n <frame stride>] [--dir <x, y, z>]
    [--n_steps <start frame> <end frame>] [--min_window <min. time window>]
    [--msd_mode atom --msd_species resname <resname> type <atom type>]
mdt [--lam] [--msd] [Parser]
    [--dump_frame <steps>] [--t_steps <time steps>] [--n <frame stride>] [--dir <x, y, z>]
    [--n_steps <start frame> <end frame>] [--min_window <min. time window>]
    [--msd_mode atom --msd_species resname <resname> type <atom type> <atom type>]
mdt [--lam] [--msd] [Parser]
    [--dump_frame <steps>] [--t_steps <time steps>] [--n <frame stride>] [--dir <x, y, z>]
    [--n_steps <start frame> <end frame>] [--min_window <min. time window>]
    [--msd_mode atom --msd_species resname <resname> type <atom type> <resname> type <atom type>]
mdt [--lam] [--msd] [Parser]
    [--dump_frame <steps>] [--t_steps <time steps>] [--n <frame stride>] [--dir <x, y, z>]
    [--n_steps <start frame> <end frame>] [--min_window <min. time window>]
    [--msd_mode atom --msd_species resname <resname> <resname>]
```

COM-based MSD for Species

GROMACS

```
mdt [--gro] [--msd] [Parser]
    [--t_steps <time steps>] [--skip_species <resname>] [--dir <x, y, z>]
    [--n_steps <start frame> <end frame>] [--min_window <min. time window>]
```

LAMMPS

```
mdt [--lam] [--msd] [Parser]
    [--t_steps <time steps>] [--dir <x, y, z>] [--n <frame stride>] [--dump_frame <steps>]
    [--min_window <min. time window>] [--n_steps <start frame> <end frame>]
```

S2.2 Nernst-Einstein Conductivity

GROMACS

```
mdt [--gro] [--ne] [Parser]
    [--t_steps <time steps>] [--skip_species <resname>] [--temp <temperature>]
    [--n_steps <start frame> <end frame>] [--min_window <min. time window>]
```

LAMMPS

```
mdt [--lam] [--ne] [Parser]
    [--t_steps <time steps>] [--dump_frame <steps>] [--n <frame stride>] [--temp <temperature>]
    [--n_steps <start frame> <end frame>] [--min_window <min. time window>]
```

S2.3 Ion-Ion Correlations

GROMACS

```
mdt [--gro] [--corr] [Parser]
    [--t_steps <time steps>] [--n_delta <window for time origin shifting>] [--temp <temperature>]
    [--n_steps <start frame> <end frame>] [--t_window <time window for slope>]
```

LAMMPS

```
mdt [--lam] [--corr] [Parser]
    [--t_steps <time steps>] [--dump_frame <steps>] [--n_delta <window for time origin shifting>]
    [--n <frame stride>] [--n_steps <start frame> <end frame>] [--temp <temperature>]
    [--t_window <time window for slope>]
```

S2.4 Einstein Conductivity Using Einstein-Helfand Equation

GROMACS

```
mdt [--gro] [--en] [Parser]
    [--t_steps <time steps>] [--t_window <time window for slope>]
    [--n_steps <start frame> <end frame>] [--temp <temperature>]
```

LAMMPS

```
mdt [--lam] [--en] [Parser]
    [--t_steps <time steps>] [--dump_frame <steps>] [--t_window <time window for slope>]
    [--n <frame stride>] [--n_steps <start frame> <end frame>] [--temp <temperature>]
```

S2.5 Spatial Decomposition Analysis of Ion-Ion Correlations

GROMACS

```
mdt [--gro] [--spda] [Parser]
    [--t_steps <time steps>] [--n_delta <window for time origin shifting>] [--temp <temperature>]
    [--n_steps <start frame> <end frame>] [--t_window <time window for slope>]
```

LAMMPS

```
mdt [--lam] [--spda] [Parser]
    [--t_steps <time steps>] [--dump_frame <steps>] [--n_delta <window for time origin shifting>]
    [--n <frame stride>] [--n_steps <start frame> <end frame>] [--temp <temperature>]
    [--t_window <time window for slope>]
```

S2.6 Onsager Transport Coefficients

GROMACS

```
mdt [--gro] [--onsg] [Parser]
    [--t_steps <time steps>] [--t_window <time window for slope>] [--temp <temperature>]
    [--n_steps <start frame> <end frame>] [--n_delta <window for time origin shifting>]
```

LAMMPS

```
mdt [--lam] [--onsg] [Parser]
    [--t_steps <time steps>] [--dump_frame <steps>] [--temp <temperature>]
    [--n <frame stride>] [--t_steps <time steps>] [--t_window <time window for slope>]
    [--n_steps <start frame> <end frame>] [--n_delta <window for time origin shifting>]
```

S2.7 Ionic Conductivity Using Stefan-Maxwell Diffusivity

GROMACS

```
mdt [--gro] [--smd] [Parser]
    [--t_steps <time steps>] [--temp <temperature>]
    [--n_steps <start frame> <end frame>] [--min_window <min. time window>]
```

LAMMPS

```
mdt [--lam] [--smd] [Parser]
    [--t_steps <time steps>] [--dump_frame <steps>] [--n <frame stride>] [--temp <temperature>]
    [--n_steps <start frame> <end frame>] [--min_window <min. time window>]
```

S2.8 Cluster Analysis

GROMACS

```
mdt [--gro] [--cage_p] [Parser]
    [--t_steps <time steps>] [--n_steps <start frame> <end frame>] [--rcut <first solvation>]
    [--resname <reference species> <target species>]
```

LAMMPS

```
mdt [--lam] [--cage_p <reference species> <cage species>] [Parser]
    [--n <frame stride>] [--dump_frame <steps>] [--species <resname> <numbers>]
    [--rcut <first solvation>] [--n_steps <start frame> <end frame>] [--t_steps <time steps>]
    [--resname <reference species> <target species>]
```

S2.9 Cage Correlation Lifetime

GROMACS

```
mdt [--gro] [--cage] [Parser]
    [--ref_species <resname>] [--cage_species <resname>] [--n_steps <start frame> <end frame>]
    [--t_steps <time steps>] [--rcut <first solvation>] [--steps <time origin steps>]
```

LAMMPS

```
mdt [--lam] [--cage] [Parser]
    [--t_steps <time steps>] [--dump_frame <steps>] [--ref_species <resname>]
    [--cage_species <resname>] [--n_steps <start frame> <end frame>] [--rcut <first solvation>]
    [--steps <time origin steps>] [--n <frame stride>]
```

S2.10 Pair Correlation Lifetime

GROMACS

```
mdt [--gro] [--pcl] [Parser]
    [--t_steps <time steps>] [--n_steps <start frame> <end frame>] [--steps <time origin steps>]
    [--resname <reference species> <target species>]
```

LAMMPS

```
mdt [--lam] [--pcl] [Parser]
    [--dump_frame <steps>] [--t_steps <time steps>] [--species <resname> <numbers>]
    [--n <frame stride>] [--n_steps <start frame> <end frame>] [--steps <time origin steps>]
    [--resname <reference species> <target species>]
```

S2.11 Transport Mechanism of Species

GROMACS

```
mdt [--gro] [--veh] [Parser]
    [--t_steps <time steps>] [--min_window <min. time window>] [--rcut <first solvation>]
    [--temp <temperature>] [--steps <time origin steps>] [--n_steps <start frame> <end frame>]
    [--resname <reference species> <target species>]
```

LAMMPS

```
mdt [--lam] [--veh] [Parser]
    [--t_steps <time steps>] [--dump_frame <steps>] [--min_window <min. time window>]
    [--n <frame stride>] [--n_steps <start frame> <end frame>] [--temp <temperature>]
    [--rcut <first solvation>] [--steps <time origin steps>]
    [--resname <reference species> <target species>]
```

S2.12 Radial Distribution Function Calculation

COM-COM RDF

GROMACS

```
mdt [--gro] [--rdf] [Parser]
    [--t_steps <time steps>] [--n_steps <start frame> <end frame>] [--steps <time origin steps>]
mdt [--gro] [--rdf] [Parser]
    [--rdf_mode com com --rdf_pairs resname <resname> type <atom type> <atom type> resname
    <resname> type <atom type> <atom type>] [--t_steps <time steps>] [--n_steps <start frame>
    <end frame>]
mdt [--gro] [--rdf] [Parser]
    [--rdf_mode com com --rdf_pairs resname <resname> name <atom name> <atom name> resname
    <resname> name <atom name> <atom name>] [--t_steps <time steps>] [--n_steps <start frame>
    <end frame>]
```

LAMMPS

```
mdt [--lam] [--rdf] [Parser]
    [--n <frame stride>] [--t_steps <time steps>] [--n_steps <start frame> <end frame>]
    [--dump_frame <steps>] [--steps <time origin steps>]
mdt [--lam] [--rdf] [Parser]
    [--dump_frame <steps>] [--t_steps <time steps>] [--n_steps <start frame> <end frame>]
    [--n <frame stride>] [--rdf_mode com com --rdf_pairs resname <resname>
    type <atom type> <atom type> resname <resname> type <atom type> <atom type>]
```

ATOM-COM RDF

GROMACS

```
mdt [--gro] [--rdf] [Parser]
    [--rdf_mode atom com --rdf_pairs resname <resname> type <atom type> resname <resname> type
    <atom type> <atom type>] [--t_steps <time steps>] [--n_steps <start frame> <end frame>]
mdt [--gro] [--rdf] [Parser]
    [--rdf_mode atom com --rdf_pairs resname <resname> name <atom name> resname <resname> name
    <atom name> <atom name>] [--t_steps <time steps>] [--n_steps <start frame> <end frame>]
```

LAMMPS

```
mdt [--lam] [--rdf] [Parser]
    [--dump_frame <steps>] [--t_steps <time steps>] [--n_steps <start frame> <end frame>]
    [--n <frame stride>] [--rdf_mode atom com --rdf_pairs resname <resname> type <atom type>
    resname <resname> type <atom type> <atom type>]
```

ATOM-ATOM RDF

GROMACS

```
mdt [--gro] [--rdf] [Parser]
    [--rdf_mode atom atom --rdf_pairs resname <resname> type <atom type> resname <resname> type
    <atom type>] [--t_steps <time steps>] [--n_steps <start frame> <end frame>]
mdt [--gro] [--rdf] [Parser]
    [--rdf_mode atom atom --rdf_pairs resname <resname> name <atom name> resname <resname> type
    <atom name>] [--t_steps <time steps>] [--n_steps <start frame> <end frame>]
```

LAMMPS

```
mdt [--lam] [--rdf] [Parser]
    [--dump_frame <steps>] [--t_steps <time steps>] [--n_steps <start frame> <end frame>]
    [--n <frame stride>] [--rdf_mode atom atom --rdf_pairs resname <resname> type <atom type>
    resname <resname> type <atom type>]
```

S3 Installation Guide

The following dependencies are required:

```
python >=3.9
matplotlib >=3.6
pandas >=2.0
numpy >=1.20
scipy >=1.10
tqdm >=4.60
```

MDAnalysis Requirements

```
MDAnalysis is required only if you intend to use the --mda option.
Installation options:
# Recommended if using Conda
conda install -c conda-forge mdanalysis
# Alternative using pip
pip install MDAnalysis
```

Chemfiles Requirements

```
Chemfiles is required only if you intend to use the --chem option.
Installation options:
# Recommended if using Conda (Anaconda/Miniconda)
conda install -c conda-forge chemfiles
# Alternative using pip
pip install chemfiles
```

Ovito Requirements

```
Ovito is required only if you intend to use the --ovito option.
Installation options:
# Recommended if using Conda (Anaconda/Miniconda)
conda install -c conda-forge ovito
# Alternative using pip
pip install ovito
```

S3.1 Create a Virtual Environment and Run MDTransport Directly

```
git clone https://github.com/ShahResearchGroup/MDTransport.git
cd MDTransport
conda env create -f environment.yml
conda activate mdt_env
chmod +x mdt.py
```

S3.2 Build From Sources

MDTransport is available on GitHub page and can be install using pip

```
pip install git+https://github.com/ShahResearchGroup/MDTransport.git
or
git clone https://github.com/ShahResearchGroup/MDTransport.git
cd MDTransport
pip install -e .
```

S3.3 Installation via Package Managers

MDTransport is available as a pre-compiled package for Linux, macOS, and Windows. It can be installed through the Python Package Index (PyPI) or the Conda-forge channel.

To install the package via PyPI, use:

```
pip install MDTransport
```

Alternatively, MDTransport can be installed via Conda-forge:

```
conda install -c conda-forge mdtransport
```

S4 Simulation Details

Molecular dynamics (MD) simulations were performed using the GROMACS 2018 software package.[1] The systems were modeled using OPLS-based potential.[2–4] Molecules were packed in a cubic box using the Packmol Python script.[5] Periodic boundary conditions were applied in the x, y, and z directions for all simulations. Initially, an energy minimization using the steepest descent algorithm for 50000 steps was performed to relax the structure. This was followed by an annealing process over 1.5 ns, during which the reference temperature was systematically adjusted. Equilibration run was performed over 25 ns, beginning with a 5 ns NVT equilibration at 298 K using Berendsen thermostat[6] followed by a 20 ns of NPT run for 20 ns (Nosé-Hoover thermostat[7] and Parrinello-Rahman barostat[8] with time constant of 2 and 10 ps, respectively). Finally, a 40 ns of NPT production run was performed for properties calculations. A cut-off of 13.0 Å was applied to both LJ and electrostatic interactions, and long-range LJ interactions were accounted for using dispersion correction terms. Electrostatic interactions were divided into short-range and long-range contributions. Short-range electrostatic interactions were computed through pairwise summation, while long-range electrostatic interactions were treated using the Particle-Mesh Ewald (PME) method.[9] The equations of motion were integrated using the leap-frog algorithm with a 1 fs time step.

Covalent bonds involving hydrogen atoms were constrained using the LINCS algorithm.

S5 Validation

Table S1: Comparison of Diffusion Coefficients ($D \times 10^{-9}$ m^2/s) of Molecular Solvents.

System	MDTransport	MDAnalysis	GROMACS
TIP3P Water	5.31	5.23	5.28
Ethylene glycol	0.33	0.33	0.33
2,2,2-Trifluoroethanol	0.54	0.54	0.54
Acetonitrile	4.79	4.77	4.80
Glycerol	0.02	0.02	0.02

Table S2: Comparison of Diffusion Coefficients ($D \times 10^{-11}$ m^2/s) for Imidazolium-based ILs

System	MDTransport		MDAnalysis		GROMACS	
	Cat.	An.	Cat.	An.	Cat.	An.
[C_2mim][BF_4]	5.26	3.59	5.26	3.59	5.26	3.59
[C_2mim][DCA]	3.79	4.50	3.70	4.51	3.79	4.50
[C_2mim][NO_3]	1.97	1.56	1.97	1.56	1.97	1.56
[C_2mim][NTf_2]	4.53	3.43	4.73	3.42	4.53	3.42
[C_2mim][PF_6]	0.86	0.45	0.86	0.45	0.86	0.45
[C_2mim][TfO]	4.93	2.85	4.52	2.85	4.93	2.85

Table S3: Comparison of Diffusion Coefficients ($D \times 10^{-11}$ m^2/s) for Ammonium-based ILs

System	MDTransport		MDAnalysis		GROMACS	
	Cat.	An.	Cat.	An.	Cat.	An.
[EA][BF_4]	1.24	2.57	1.24	2.57	1.24	2.57
[EA][DCA]	0.53	0.82	0.53	0.82	0.53	0.82
[EA][NO_3]	0.58	1.22	0.58	1.22	0.58	1.22
[EA][NTf_2]	0.60	0.69	0.60	0.69	0.60	0.70
[EA][PF_6]	0.31	0.53	0.31	0.53	0.31	0.53
[EA][TfO]	0.85	0.93	0.85	0.93	0.85	0.93

Table S4: Comparison of Diffusion Coefficients ($D \times 10^{-11}$ m^2/s) for Pyrrolidinium-based ILs

**System**	**MDTransport**		**MDAnalysis**		**GROMACS**	
	**Cat.**	**An.**	**Cat.**	**An.**	**Cat.**	**An.**
[C_2mpyrr][BF_4]	0.33	0.39	0.33	0.39	0.33	0.39
[C_2mpyrr][DCA]	0.52	1.05	0.52	1.05	0.52	1.05
[C_2mpyrr][NTf_2]	0.94	1.03	0.94	1.03	0.94	1.03
[C_2mpyrr][PF_6]	0.05	0.06	0.05	0.07	0.05	0.06
[C_2mpyrr][TfO]	0.67	0.56	0.67	0.56	0.67	0.56

S6 Properties

S6.1 Mean Square Displacement

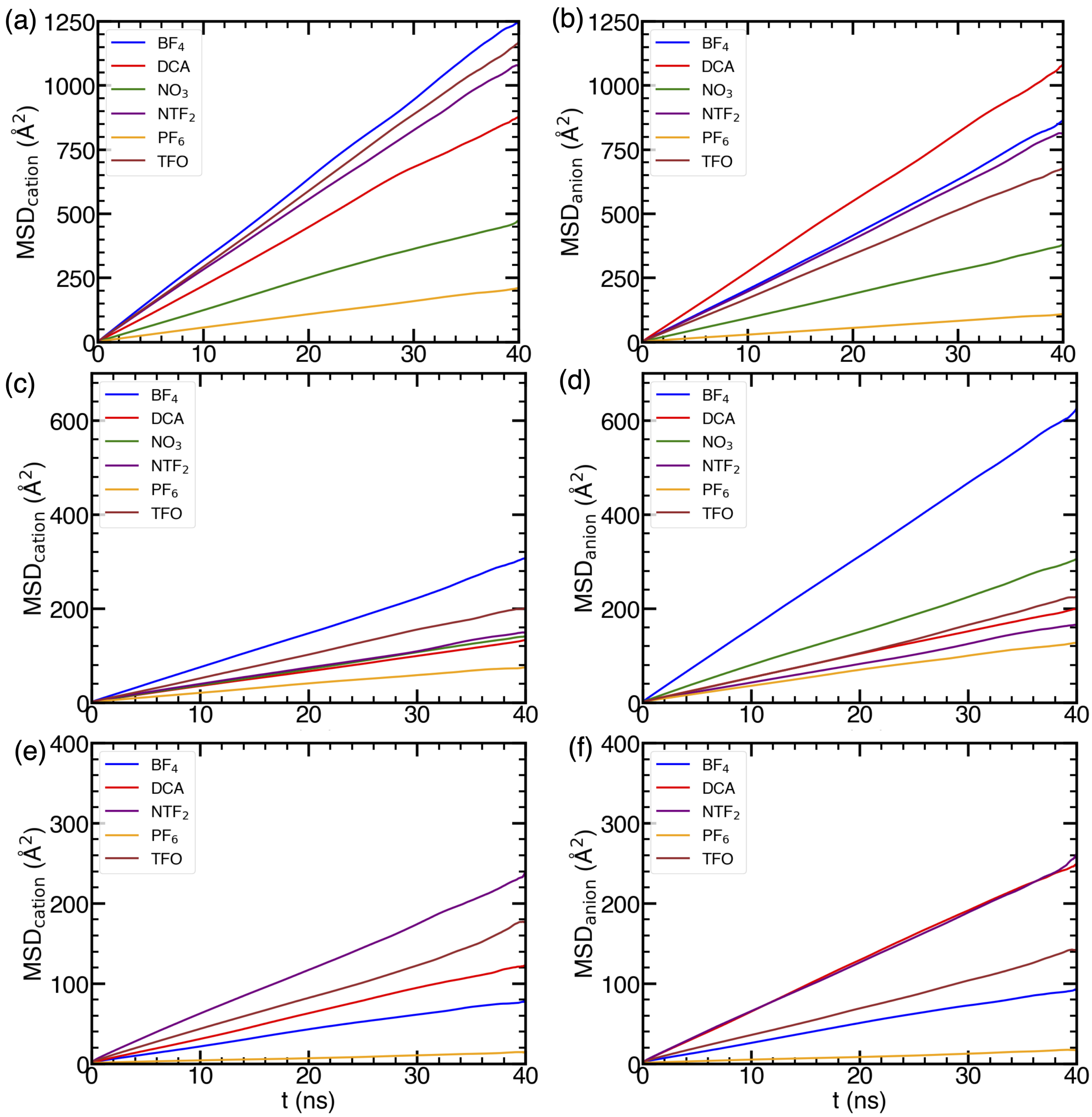

Figure S1: Mean square displacements of ions in (a-b) $[C_2mim]^+$-based ILs, (c-d) $[EA]^+$-based ILs, and (e-f) $[C_2C_1pyrr]^+$-based ILs.

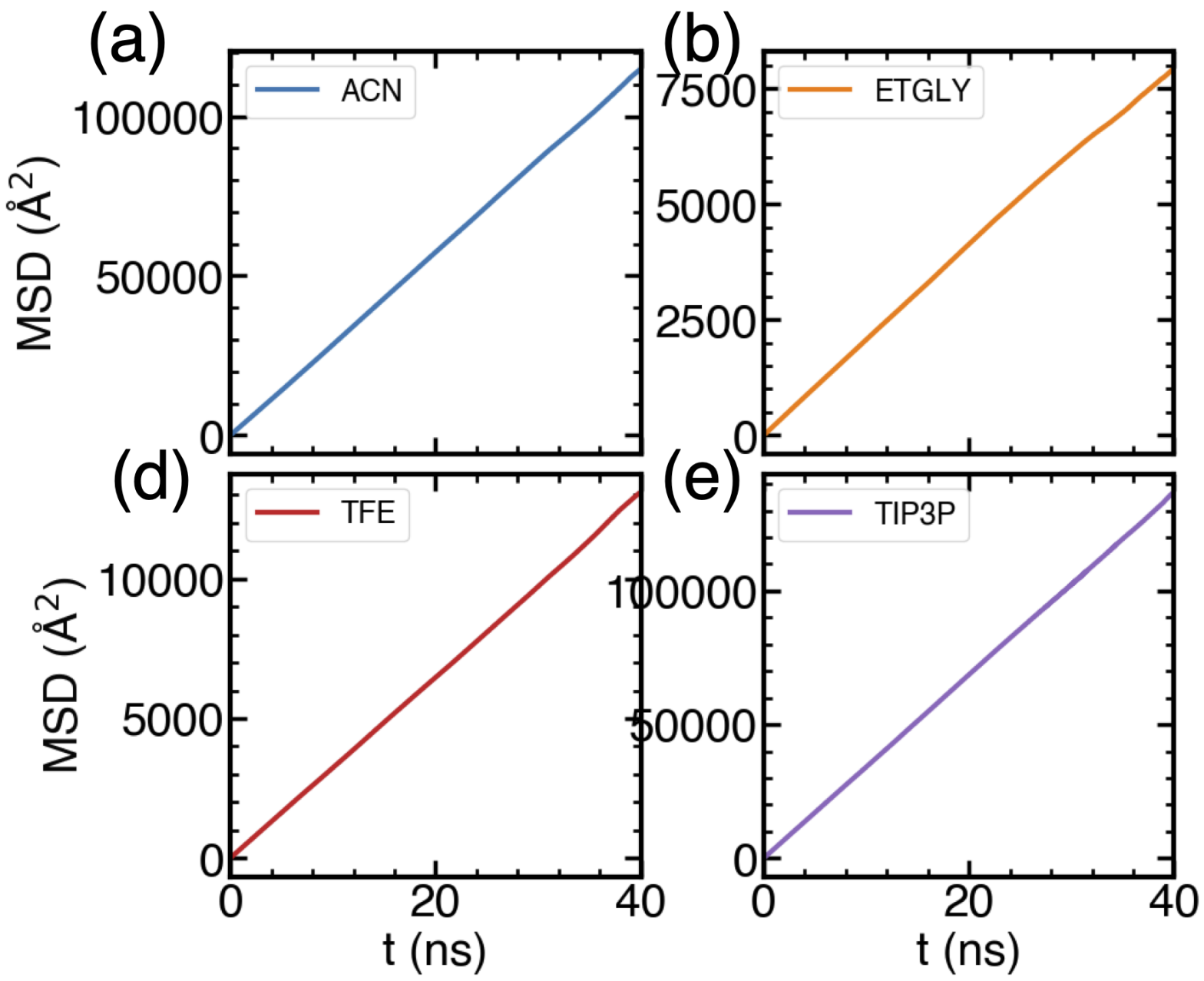

Figure S2: Mean square displacements of (a) Acetonitrile, (b) Ethylene glycol, (c) Glycerol, (d) 2,2,2-Trifluoroethanol, and (e) TIP3P wtaer model.

S6.2 Log MSD vs Log t curves

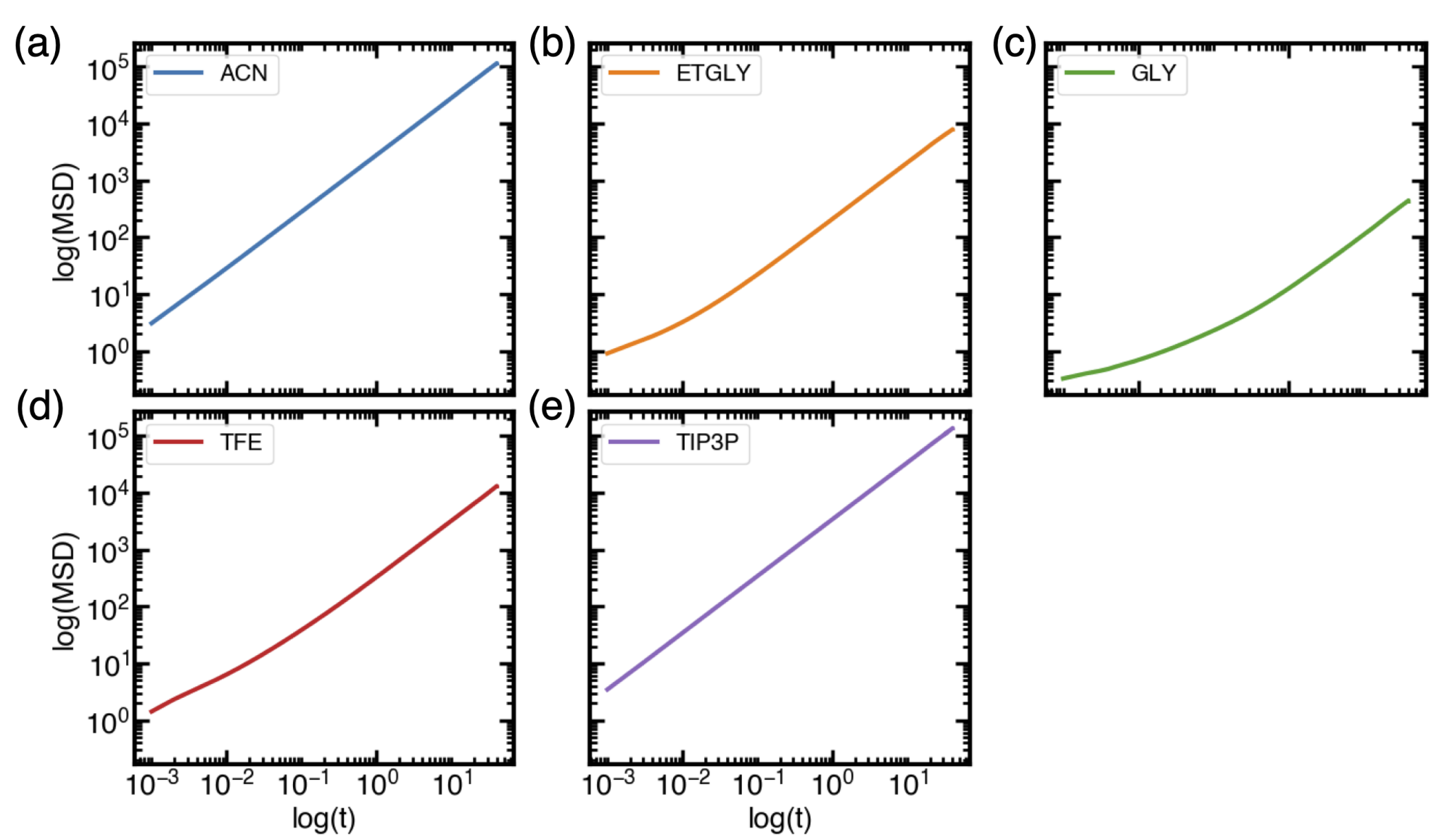

Figure S3: Log MSD vs Log t curves for (a) Acetonitrile, (b) Ethylene glycol, (c) Glycerol, (d) 2,2,2-Trifluoroethanol, and (e) TIP3P water model.

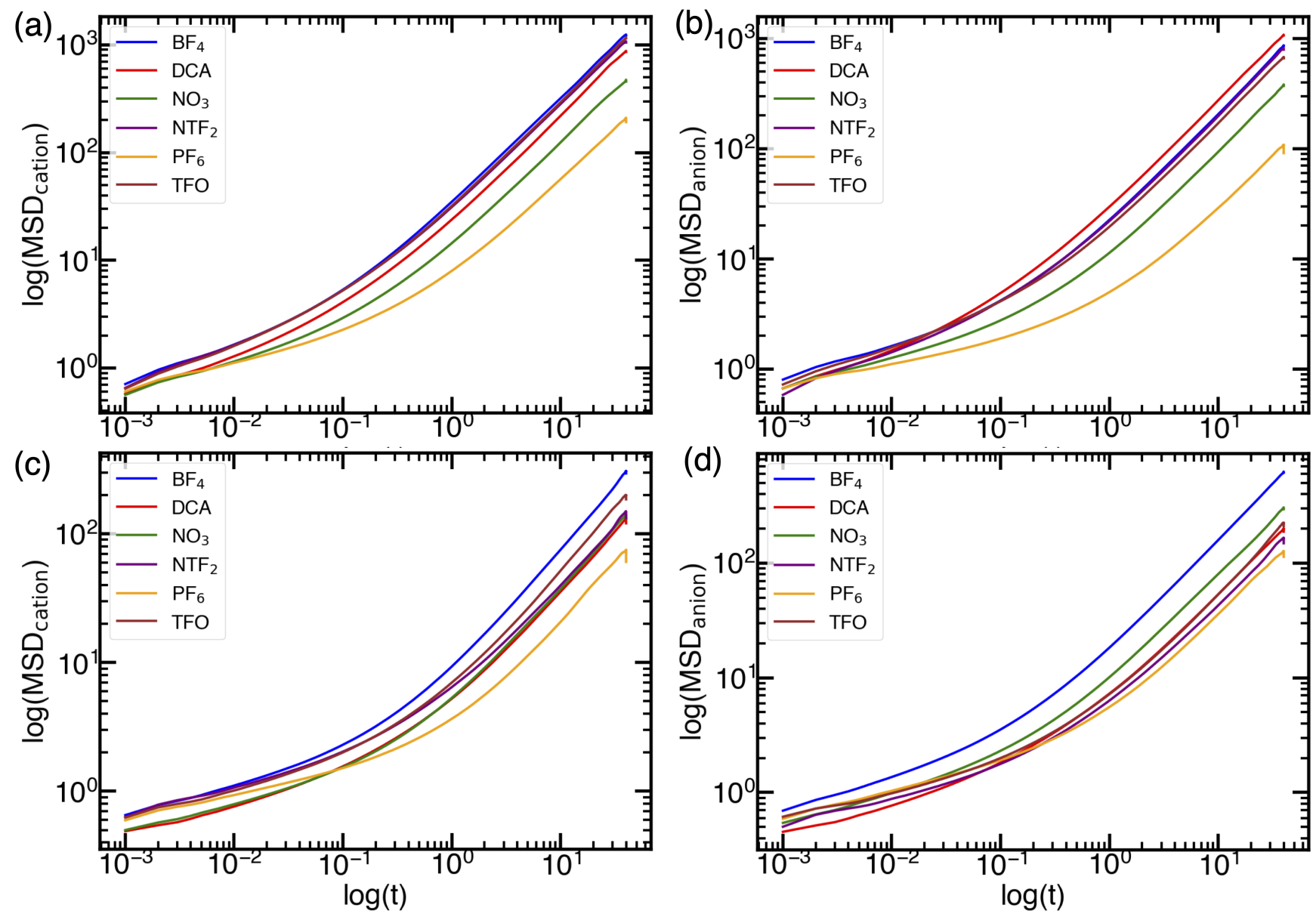

Figure S4: Log MSD vs Log t curves for (a) $[C_2mim]^+$-based ILs, and (b) $[EA]^+$-based ILs.

S6.3 Self-Diffusion Coefficients Calculations

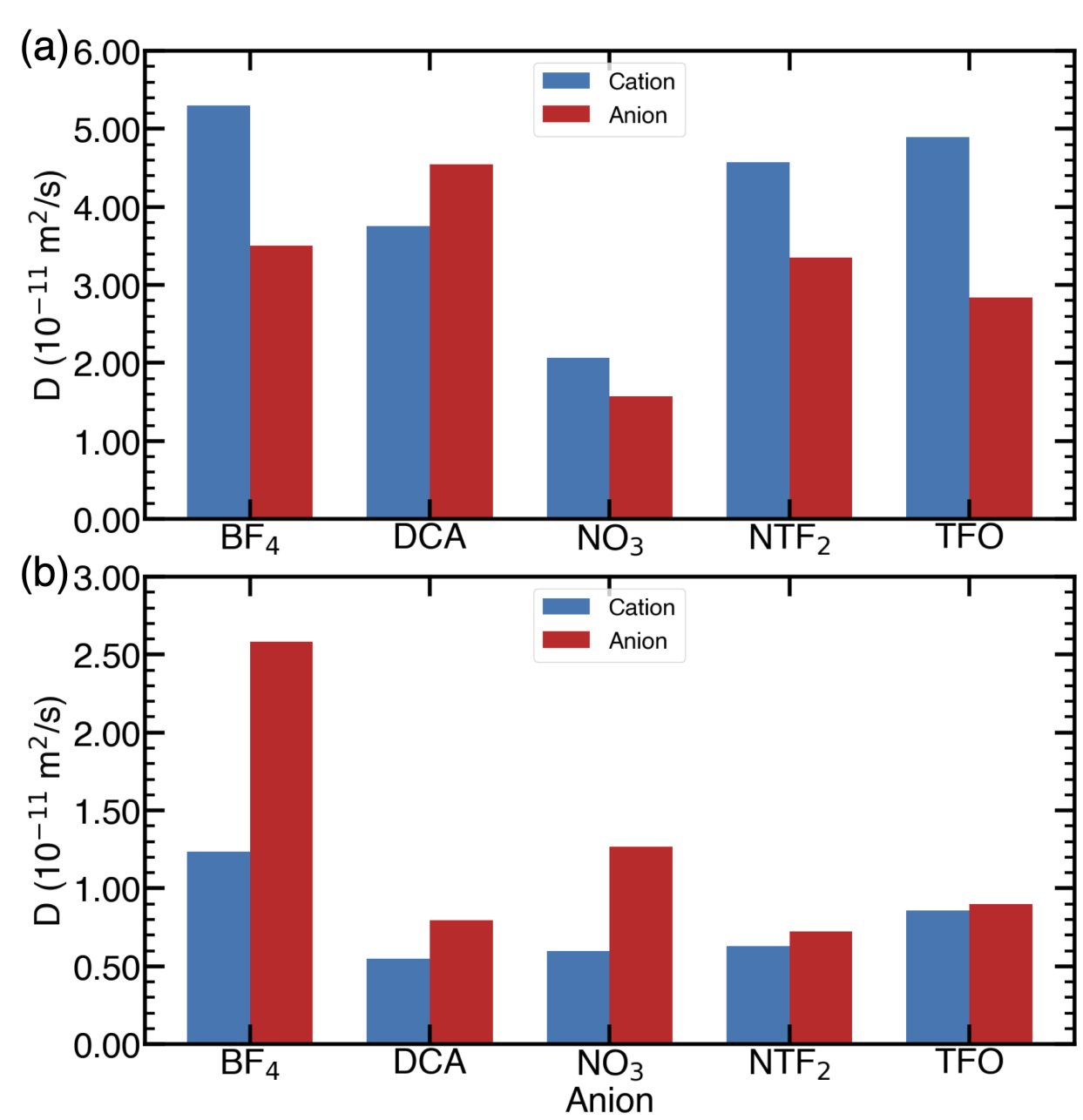

Figure S5: Self-diffusion coefficients of ions in (a) $[C_2mim]^+$-based ILs, and (b) $[EA]^+$-based ILs.

S6.4 Collective Mean Square Displacement

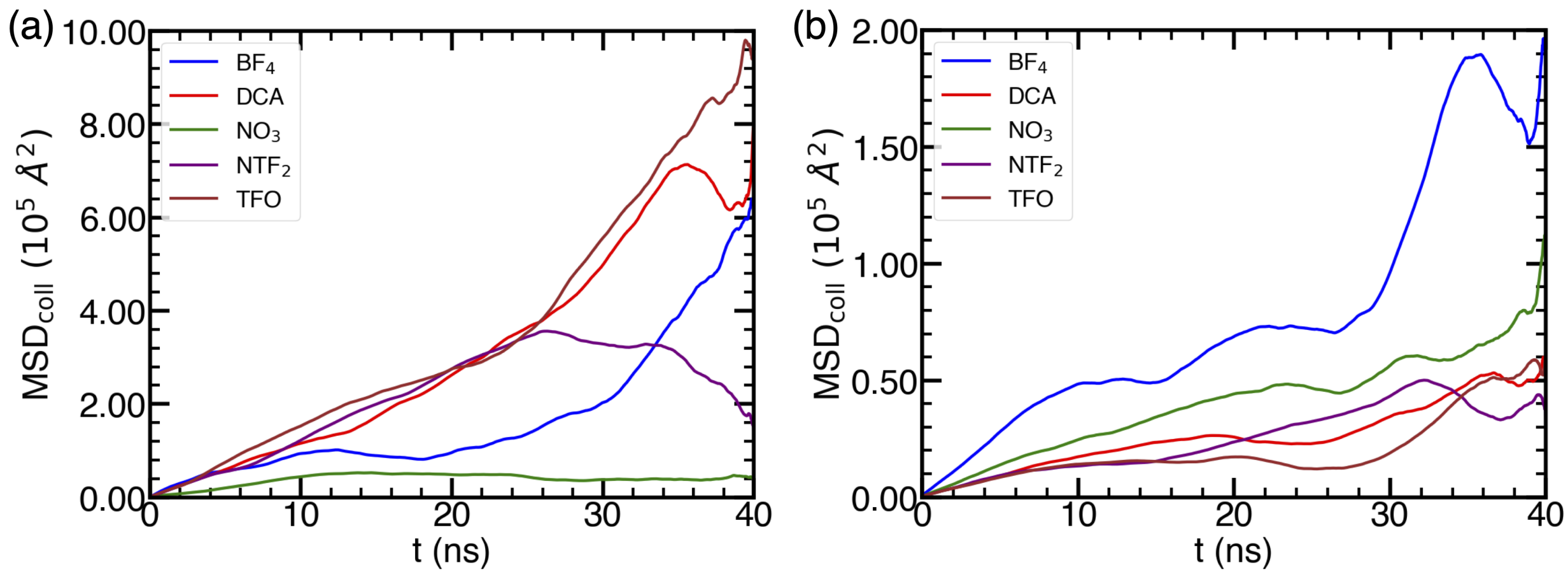

Figure S6: Collective mean square displacement of (a) $[C_2mim]^+$-based ILs, and (b) $[EA]^+$-based ILs.

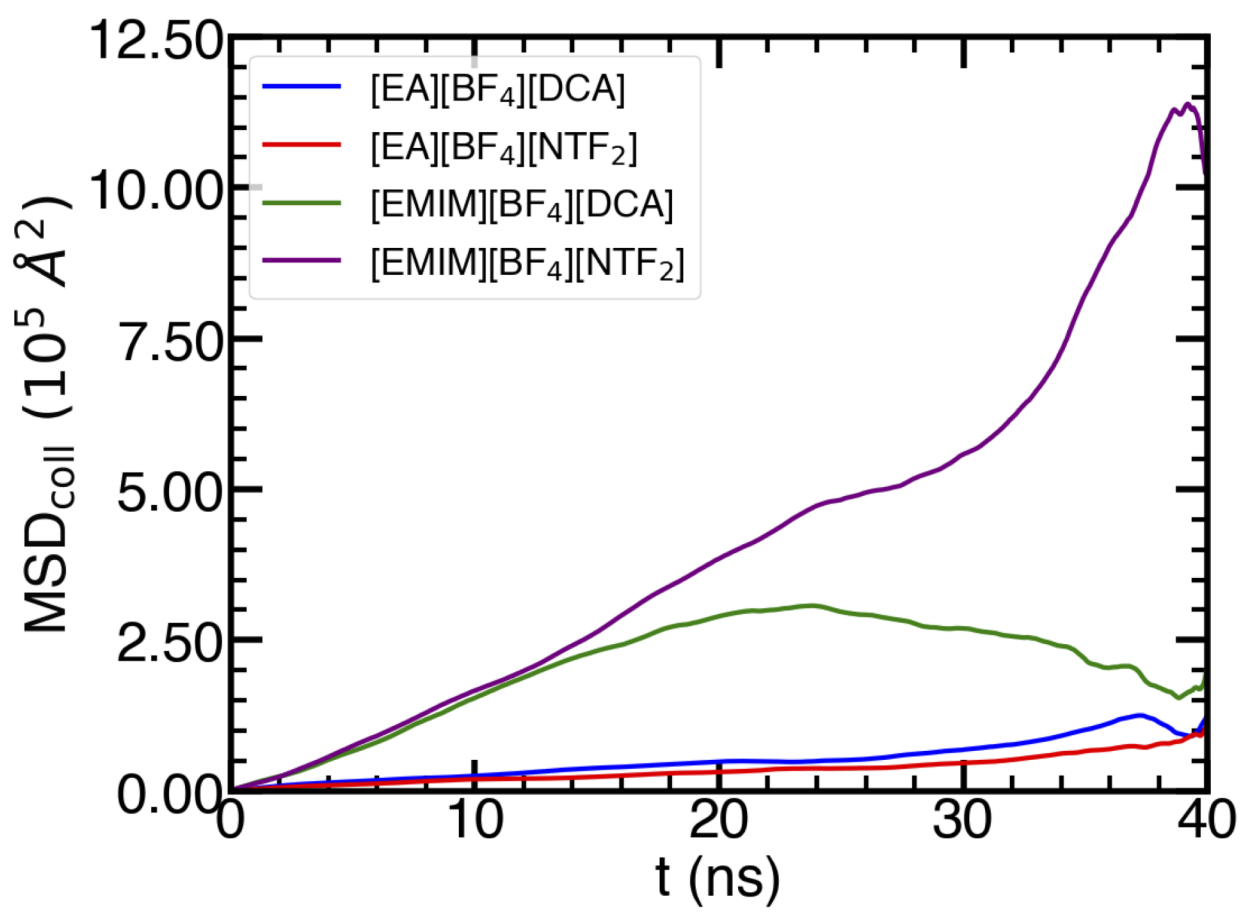

Figure S7: Collective mean square displacement of 50:50 reciprocal mixture of ILs.

S6.5 Ionic Conductivity Calculations

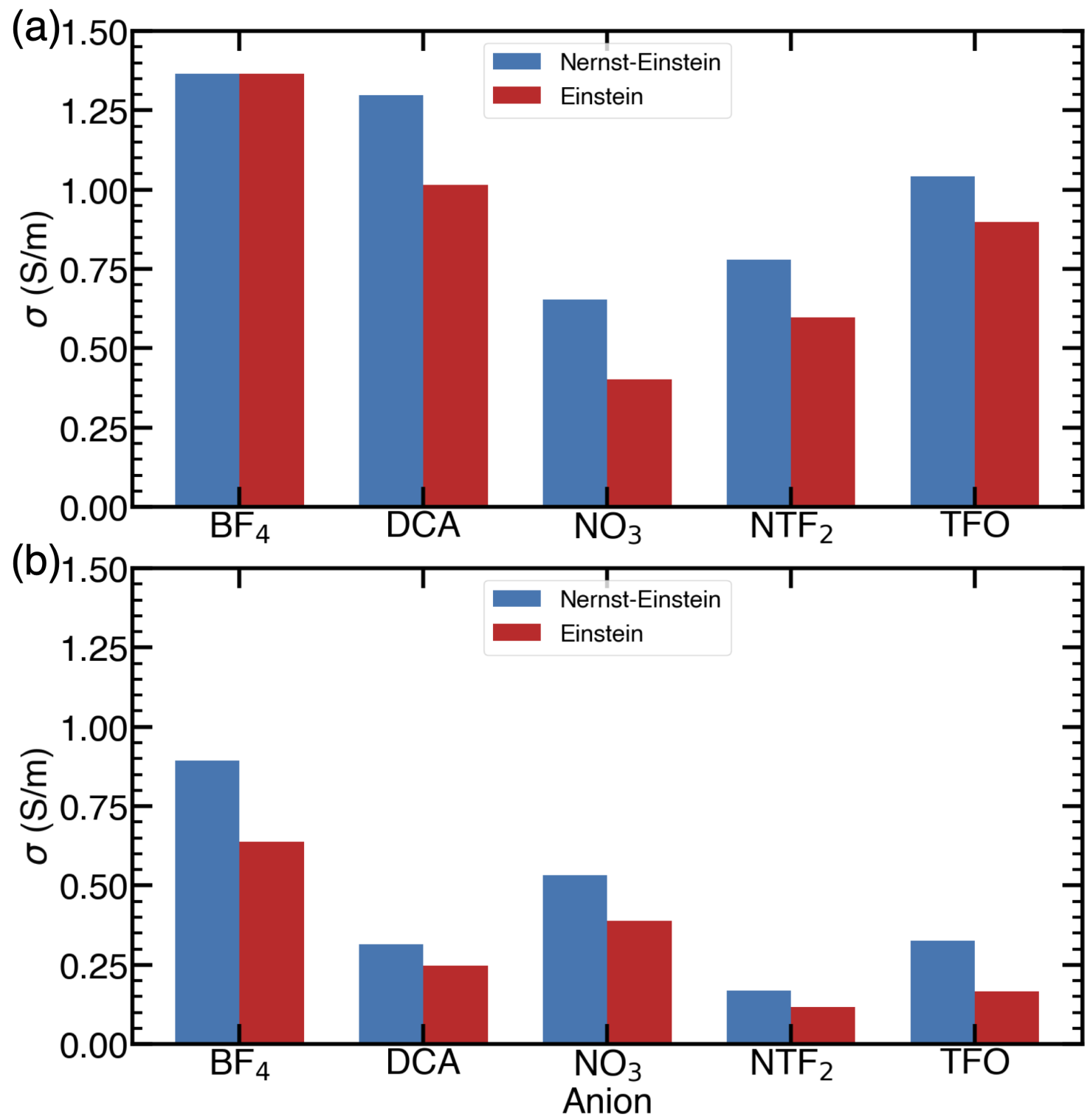

Figure S8: Ionic conductivity of (a) $[C_2mim]^+$-based ILs, and (b) $[EA]^+$-based ILs.

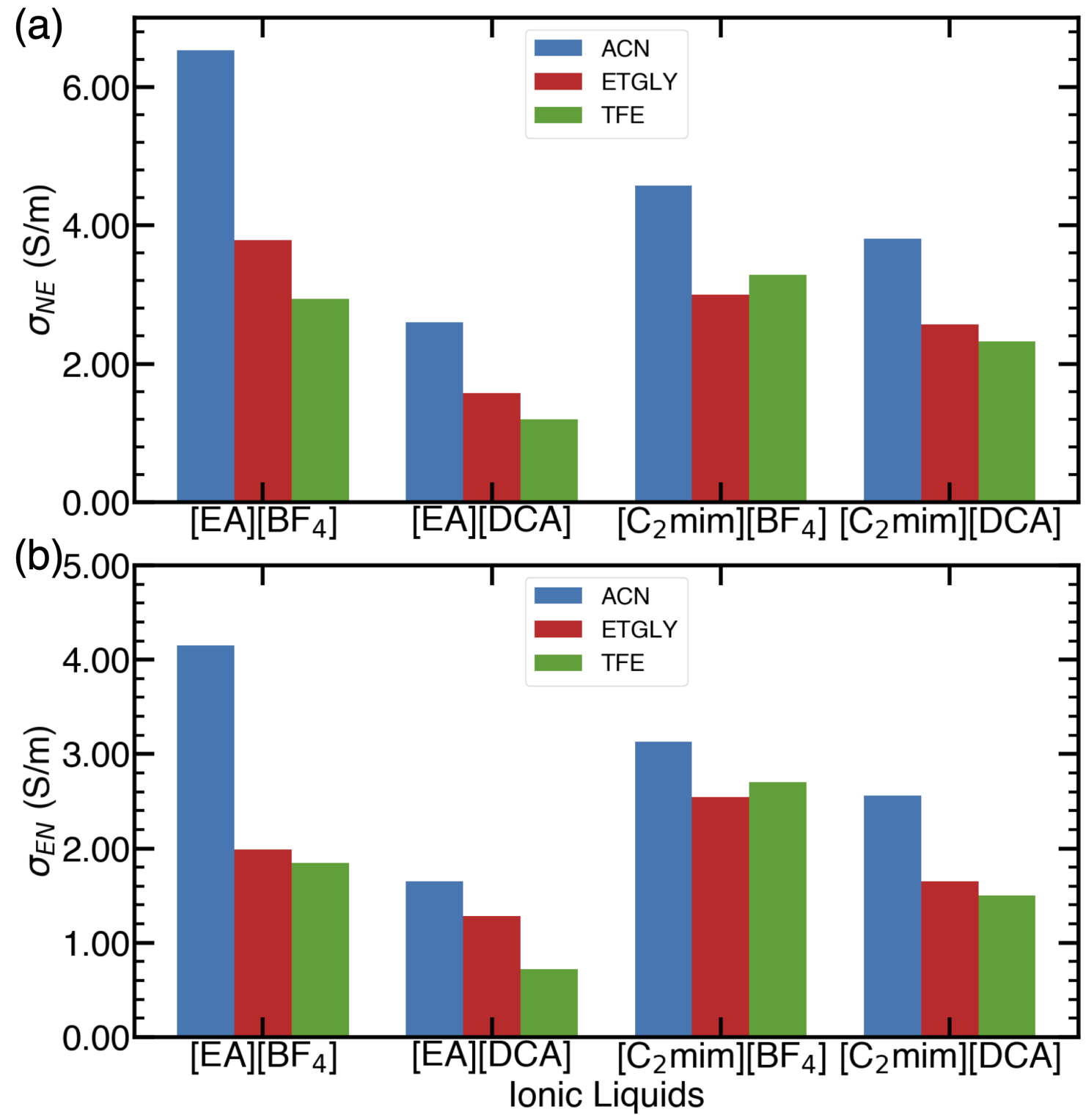

Figure S9: Ionic conductivity of mixture of 50:50 solvents and ionic liquids ([EA][BF_4], [EA][DCA], [C_2mim][BF_4], and [C_2mim][DCA]).

S6.6 Maxwell–Stefan Diffusion and Ionic Conductivity

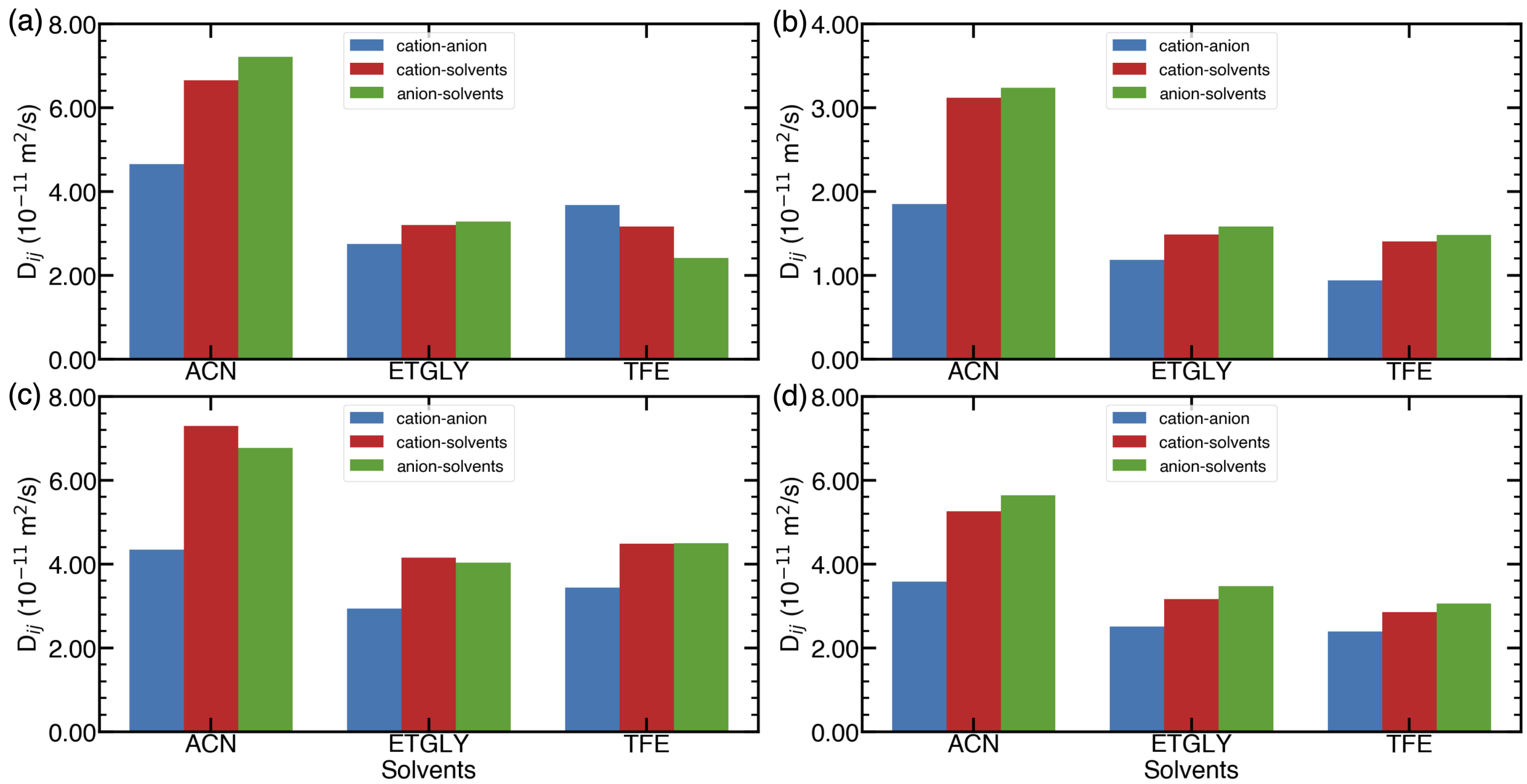

Figure S10: Maxwell–Stefan diffusivity in mixture of 50:50 solvents and (a) [EA][BF_4], (b) [EA][DCA], (c) [C_2mim][BF_4], and (d) [C_2mim][DCA].

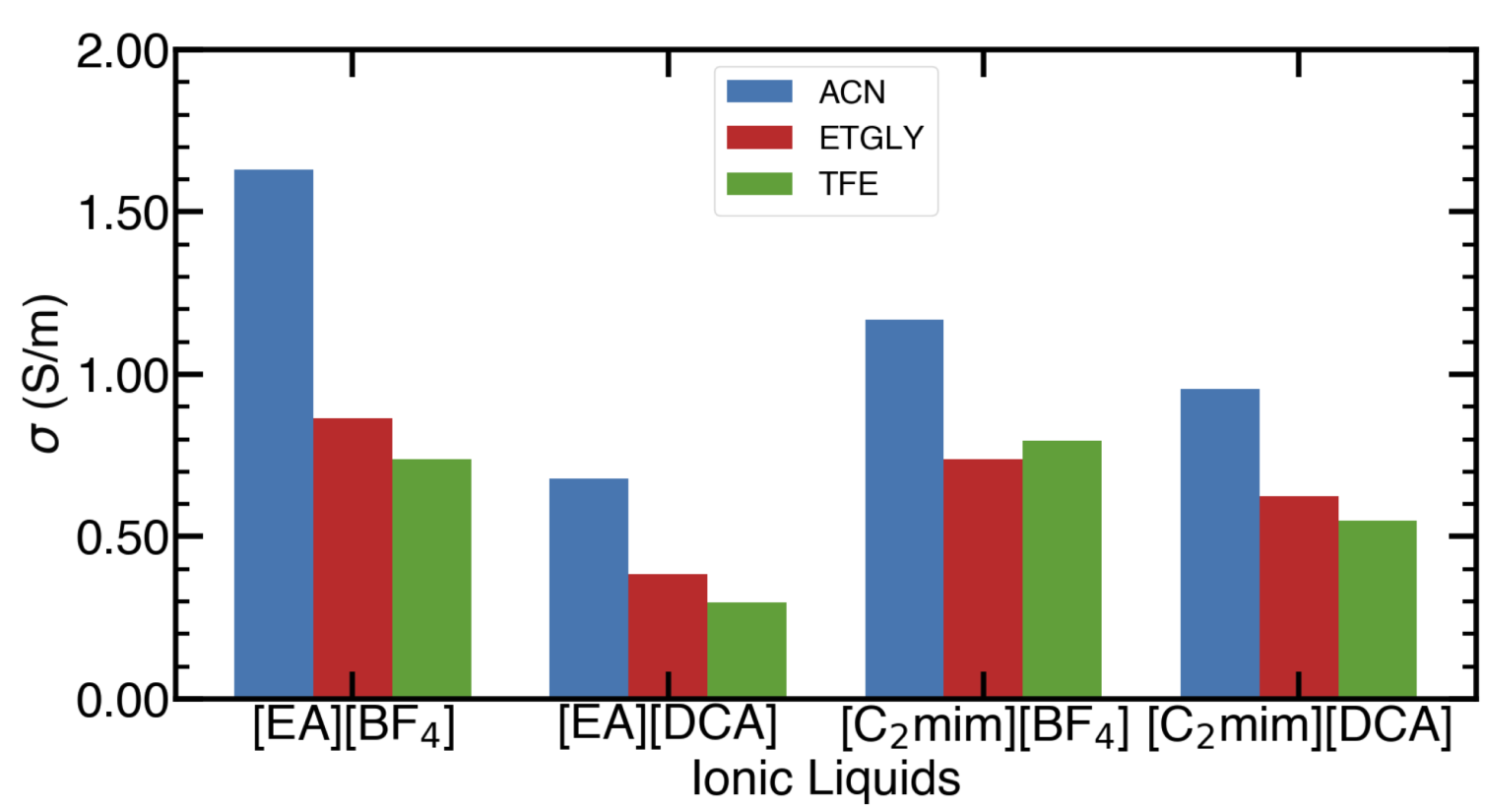

Figure S11: Ionic conductivity of mixture of 50:50 solvents and ionic liquids ([EA][BF_4], [EA][DCA], [C_2mim][BF_4], and [C_2mim][DCA]).

S6.7 Cluster, cage and Ion-pairs Lifetime

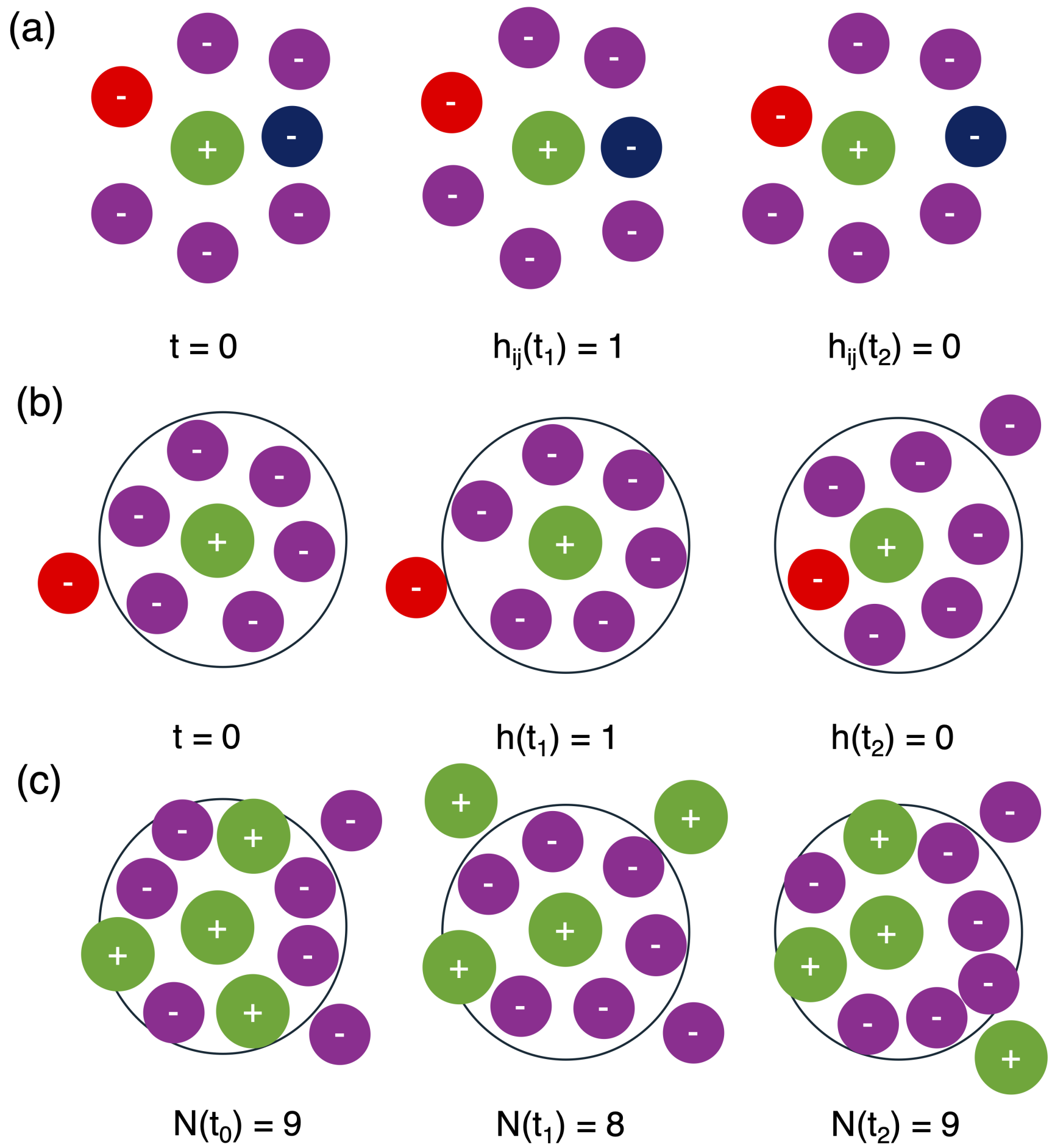

Figure S12: (a) Schematic illustrating the definition of an IP. At a specific time t_0, an IP is determined as the nearest anion (blue) to a central cation (green), or vice versa. This IP is considered intact ($\mathrm{h}_{ij}(\mathrm{t}_1) = 1$) at a later time t_1 until, at time t_2, another counter-ion (red) becomes closer to the central ion, resulting in $\mathrm{h}_{ij}(\mathrm{t}_2) = 0$. (b) A cage is identified by the set of counter-ions surrounding a reference ion. when an ion (red) enters or leaves the cage (blue), $\mathrm{h}(\mathrm{t}_2) = 0$. (c) A cluster is defined as a group of both cations (green) and anions (purple) located within a distance r_c from the reference ion.

References

(1) Abraham, M. J.; Murtola, T.; Schulz, R.; Páll, S.; Smith, J. C.; Hess, B.; Lindahl, E. GROMACS: High performance molecular simulations through multi-level parallelism from laptops to supercomputers. *SoftwareX* **2015**, *1*, 19–25.

(2) Doherty, B.; Acevedo, O. OPLS force field for choline chloride-based deep eutectic solvents. *The Journal of Physical Chemistry B* **2018**, *122*, 9982–9993.

(3) Zhong, X.; Velez, C.; Acevedo, O. Partial charges optimized by genetic algorithms for deep eutectic solvent simulations. *Journal of chemical theory and computation* **2021**, *17*, 3078–3087.

(4) Doherty, B.; Zhong, X.; Acevedo, O. Virtual site OPLS force field for imidazolium-based ionic liquids. *The Journal of Physical Chemistry B* **2018**, *122*, 2962–2974.

(5) Martınez, L.; Andrade, R.; Birgin, E.; Martınez, J. A package for building initial configurations for molecular dynamics simulations. *J. Phys. Chem. Lett* **2009**, *30*, 92157–2164.

(6) Berendsen, H. J.; Postma, J. v.; Van Gunsteren, W. F.; DiNola, A.; Haak, J. R. Molecular dynamics with coupling to an external bath. *The Journal of chemical physics* **1984**, *81*, 3684–3690.

(7) Evans, D. J.; Holian, B. L. The nose–hoover thermostat. *The Journal of chemical physics* **1985**, *83*, 4069–4074.

(8) Parrinello, M.; Rahman, A. Polymorphic transitions in single crystals: A new molecular dynamics method. *Journal of Applied physics* **1981**, *52*, 7182–7190.

(9) Darden, T.; York, D.; Pedersen, L. Particle mesh Ewald: An N log (N) method for Ewald sums in large systems. *The Journal of chemical physics* **1993**, *98*, 10089–10092.
