# Supplementary material for: MDTransport: A Modular, Open-Source, Extensible Python Tool for Computing Transport Properties from GROMACS and LAMMPS Simulations
Source: J Chem Inf Model. 2026 Aug 1;66(16):9989–10003. doi: 10.1021/acs.jcim.6c00558 (PMC13508777; doi:10.1021/acs.jcim.6c00558)
Supplement: Supplementary file 2 [file ci6c00558_si_001.zip › Sample/MSD_Example.pdf]

MDTransport Example

MDTransport: A Python Tool for Computing Transport Properties from GROMACS and LAMMPS Simulations

Ashutosh Kumar Verma, Amey S. Thorat, Jindal K. Shah*

School of Chemical Engineering, Oklahoma State University, Stillwater, OK 74078, United States

E-mail: jindal.shah@okstate.edu

Contents

1 Installation Guide **2**

1.1 Create a Virtual Environment and Run MDTransport Directly 3

1.2 Build From Sources 3

1.3 Install Pre-compiled packages 3

2 Steps to Run MDTransport **4**

2.1 Post-processing GROMACS Files 5

2.1.1 Using `--pdb` 5

2.1.2 Using `--exe` 9

2.1.3 Using `--mda` 12

2.1.4 Using `--chem` 16

2.1.5 Using `--ovito` 20

2.2 Post-processing LAMMPS Files 24

2.2.1 Using `--traj` 24

2.2.2 Using `--com` 28

2.2.3 Using `--mda` 32

2.2.4 Using `--chem` 36

2.2.5 Using `--ovito` 40

1 Installation Guide

The following dependencies are required:

```
python >=3.9
matplotlib >=3.6
pandas >=2.0
numpy >=1.20
scipy >=1.10
tqdm >=4.60
```

MDAnalysis Requirements

```
MDAnalysis is required only if you intend to use the --mda option.
Installation options:
# Recommended if using Conda
conda install -c conda-forge mdanalysis
# Alternative using pip
pip install MDAnalysis
```

Chemfiles Requirements

```
Chemfiles is required only if you intend to use the --chem option.
Installation options:
# Recommended if using Conda (Anaconda/Miniconda)
conda install -c conda-forge chemfiles
# Alternative using pip
pip install chemfiles
```

Ovito Requirements

```
Ovito is required only if you intend to use the --ovito option.
Installation options:
# Recommended if using Conda (Anaconda/Miniconda)
conda install -c conda-forge ovito
# Alternative using pip
pip install ovito
```

1.1 Create a Virtual Environment and Run MDTransport Directly

```
git clone https://github.com/ShahResearchGroup/MDTransport.git
cd MDTransport
conda env create -f environment.yml
conda activate mdt_env
chmod +x MDTransport.py
```

1.2 Build From Sources

MDTransport is available on GitHub page and can be install using pip

```
pip install git+https://github.com/ShahResearchGroup/MDTransport.git
or
git clone https://github.com/ShahResearchGroup/MDTransport.git
cd MDTransport
pip install .
```

1.3 Install Pre-compiled packages

MDTransport is available as pre-compiled packages for the three main operating systems (Linux, MacOS, Windows) on PyPI. You can install these pre-compiled packages using pip

```
pip install MDTransport
```

2 Steps to Run MDTransport

The GROMACS and LAMMPS simulations files are organized under the directory MDTransport_Example. Trajectory and topology files from each MD simulation packages are stored in specific subdirectories. The directory structure used in this work is shown in the Figure 1.

(a)
```
Input_files
└── GROMACS
    ├── init_config
    │   ├── EMIM.pdb
    │   ├── init_config.inp
    │   ├── init_config.pdb
    │   └── NTF2.pdb
    ├── itp_files
    │   ├── atomtypes.itp
    │   ├── EMIM_atomtypes.itp
    │   ├── EMIM.itp
    │   ├── itp_files.itp
    │   ├── NTF2_atomtypes.itp
    │   └── NTF2.itp
    └── sim
        ├── annealing.mdp
        ├── data.top
        ├── init_config.inp
        ├── init_config.pdb
        ├── minimize.mdp
        ├── nvt_eq.mdp
        └── nvt_prod.mdp
```

(b)
```
Input_files
└── LAMMPS
    ├── data.lmp
    ├── EMIM_pack.xyz
    ├── emim.ff
    ├── EMIM.xyz
    ├── lammps.sbatch
    ├── mypack.inp
    ├── NTF2_pack.xyz
    ├── ntf2.ff
    ├── NTF2.xyz
    ├── nvt.lmp
    ├── pair.lmp
    └── simbox.xyz
```

(c)
```
Trajectories
├── GROMACS
│   ├── itp_files
│   │   ├── atomtypes.itp
│   │   ├── EMIM_atomtypes.itp
│   │   ├── EMIM.itp
│   │   ├── itp_files.itp
│   │   ├── NTF2_atomtypes.itp
│   │   └── NTF2.itp
│   ├── nvt_prod.gro
│   ├── nvt_prod.tpr
│   ├── nvt_prod.xtc
│   └── traj.pdb
└── LAMMPS
    ├── com.txt
    ├── data.lmp
    ├── traj.lammpstrj
    └── traj.xtc
```

Figure 1: File organization used for tutorial illustration and post-processing the simulations files (a) GROMACS input files, (b) LAMMPS input files, and (c) GROMACS and LAMPPS output files.

This tutorial illustrates how to use `MDTransport` to calculate mean square displacement and diffusion coefficients, and how to post-process molecular dynamics trajectories generated with both GROMACS and LAMMPS. A system consisting 64 ion-pairs of [C_2mim][NTF_2] is used for this example.

2.1 Post-processing GROMACS Files

2.1.1 Using `--pdb`

It requires a `.pdb` file and a directory containing GROMACS itp files as shown in Figure 1.

1. Navigate to `Sample/Trajectories/GROMACS` in a terminal.
2. Type `python MDTransport.py` and follow screen instruction for the post-processing as shown below or type full command e.g., For MSD calculations: `python MDTransport.py --gro --msd --pdb traj.pdb --itp itp_files`.

```
Last login: Wed Feb 11 09:50:42 on ttys000
asverma@Ashutoshs-iMac ~ % cd /Users/asverma/Downloads/Sample/Trajectories/GROMACS/
asverma@Ashutoshs-iMac GROMACS % python MDTransport.py
================================================================================
MDTransport: GROMACS & LAMMPS Transport Analysis Tools
Copyright (C) 2026  Ashutosh Kumar Verma, Amey Thorat and Jindal Shah

Oklahoma State University, Stillwater, OK 74078.
This is free software, and you are welcome to redistribute it
under certain conditions. See the GNU GPL for details.
================================================================================
=======================================================
          MDTransport: Validating Inputs
=======================================================

Select analysis mode:
 1. GROMACS
 2. LAMMPS

Enter number: 1

Select GROMACS input method:
 1. PDB file
 2. gmx trjconv
 3. MDAnalysis
 4. Chemfiles
 5. OVITO

Enter number: 1
Enter pdb file name or full path (e.g., traj.pdb): traj.pdb
Enter folder name or full path containg itp files (e.g., itp_files): itp_files

Select one or more analysis tasks:
 1. --ONSG     Onsager Transport Coefficients Calculation
 2. --MSD      Mean Square Displacement & Self-diffusion Coefficients Calculation
 3. --NE       Nernst-Einstein Conductivity Calculation
 4. --SPDA     Spatial Decomposition Analysis of Cross-Correlations
 5. --TRA      Einstein, Self- & Cross-correlation Terms Calculation
 6. --EN       Einstein Conductivity Calculation
 7. --IPL      Ion-Pair Lifetime Calculation
 8. --CAGE     Cage Correlation Lifetime Calculation
 9. --CAGE_P   Cage Population (Cluster) Analysis
10. --RDF      Radial Distribution Function Calculation & Analysis
11. --SMD      Stefan-Maxwell Diffusivity and Conductivity Calculation
12. --VEH      Vehicular Transport Mechanism Analysis

Enter selection(s) (e.g., '1 4', '1-3,5', or '--msd --rdf').
(Enter 'all' to select all tasks)
>> 2
=====================================================================================
SYSTEM TOPOLOGY
=====================================================================================
Resname  | Type   | Name (s)        | Mass       | Charge     | Selection Example
-------------------------------------------------------------------------------------
EMI      | CA     | C06             | 12.011     | 0.114      | resname EMI type CA
EMI      | CM     | C05             | 12.011     | -0.122     | resname EMI type CM
EMI      | CR     | C01             | 12.011     | -0.044     | resname EMI type CR
EMI      | CT     | C0N             | 12.011     | 0.085      | resname EMI type CT
```

```
EMI       | CW     | C03, C04        | 12.011      | -0.079      | resname EMI type CW
EMI       | HA     | H0E, H0F        | 1.011       | -0.001      | resname EMI type HA
EMI       | HM     | H0C, H0B, H0D   | 1.011       | 0.065       | resname EMI type HM
EMI       | HR     | H07             | 1.008       | 0.121       | resname EMI type HR
EMI       | HT     | H0P, H0O, H0Q   | 1.011       | -0.026      | resname EMI type HT
EMI       | HW     | H08, H09        | 1.008       | 0.115       | resname EMI type HW
EMI       | NA     | N02, N00        | 14.007      | 0.189       | resname EMI type NA
NSC       | CBT    | CBT             | 12.011      | 0.268       | resname NSC type CBT
NSC       | F1     | F1              | 18.998      | -0.131      | resname NSC type F1
NSC       | NBT    | NBT             | 14.000      | -0.589      | resname NSC type NBT
NSC       | OBT    | OBT             | 15.999      | -0.512      | resname NSC type OBT
NSC       | SBT    | SBT             | 32.066      | 1.085       | resname NSC type SBT
=====================================================================================
Enter start and end frames in ps (or press Enter to use full trajectory) (e.g., 0 2000):
Enable advanced MSD settings (MDAnalysis or Chemfiles required)? (Y/N): N
>> COM-based MSD calculations only.
Enter resnames to computes MSD, separated by spaces (e.g., EMI BF). Press Enter to continue a
nd process all species:
Enter directions for MSD analysis (or press Enter for all) (e.g., x y z):
Enter resnames to skip, separated by spaces (e.g., SOL GRA). Press Enter to continue and proc
ess all species:


=======================================================
             MDTransport: Input Summary
=======================================================
Analysis Mode                            : GROMACS
GROMACS Mode                             : PDB
PDB                                      : traj.pdb
ITP Folder                               : itp_files
Analysis Task(s)                         : MSD
Number of cores                          : 8
=======================================================

Proceed with these inputs? (Y/n): Y
Continuing with execution...

=======================================================
   MDTransport: All inputs validated successfully
=======================================================


=====================================================================================
                        MDTransport: Starting Data Preparation
                                 2026-02-11 10:02:36
=====================================================================================

Reading PDB: 100%|█████████████████████████████████████████| 172M/172M [00:00<00:00, 429MB/s]
Processing Snapshots: 100%|███████████████████████| 1001/1001 [00:00<00:00, 1044.27 frames/s]


=======================================================
                 System Properties
=======================================================
Average Volume   : 26.077 nm³
Average Density  : 1594.866 kg/m³
EMI              : 64
NSC              : 64
```

```
EMI_charge       : 0.718
NSC_charge       : -0.718
=======================================================



=====================================================================================
                        MDTransport: Data Preparation Finished
                                 2026-02-11 10:02:37
                                Elapsed Time: 0:00:01
=====================================================================================



=====================================================================================
            MDTransport: Starting Self-diffusion Coefficients Calculation
                                 2026-02-11 10:02:37
=====================================================================================

Unwrapping Trajectory: 100%|████████████████████████| 128/128 [00:00<00:00, 8646.80species/s]
Running 3D MSD in (['x', 'y', 'z'])
Calculating MSD: 100%|████████████████████████████████████| 2/2 [00:00<00:00, 16.44species/s]
Finding best time window: 100%|█| 3240/3240 [00:00<00:00, 205752.56window/s, avg|β-1|=0.0019]

Best time window for linear fit: 7120.0 – 15120.0 ps | avg|β-1|: 0.0019

=====================================================================================
Self-Diffusion Coefficients Summary
=====================================================================================
Species  Dimensions  Time Window (ps)  D (10⁻¹¹ m²/s)
-------------------------------------------------------
EMI      3D          7120.0-15120.0    9.555
NSC      3D          7120.0-15120.0    8.052
=====================================================================================



=====================================================================================
            MDTransport: Self-diffusion Coefficients Calculation Finished
                                 2026-02-11 10:02:37
                                Elapsed Time: 0:00:00
                               Total Wall Time: 0:00:01
=====================================================================================

asverma@Ashutoshs-iMac GROMACS %
```

2.1.2 Using `--exe`

The GROMACS need to be installed for this option as `MDTransport` generate a `traj.pdb` file first using GROMACS `gmx trjconv command` (a `.trr` or `.xtc` and `.tpr` files required) and process it same as `--pdb` option does without needing of GROMACS itp files.

1. Navigate to `Sample/Trajectories/GROMACS` in a terminal.
2. Type `python MDTransport.py` and follow screen instruction for the post-processing as shown below or type full command e.g., For MSD calculations: `python MDTransport.py --gro --msd --exe gmx --t_steps 1`.

```
Last login: Mon Feb  2 23:07:42 on ttys001
asverma@Ashutoshs-iMac ~ % cd /Users/asverma/Downloads/MDTransport_Example/GROMACS_EMI_20_BF_20/using_exe_mda_chem
asverma@Ashutoshs-iMac using_exe_mda_chem % python MDTransport.py
================================================================================
MDTransport: GROMACS & LAMMPS Transport Analysis Tools
Copyright (C) 2026  Ashutosh Kumar Verma, Amey Thorat and Jindal Shah

Oklahoma State University, Stillwater, OK 74078.
This is free software, and you are welcome to redistribute it
under certain conditions. See the GNU GPL for details.
================================================================================
=======================================================
          MDTransport: Validating Inputs
=======================================================

Select analysis mode:
 1. GROMACS
 2. LAMMPS

Enter number: 1

Select GROMACS input method:
 1. PDB file
 2. gmx trjconv
 3. MDAnalysis
 4. Chemfiles
 5. OVITO

Enter number: 2
Enter GROMACS executable name (e.g., gmx): gmx

Select one or more analysis tasks:
 1. --ONSG     Onsager Transport Coefficients Calculation
 2. --MSD      Mean Square Displacement & Self-diffusion Coefficients Calculation
 3. --NE       Nernst-Einstein Conductivity Calculation
 4. --SPDA     Spatial Decomposition Analysis of Cross-Correlations
 5. --TRA      Einstein, Self- & Cross-correlation Terms Calculation
 6. --EN       Einstein Conductivity Calculation
 7. --IPL      Ion-Pair Lifetime Calculation
 8. --CAGE     Cage Correlation Lifetime Calculation
 9. --CAGE_P   Cage Population (Cluster) Analysis
10. --RDF      Radial Distribution Function Calculation & Analysis
11. --SMD      Stefan-Maxwell Diffusivity and Conductivity Calculation
12. --VEH      Vehicular Transport Mechanism Analysis

Enter selection(s) (e.g., '1 4', '1-3,5', or '--msd --rdf').
(Enter 'all' to select all tasks)
>> 2
=====================================================================================
SYSTEM TOPOLOGY
=====================================================================================
Resname  | Type   | Name (s)        | Mass       | Charge     | Selection Example
-------------------------------------------------------------------------------------
EMI      | CA     | C06             | 12.011     | 0.096      | resname EMI type CA
EMI      | CM     | C05             | 12.011     | -0.206     | resname EMI type CM
EMI      | CR     | C01             | 12.011     | -0.060     | resname EMI type CR
EMI      | CT     | C0N             | 12.011     | 0.065      | resname EMI type CT
EMI      | CW     | C03, C04        | 12.011     | -0.093     | resname EMI type CW
EMI      | HA     | H0E, H0F        | 1.011      | 0.019      | resname EMI type HA
EMI      | HM     | H0C, H0B, H0D   | 1.011      | 0.096      | resname EMI type HM
EMI      | HR     | H07             | 1.008      | 0.153      | resname EMI type HR
EMI      | HT     | H0Q, H0P, H0O   | 1.011      | -0.019     | resname EMI type HT
EMI      | HW     | H08, H09        | 1.008      | 0.131      | resname EMI type HW
EMI      | NA     | N02, N00        | 14.007     | 0.206      | resname EMI type NA
BF       | B      | B               | 10.811     | 1.007      | resname BF type B
BF       | F      | F               | 18.998     | -0.453     | resname BF type F
=====================================================================================
Enter t_steps for analysis (or press Enter for default 1) (e.g., 1): 1
t_steps = 1 ps
Enter start and end frames in ps (or press Enter to use full trajectory) (e.g., 0 2000):
Enable advanced MSD settings (MDAnalysis or Chemfiles required)? (Y/N): N
>> COM-based MSD calculations only.
Enter directions for MSD analysis (or press Enter for all) (e.g., x y z):
Enter resnames to skip, separated by spaces (e.g., SOL GRA). Press Enter to continue and process all species:
```

```
=======================================================
             MDTransport: Input Summary
=======================================================
Analysis Mode                             : GROMACS
GROMACS Mode                              : EXE
EXE                                       : gmx
topology file                             : /Users/asverma/Downloads/MDTransport_Example/GROMACS_EMI_20_BF_20/using_exe_
mda_chem/nvt_prod.tpr
trajectory file                           : /Users/asverma/Downloads/MDTransport_Example/GROMACS_EMI_20_BF_20/using_exe_
mda_chem/nvt_prod.xtc
t_steps                                   : 1 ps
Analysis Task(s)                          : MSD
Number of cores                           : 8
=======================================================

Proceed with these inputs? (Y/n): Y
Continuing with execution...

=======================================================
   MDTransport: All inputs validated successfully
=======================================================


=============================================================================
                       MDTransport: Starting Data Preparation
                                2026-02-02 23:09:07
=============================================================================

Generating PDB: 20000.0ps [00:01, 12893.83ps/s]
Reading PDB: 100%|██████████████████████████████████████████████████████████████████| 153M/153M [00:00<00:00, 1.44GB/s]
Processing Snapshots: 100%|█████████████████████████████████████████████████| 4001/4001 [00:00<00:00, 4311.78 frames/s]


=======================================================
                 System Properties
=======================================================
Average Volume   : 5.225 nm³
Average Density  : 1258.603 kg/m³
EMI              : 20
BF               : 20
EMI_charge       : 0.805
BF_charge        : -0.805
=======================================================



=============================================================================
                       MDTransport: Data Preparation Finished
                                2026-02-02 23:09:09
                               Elapsed Time: 0:00:02
=============================================================================



=============================================================================
            MDTransport: Starting Self-diffusion Coefficients Calculation
                                2026-02-02 23:09:09
=============================================================================

Unwrapping Trajectory: 100%|████████████████████████████████████████████████████| 40/40 [00:00<00:00, 2939.25species/s]
Running 3D MSD in (['x', 'y', 'z'])
Calculating MSD: 100%|██████████████████████████████████████████████████████████████| 2/2 [00:00<00:00, 19.80species/s]
Finding best time window: 100%|███████████████████████████| 3240/3240 [00:00<00:00, 301755.19window/s, avg|β-1|=0.0696]

Best time window for linear fit: 2005.0 – 10005.0 ps | avg|β-1|: 0.0696

=============================================================================
Self-Diffusion Coefficients Summary
=============================================================================
Species  Dimensions  Time Window (ps)  D (10⁻¹¹ m²/s)
-------------------------------------------------------
EMI      3D          2005.0-10005.0    4.560
BF       3D          2005.0-10005.0    1.324
=============================================================================
```

2.1.3 Using `--mda`

MDAnalysis is required for this options as `MDTransport` uses it for extracting species data from `.trr` or `.xtc` and `.tpr` files and process it without generating intermediate `.pdb` file as it was generated when using `--exe`.

1. Navigate to `Sample/Trajectories/GROMACS` in a terminal.
2. Type `python MDTransport.py` and follow screen instruction for the post-processing as shown below or type full command e.g., For MSD calculations: `python MDTransport.py --gro --msd --mda --t_steps 1`.

```
Last login: Wed Feb 11 10:03:47 on ttys002
asverma@Ashutoshs-iMac ~ % cd /Users/asverma/Downloads/Sample/Trajectories/GROMACS/
asverma@Ashutoshs-iMac GROMACS % python MDTransport.py
================================================================================
MDTransport: GROMACS & LAMMPS Transport Analysis Tools
Copyright (C) 2026  Ashutosh Kumar Verma, Amey Thorat and Jindal Shah

Oklahoma State University, Stillwater, OK 74078.
This is free software, and you are welcome to redistribute it
under certain conditions. See the GNU GPL for details.
================================================================================
=======================================================
          MDTransport: Validating Inputs
=======================================================

Select analysis mode:
 1. GROMACS
 2. LAMMPS

Enter number: 1

Select GROMACS input method:
 1. PDB file
 2. gmx trjconv
 3. MDAnalysis
 4. Chemfiles
 5. OVITO

Enter number: 3

Select one or more analysis tasks:
 1. --ONSG     Onsager Transport Coefficients Calculation
 2. --MSD      Mean Square Displacement & Self-diffusion Coefficients Calculation
 3. --NE       Nernst-Einstein Conductivity Calculation
 4. --SPDA     Spatial Decomposition Analysis of Cross-Correlations
 5. --TRA      Einstein, Self- & Cross-correlation Terms Calculation
 6. --EN       Einstein Conductivity Calculation
 7. --IPL      Ion-Pair Lifetime Calculation
 8. --CAGE     Cage Correlation Lifetime Calculation
 9. --CAGE_P   Cage Population (Cluster) Analysis
10. --RDF      Radial Distribution Function Calculation & Analysis
11. --SMD      Stefan-Maxwell Diffusivity and Conductivity Calculation
12. --VEH      Vehicular Transport Mechanism Analysis

Enter selection(s) (e.g., '1 4', '1-3,5', or '--msd --rdf').
(Enter 'all' to select all tasks)
>> 2

=====================================================================================
SYSTEM TOPOLOGY (GROMACS)
=====================================================================================
Resname    | Type     | Name(s)         | Count    | Mass     | Selection Example
-------------------------------------------------------------------------------------
EMI        | CA       | C06             | 64       | 12.01    | resname EMI type CA
EMI        | CM       | C05             | 64       | 12.01    | resname EMI type CM
EMI        | CR       | C01             | 64       | 12.01    | resname EMI type CR
EMI        | CT       | C0N             | 64       | 12.01    | resname EMI type CT
EMI        | CW       | C03,C04         | 128      | 12.01    | resname EMI type CW
```

```
EMI         | HA        | H0E,H0F         | 128       | 1.01      | resname EMI type HA
EMI         | HM        | H0B,H0C,H0D     | 192       | 1.01      | resname EMI type HM
EMI         | HR        | H07             | 64        | 1.01      | resname EMI type HR
EMI         | HT        | H0O,H0P,H0Q     | 192       | 1.01      | resname EMI type HT
EMI         | HW        | H08,H09         | 128       | 1.01      | resname EMI type HW
EMI         | NA        | N00,N02         | 128       | 14.01     | resname EMI type NA
NSC         | CBT       | CBT             | 128       | 12.01     | resname NSC type CBT
NSC         | F1        | F1              | 384       | 19.00     | resname NSC type F1
NSC         | NBT       | NBT             | 64        | 14.00     | resname NSC type NBT
NSC         | OBT       | OBT             | 256       | 16.00     | resname NSC type OBT
NSC         | SBT       | SBT             | 128       | 32.07     | resname NSC type SBT
=========================================================================================

Enter t_steps for analysis (or press Enter for default 1) (e.g., 1): 1
t_steps = 1 ps
Enter start and end frames in ps (or press Enter to use full trajectory) (e.g., 0 2000):
Enable advanced MSD settings (MDAnalysis or Chemfiles required)? (Y/N): N
>> COM-based MSD calculations only.
Enter resnames to computes MSD, separated by spaces (e.g., EMI BF). Press Enter to continue a
nd process all species:
Enter directions for MSD analysis (or press Enter for all) (e.g., x y z):
Enter resnames to skip, separated by spaces (e.g., SOL GRA). Press Enter to continue and proc
ess all species:


==========================================================
             MDTransport: Input Summary
==========================================================
Analysis Mode                              : GROMACS
GROMACS Mode                               : MDAnalysis
topology file                              : /Users/asverma/Downloads/Sample/Trajectories/GROMA
CS/nvt_prod.tpr
trajectory file                            : /Users/asverma/Downloads/Sample/Trajectories/GROMA
CS/nvt_prod.xtc
t_steps                                    : 1 ps
Analysis Task(s)                           : MSD
Number of cores                            : 8
==========================================================

Proceed with these inputs? (Y/n): Y
Continuing with execution...

==========================================================
   MDTransport: All inputs validated successfully
==========================================================


=========================================================================================
                        MDTransport: Starting Data Preparation
                                 2026-02-11 10:06:18
=========================================================================================

Trajectory does not contain data at the requested interval of 1 ps. Using Trajectory steps of
 20.0 ps.
Processing Snapshots: 100%|██████████████████████████████| 1001/1001 [00:01<00:00, 822.29it/s]


==========================================================
```

```
                 System Properties
=======================================================
Average Volume   : 26.077 nm³
Average Density  : 1594.866 kg/m³
EMI              : 64
NSC              : 64
EMI_charge       : 0.718
NSC_charge       : -0.718
=======================================================



=====================================================================================
                        MDTransport: Data Preparation Finished
                                 2026-02-11 10:06:20
                                Elapsed Time: 0:00:01
=====================================================================================



=====================================================================================
            MDTransport: Starting Self-diffusion Coefficients Calculation
                                 2026-02-11 10:06:20
=====================================================================================

Unwrapping Trajectory: 100%|████████████████████████| 128/128 [00:00<00:00, 8898.61species/s]
Running 3D MSD in (['x', 'y', 'z'])
Calculating MSD: 100%|████████████████████████████████████| 2/2 [00:00<00:00, 17.96species/s]
Finding best time window: 100%|█| 3240/3240 [00:00<00:00, 207705.46window/s, avg|β-1|=0.0019]

Best time window for linear fit: 7120.0 – 15120.0 ps | avg|β-1|: 0.0019

=====================================================================================
Self-Diffusion Coefficients Summary
=====================================================================================
Species  Dimensions  Time Window (ps)  D (10⁻¹¹ m²/s)
-------------------------------------------------------
EMI      3D          7120.0-15120.0    9.555
NSC      3D          7120.0-15120.0    8.052
=====================================================================================



=====================================================================================
            MDTransport: Self-diffusion Coefficients Calculation Finished
                                 2026-02-11 10:06:20
                                Elapsed Time: 0:00:00
                               Total Wall Time: 0:00:01
=====================================================================================

asverma@Ashutoshs-iMac GROMACS %
```

2.1.4 Using --chem

Chemfiles is required for this options and it works similar to MDAnalysis.

1. Navigate to `Sample/Trajectories/GROMACS` in a terminal.

2. Type `python MDTransport.py` and follow screen instruction for the post-processing as shown below or type full command e.g., For MSD calculations: `python MDTransport.py --gro --msd --chem --t_steps 1`.

```
Last login: Wed Feb 11 10:05:33 on ttys002
asverma@Ashutoshs-iMac ~ % cd /Users/asverma/Downloads/Sample/Trajectories/GROMACS/
asverma@Ashutoshs-iMac GROMACS % python MDTransport.py
================================================================================
MDTransport: GROMACS & LAMMPS Transport Analysis Tools
Copyright (C) 2026  Ashutosh Kumar Verma, Amey Thorat and Jindal Shah

Oklahoma State University, Stillwater, OK 74078.
This is free software, and you are welcome to redistribute it
under certain conditions. See the GNU GPL for details.
================================================================================
=======================================================
          MDTransport: Validating Inputs
=======================================================

Select analysis mode:
 1. GROMACS
 2. LAMMPS

Enter number: 1

Select GROMACS input method:
 1. PDB file
 2. gmx trjconv
 3. MDAnalysis
 4. Chemfiles
 5. OVITO

Enter number: 4

Select one or more analysis tasks:
 1. --ONSG     Onsager Transport Coefficients Calculation
 2. --MSD      Mean Square Displacement & Self-diffusion Coefficients Calculation
 3. --NE       Nernst-Einstein Conductivity Calculation
 4. --SPDA     Spatial Decomposition Analysis of Cross-Correlations
 5. --TRA      Einstein, Self- & Cross-correlation Terms Calculation
 6. --EN       Einstein Conductivity Calculation
 7. --IPL      Ion-Pair Lifetime Calculation
 8. --CAGE     Cage Correlation Lifetime Calculation
 9. --CAGE_P   Cage Population (Cluster) Analysis
10. --RDF      Radial Distribution Function Calculation & Analysis
11. --SMD      Stefan-Maxwell Diffusivity and Conductivity Calculation
12. --VEH      Vehicular Transport Mechanism Analysis

Enter selection(s) (e.g., '1 4', '1-3,5', or '--msd --rdf').
(Enter 'all' to select all tasks)
>> 2
=====================================================================================
SYSTEM TOPOLOGY
=====================================================================================
Resname    | Name(s)         | Count    | Mass     | Charge   | Selection Example
-------------------------------------------------------------------------------------
EMI        | C01             | 64       | 12.01    | -0.0445  | resname EMI name C01
EMI        | C03             | 64       | 12.01    | -0.0791  | resname EMI name C03
EMI        | C04             | 64       | 12.01    | -0.0791  | resname EMI name C04
EMI        | C05             | 64       | 12.01    | -0.1217  | resname EMI name C05
EMI        | C06             | 64       | 12.01    | 0.1139   | resname EMI name C06
EMI        | C0N             | 64       | 12.01    | 0.0851   | resname EMI name C0N
EMI        | H07             | 64       | 1.01     | 0.1206   | resname EMI name H07
```

```
EMI         | H08               | 64        | 1.01      | 0.1149    | resname EMI name H08
EMI         | H09               | 64        | 1.01      | 0.1149    | resname EMI name H09
EMI         | H0B               | 64        | 1.01      | 0.0652    | resname EMI name H0B
EMI         | H0C               | 64        | 1.01      | 0.0652    | resname EMI name H0C
EMI         | H0D               | 64        | 1.01      | 0.0652    | resname EMI name H0D
EMI         | H0E               | 64        | 1.01      | -0.0008   | resname EMI name H0E
EMI         | H0F               | 64        | 1.01      | -0.0008   | resname EMI name H0F
EMI         | H0O               | 64        | 1.01      | -0.0261   | resname EMI name H0O
EMI         | H0P               | 64        | 1.01      | -0.0261   | resname EMI name H0P
EMI         | H0Q               | 64        | 1.01      | -0.0261   | resname EMI name H0Q
EMI         | N00               | 64        | 14.01     | 0.1887    | resname EMI name N00
EMI         | N02               | 64        | 14.01     | 0.1887    | resname EMI name N02
NSC         | CBT               | 128       | 12.01     | 0.2680    | resname NSC name CBT
NSC         | F1                | 384       | 19.00     | -0.1308   | resname NSC name F1
NSC         | NBT               | 64        | 14.00     | -0.5888   | resname NSC name NBT
NSC         | OBT               | 256       | 16.00     | -0.5125   | resname NSC name OBT
NSC         | SBT               | 128       | 32.07     | 1.0848    | resname NSC name SBT
=====================================================================================
Enter t_steps for analysis (or press Enter for default 1) (e.g., 1): 1
t_steps = 1 ps
Enter start and end frames in ps (or press Enter to use full trajectory) (e.g., 0 2000):
Enable advanced MSD settings (MDAnalysis or Chemfiles required)? (Y/N): N
>> COM-based MSD calculations only.
Enter resnames to computes MSD, separated by spaces (e.g., EMI BF). Press Enter to continue an
d process all species:
Enter directions for MSD analysis (or press Enter for all) (e.g., x y z):
Enter resnames to skip, separated by spaces (e.g., SOL GRA). Press Enter to continue and proce
ss all species:


=======================================================
             MDTransport: Input Summary
=======================================================
Analysis Mode                           : GROMACS
GROMACS Mode                            : Chemfiles
topology file                           : /Users/asverma/Downloads/Sample/Trajectories/GROMAC
S/nvt_prod.tpr
trajectory file                         : /Users/asverma/Downloads/Sample/Trajectories/GROMAC
S/nvt_prod.xtc
t_steps                                 : 1 ps
Analysis Task(s)                        : MSD
Number of cores                         : 8
=======================================================

Proceed with these inputs? (Y/n): Y
Continuing with execution...

=======================================================
   MDTransport: All inputs validated successfully
=======================================================


=====================================================================================
                        MDTransport: Starting Data Preparation
                                 2026-02-11 10:07:25
=====================================================================================

Trajectory does not contain data at the requested interval of 1 ps. Using Trajectory steps of
20.0 ps.
```

```
Processing Snapshots: 100%|█████████████████████████████| 1001/1001 [00:00<00:00, 1505.98it/s]


=======================================================
                 System Properties
=======================================================
Average Volume   : 26.077 nm³
Average Density  : 1594.866 kg/m³
EMI              : 64
NSC              : 64
EMI_charge       : 0.718
NSC_charge       : -0.718
=======================================================



=====================================================================================
                        MDTransport: Data Preparation Finished
                                 2026-02-11 10:07:26
                                Elapsed Time: 0:00:00
=====================================================================================




=====================================================================================
            MDTransport: Starting Self-diffusion Coefficients Calculation
                                 2026-02-11 10:07:26
=====================================================================================

Unwrapping Trajectory: 100%|█████████████████████████| 128/128 [00:00<00:00, 9712.37species/s]
Running 3D MSD in (['x', 'y', 'z'])
Calculating MSD: 100%|█████████████████████████████████████| 2/2 [00:00<00:00, 17.74species/s]
Finding best time window: 100%|██| 3240/3240 [00:00<00:00, 205205.74window/s, avg|β-1|=0.0019]

Best time window for linear fit: 7120.0 – 15120.0 ps | avg|β-1|: 0.0019

=====================================================================================
Self-Diffusion Coefficients Summary
=====================================================================================
Species  Dimensions  Time Window (ps)  D (10⁻¹¹ m²/s)
-------------------------------------------------------
EMI      3D          7120.0-15120.0    9.555
NSC      3D          7120.0-15120.0    8.052
=====================================================================================



=====================================================================================
            MDTransport: Self-diffusion Coefficients Calculation Finished
                                 2026-02-11 10:07:26
                                Elapsed Time: 0:00:00
                               Total Wall Time: 0:00:00
=====================================================================================

asverma@Ashutoshs-iMac GROMACS %
```

2.1.5 Using `--ovito`

OVITO is required for this options and it works similar to MDAnalysis or Chemfiles. However, a `.gro` file and itp files are required to get topology details such as species charges, counts, and masses. This is similar to using `--pdb` option that doesn't need large intermediate `.pdb` files and extracts species data from `.trr` or `.xtc` files directly.

1. Navigate to `Sample/Trajectories/GROMACS` in a terminal.
2. Type `python MDTransport.py` and follow screen instruction for the post-processing as shown below or type full command e.g., For MSD calculations: `python MDTransport.py --gro --msd --ovito --tpr traj.gro --itp itp_files --t_steps 1`.

```
Last login: Wed Feb 11 10:06:53 on ttys002
asverma@Ashutoshs-iMac ~ % cd /Users/asverma/Downloads/Sample/Trajectories/GROMACS/
asverma@Ashutoshs-iMac GROMACS % python MDTransport.py
================================================================================
MDTransport: GROMACS & LAMMPS Transport Analysis Tools
Copyright (C) 2026  Ashutosh Kumar Verma, Amey Thorat and Jindal Shah

Oklahoma State University, Stillwater, OK 74078.
This is free software, and you are welcome to redistribute it
under certain conditions. See the GNU GPL for details.
================================================================================
=======================================================
          MDTransport: Validating Inputs
=======================================================

Select analysis mode:
 1. GROMACS
 2. LAMMPS

Enter number: 1

Select GROMACS input method:
 1. PDB file
 2. gmx trjconv
 3. MDAnalysis
 4. Chemfiles
 5. OVITO

Enter number: 5
Enter folder name or full path containg itp files (e.g., itp_files): itp_files
Enter name or full path of gro file (e.g., nvt.gro): nvt_prod.gro

Select one or more analysis tasks:
 1. --ONSG     Onsager Transport Coefficients Calculation
 2. --MSD      Mean Square Displacement & Self-diffusion Coefficients Calculation
 3. --NE       Nernst-Einstein Conductivity Calculation
 4. --SPDA     Spatial Decomposition Analysis of Cross-Correlations
 5. --TRA      Einstein, Self- & Cross-correlation Terms Calculation
 6. --EN       Einstein Conductivity Calculation
 7. --IPL      Ion-Pair Lifetime Calculation
 8. --CAGE     Cage Correlation Lifetime Calculation
 9. --CAGE_P   Cage Population (Cluster) Analysis
10. --RDF      Radial Distribution Function Calculation & Analysis
11. --SMD      Stefan-Maxwell Diffusivity and Conductivity Calculation
12. --VEH      Vehicular Transport Mechanism Analysis

Enter selection(s) (e.g., '1 4', '1-3,5', or '--msd --rdf').
(Enter 'all' to select all tasks)
>> 2
Enter t_steps for analysis (or press Enter for default 1) (e.g., 1): 1
t_steps = 1 ps
Enter start and end frames in ps (or press Enter to use full trajectory) (e.g., 0 2000):
Enable advanced MSD settings (MDAnalysis or Chemfiles required)? (Y/N): N
>> COM-based MSD calculations only.
Enter resnames to computes MSD, separated by spaces (e.g., EMI BF). Press Enter to continue an
d process all species:
Enter directions for MSD analysis (or press Enter for all) (e.g., x y z):
Enter resnames to skip, separated by spaces (e.g., SOL GRA). Press Enter to continue and proce
ss all species:
```

```
=======================================================
             MDTransport: Input Summary
=======================================================
Analysis Mode                                : GROMACS
GROMACS Mode                                 : OVITO
topology file                                : nvt_prod.gro
t_steps                                      : 1 ps
Analysis Task(s)                             : MSD
Number of cores                              : 8
=======================================================

Proceed with these inputs? (Y/n): Y
Continuing with execution...

=======================================================
   MDTransport: All inputs validated successfully
=======================================================


=====================================================================================
                        MDTransport: Starting Data Preparation
                                 2026-02-11 10:08:29
=====================================================================================

Trajectory does not contain data at the requested interval of 1 ps. Using Trajectory steps of
20.0 ps.
Processing Snapshots: 100%|█████████████████████████████| 1001/1001 [00:00<00:00, 1002.77it/s]


=======================================================
                 System Properties
=======================================================
Average Volume   : 26.077 nm³
Average Density  : 1594.866 kg/m³
EMI              : 64
NSC              : 64
EMI_charge       : 0.718
NSC_charge       : -0.718
=======================================================



=====================================================================================
                        MDTransport: Data Preparation Finished
                                 2026-02-11 10:08:33
                                Elapsed Time: 0:00:03
=====================================================================================



=====================================================================================
            MDTransport: Starting Self-diffusion Coefficients Calculation
                                 2026-02-11 10:08:33
=====================================================================================

Unwrapping Trajectory: 100%|█████████████████████████| 128/128 [00:00<00:00, 5179.25species/s]
Running 3D MSD in (['x', 'y', 'z'])
```

```
Calculating MSD: 100%|█████████████████████████████████████| 2/2 [00:00<00:00, 17.81species/s]
Finding best time window: 100%|██| 3240/3240 [00:00<00:00, 207148.22window/s, avg|β-1|=0.0019]

Best time window for linear fit: 7120.0 – 15120.0 ps | avg|β-1|: 0.0019

=====================================================================================
Self-Diffusion Coefficients Summary
=====================================================================================
Species  Dimensions  Time Window (ps)  D (10⁻¹¹ m²/s)
-------------------------------------------------------
EMI      3D          7120.0-15120.0    9.555
NSC      3D          7120.0-15120.0    8.052
=====================================================================================



=====================================================================================
            MDTransport: Self-diffusion Coefficients Calculation Finished
                                 2026-02-11 10:08:33
                                Elapsed Time: 0:00:00
                               Total Wall Time: 0:00:03
=====================================================================================

asverma@Ashutoshs-iMac GROMACS %
```

2.2 Post-processing LAMMPS Files

Note that LAMMPS accepts numerical input only, so species names and their counts must be specified in the same order (e.g., `--species EMI 64 NSC 64`) as they appear in the data file.

2.2.1 Using `--traj`

It requires a `.lammpstrj` and data files as shown in Figure 1.

1. Navigate to `Sample/Trajectories/LAMMPS` in a terminal.
2. Type `python MDTransport.py` and follow screen instruction for the post-processing as shown below or type full command e.g., For MSD calculations: `python MDTransport.py --lam --msd --traj traj.lammpstrj --data data.lmp --t_steps 1 --species EMI 64 NSC 64 --dump_frame 20000`.

```
Last login: Wed Feb 11 10:09:15 on ttys002
asverma@Ashutoshs-iMac ~ % cd /Users/asverma/Downloads/Sample/Trajectories/LAMMPS
asverma@Ashutoshs-iMac LAMMPS % python MDTransport.py
================================================================================
MDTransport: GROMACS & LAMMPS Transport Analysis Tools
Copyright (C) 2026  Ashutosh Kumar Verma, Amey Thorat and Jindal Shah

Oklahoma State University, Stillwater, OK 74078.
This is free software, and you are welcome to redistribute it
under certain conditions. See the GNU GPL for details.
================================================================================
=======================================================
          MDTransport: Validating Inputs
=======================================================

Select analysis mode:
 1. GROMACS
 2. LAMMPS

Enter number: 2

Select LAMMPS input method:
 1. lammpstrj
 2. com file
 3. MDAnalysis
 4. Chemfiles
 5. OVITO

Enter number: 1
Enter LAMMPS *.lammpstrj file name or full path (e.g., traj.lammpstrj): traj.lammpstrj

Select one or more analysis tasks:
 1. --ONSG     Onsager Transport Coefficients Calculation
 2. --MSD      Mean Square Displacement & Self-diffusion Coefficients Calculation
 3. --NE       Nernst-Einstein Conductivity Calculation
 4. --SPDA     Spatial Decomposition Analysis of Cross-Correlations
 5. --TRA      Einstein, Self- & Cross-correlation Terms Calculation
 6. --EN       Einstein Conductivity Calculation
 7. --IPL      Ion-Pair Lifetime Calculation
 8. --CAGE     Cage Correlation Lifetime Calculation
 9. --CAGE_P   Cage Population (Cluster) Analysis
10. --RDF      Radial Distribution Function Calculation & Analysis
11. --SMD      Stefan-Maxwell Diffusivity and Conductivity Calculation
12. --VEH      Vehicular Transport Mechanism Analysis

Enter selection(s) (e.g., '1 4', '1-3,5', or '--msd --rdf').
(Enter 'all' to select all tasks)
>> 2
Topology missing. Need LAMMPS data file.
Enter path to LAMMPS data file:
data.lmp

=====================================================================================
SYSTEM TOPOLOGY (LAMMPS)
=====================================================================================
Type      | Count     | Mass          | Atom     | Selection
-------------------------------------------------------------------------------------
1         | 64        | 12.011        | C        | type 1
2         | 128       | 12.011        | C        | type 2
```

```
3        | 64       | 1.008        | H      | type 3
4        | 128      | 1.008        | H      | type 4
5        | 64       | 12.011       | C      | type 5
6        | 192      | 1.008        | H      | type 6
7        | 128      | 14.007       | N      | type 7
8        | 64       | 12.011       | C      | type 8
9        | 128      | 1.008        | H      | type 9
10       | 64       | 12.011       | C      | type 10
11       | 192      | 1.008        | H      | type 11
12       | 64       | 14.000       | N      | type 12
13       | 128      | 32.066       | S      | type 13
14       | 256      | 15.999       | O      | type 14
15       | 128      | 12.011       | C      | type 15
16       | 384      | 18.998       | F      | type 16
==========================================================================

Enter the frame stride (process every N-th frame, press Enter for default 1): 1
N = 1
Enter t_steps for analysis (or press Enter for default 1) (e.g., 1): 1
t_steps = 1 ps
Enter species and counts (e.g., EMI 500 BF 500): EMI 64 NSC 64
Enter dump_frame of LAMMPS trajectory (or press Enter for default 1000) (e.g., 2000): 20000
dump_frame = 20000 steps
Enter start and end frames in ps (or press Enter to use full trajectory) (e.g., 0 2000):
Enable advanced MSD settings (MDAnalysis or Chemfiles required)? (Y/N): N
>> COM-based MSD calculations only.
Enter resnames to computes MSD, separated by spaces (e.g., EMI BF). Press Enter to continue an
d process all species:
Enter directions for MSD analysis (or press Enter for all) (e.g., x y z):


=======================================================
             MDTransport: Input Summary
=======================================================
Analysis Mode                            : LAMMPS
LAMMPS Mode                              : lammpstrj file
lammpstrj file                           : traj.lammpstrj
DATA File                                : data.lmp
Species/Counts                           : EMI 64 NSC 64
t_steps                                  : 1 ps
N                                        : 1
Analysis Task(s)                         : MSD
Number of cores                          : 8
=======================================================

Proceed with these inputs? (Y/n): Y
Continuing with execution...

=======================================================
   MDTransport: All inputs validated successfully
=======================================================


==========================================================================
                     MDTransport: Starting Data Preparation
                              2026-02-11 10:11:24
==========================================================================

Processing Snapshots: 1001it [00:10, 98.56it/s]
```

```
=======================================================
                 System Properties
=======================================================
Average Volume   : 26.077 nm³
Average Density  : 1594.762 kg/m³
EMI              : 64
NSC              : 64
EMI_charges      : 0.718
NSC_charges      : -0.718
=======================================================



=====================================================================================
                        MDTransport: Data Preparation Finished
                                 2026-02-11 10:11:34
                                Elapsed Time: 0:00:10
=====================================================================================



=====================================================================================
            MDTransport: Starting Self-diffusion Coefficients Calculation
                                 2026-02-11 10:11:34
=====================================================================================

Unwrapping Trajectory: 100%|████████████████████████████| 128/128 [00:00<00:00, 25991.04mol/s]
Creating species DataFrames: 100%|████████████████████████| 128/128 [00:00<00:00, 7717.77it/s]
Running 3D MSD in (['x', 'y', 'z'])
Calculating MSD: 100%|█████████████████████████████████████| 2/2 [00:00<00:00, 17.34species/s]
Finding best time window: 100%|██| 3240/3240 [00:00<00:00, 298704.14window/s, avg|β-1|=0.0469]

Best time window for linear fit: 2020.0 – 10020.0 ps | avg|β-1|: 0.0469

=====================================================================================
Self-Diffusion Coefficients Summary
=====================================================================================
Species  Dimensions  Time Window (ps)  D (10⁻¹¹ m²/s)
-------------------------------------------------------
EMI      3D          2020.0-10020.0    4.130
NSC      3D          2020.0-10020.0    2.712
=====================================================================================



=====================================================================================
            MDTransport: Self-diffusion Coefficients Calculation Finished
                                 2026-02-11 10:11:34
                                Elapsed Time: 0:00:00
                               Total Wall Time: 0:00:10
=====================================================================================

asverma@Ashutoshs-iMac LAMMPS %
```

2.2.2 Using `--com`

It requires a `com.txt` generated from LAMMPS simulations and data files as shown in Figure 1.

1. Navigate to `Sample/Trajectories/LAMMPS` in a terminal.
2. Type `python MDTransport.py` and follow screen instruction for the post-processing as shown below or type full command e.g., For MSD calculations: `python MDTransport.py --lam --msd --com com.txt --data data.lmp --t_steps 1 --species EMI 64 NSC 64 --dump_frame 20000`.

One can add below three line of commands to LAMMPS input files to generate com.txt for saving disk storage.

1. compute molchunk all chunk/atom molecule
2. compute comchunk all com/chunk molchunk
3. fix com_output all ave/time 5000 1 5000 c_comchunk[*] file com.txt mode vector

```
Last login: Wed Feb 11 10:12:01 on ttys002
asverma@Ashutoshs-iMac ~ % cd /Users/asverma/Downloads/Sample/Trajectories/LAMMPS
asverma@Ashutoshs-iMac LAMMPS % python MDTransport.py
================================================================================
MDTransport: GROMACS & LAMMPS Transport Analysis Tools
Copyright (C) 2026  Ashutosh Kumar Verma, Amey Thorat and Jindal Shah

Oklahoma State University, Stillwater, OK 74078.
This is free software, and you are welcome to redistribute it
under certain conditions. See the GNU GPL for details.
================================================================================
=======================================================
          MDTransport: Validating Inputs
=======================================================

Select analysis mode:
 1. GROMACS
 2. LAMMPS

Enter number: 2

Select LAMMPS input method:
 1. lammpstrj
 2. com file
 3. MDAnalysis
 4. Chemfiles
 5. OVITO

Enter number: 2
Enter LAMMPS com file name or full path (e.g., com.lmp): com.txt
Enter LAMMPS data file name or full path (e.g., lammps.data): data.lmp

Select one or more analysis tasks:
 1. --ONSG     Onsager Transport Coefficients Calculation
 2. --MSD      Mean Square Displacement & Self-diffusion Coefficients Calculation
 3. --NE       Nernst-Einstein Conductivity Calculation
 4. --SPDA     Spatial Decomposition Analysis of Cross-Correlations
 5. --TRA      Einstein, Self- & Cross-correlation Terms Calculation
 6. --EN       Einstein Conductivity Calculation
 7. --IPL      Ion-Pair Lifetime Calculation
 8. --CAGE     Cage Correlation Lifetime Calculation
 9. --CAGE_P   Cage Population (Cluster) Analysis
10. --RDF      Radial Distribution Function Calculation & Analysis
11. --SMD      Stefan-Maxwell Diffusivity and Conductivity Calculation
12. --VEH      Vehicular Transport Mechanism Analysis

Enter selection(s) (e.g., '1 4', '1-3,5', or '--msd --rdf').
(Enter 'all' to select all tasks)
>> 2

=====================================================================================
SYSTEM TOPOLOGY (LAMMPS)
=====================================================================================
Type     | Count    | Mass         | Atom   | Selection
-------------------------------------------------------------------------------------
1        | 64       | 12.011       | C      | type 1
2        | 128      | 12.011       | C      | type 2
3        | 64       | 1.008        | H      | type 3
4        | 128      | 1.008        | H      | type 4
```

```
5         | 64        | 12.011        | C      | type 5
6         | 192       | 1.008         | H      | type 6
7         | 128       | 14.007        | N      | type 7
8         | 64        | 12.011        | C      | type 8
9         | 128       | 1.008         | H      | type 9
10        | 64        | 12.011        | C      | type 10
11        | 192       | 1.008         | H      | type 11
12        | 64        | 14.000        | N      | type 12
13        | 128       | 32.066        | S      | type 13
14        | 256       | 15.999        | O      | type 14
15        | 128       | 12.011        | C      | type 15
16        | 384       | 18.998        | F      | type 16
=====================================================================================

Enter the frame stride (process every N-th frame, press Enter for default 1): 1
N = 1
Enter t_steps for analysis (or press Enter for default 1) (e.g., 1): 1
t_steps = 1 ps
Enter species and counts (e.g., EMI 500 BF 500): EMI 64 NSC 64
Enter start and end frames in ps (or press Enter to use full trajectory) (e.g., 0 2000):
Enable advanced MSD settings (MDAnalysis or Chemfiles required)? (Y/N): N
>> COM-based MSD calculations only.
Enter resnames to computes MSD, separated by spaces (e.g., EMI BF). Press Enter to continue and
 process all species:
Enter directions for MSD analysis (or press Enter for all) (e.g., x y z):


=======================================================
             MDTransport: Input Summary
=======================================================
Analysis Mode                              : LAMMPS
COM File                                   : com.txt
DATA File                                  : data.lmp
Species/Counts                             : EMI 64 NSC 64
t_steps                                    : 1 ps
N                                          : 1
Analysis Task(s)                           : MSD
Number of cores                            : 8
=======================================================

Proceed with these inputs? (Y/n): Y
Continuing with execution...

=======================================================
   MDTransport: All inputs validated successfully
=======================================================


=====================================================================================
                         MDTransport: Starting Data Preparation
                                  2026-02-11 10:13:30
=====================================================================================

Reading COM file: 1001it [00:00, 23474.97it/s]


=======================================================
                 System Properties
=======================================================
```

```
Average Volume   : 26.077 nm³
EMI              : 64
NSC              : 64
EMI_charges      : 0.718
NSC_charges      : -0.718
=======================================================



=====================================================================================
                       MDTransport: Data Preparation Finished
                                2026-02-11 10:13:30
                               Elapsed Time: 0:00:00
=====================================================================================



=====================================================================================
            MDTransport: Starting Self-diffusion Coefficients Calculation
                                2026-02-11 10:13:30
=====================================================================================

Unwrapping Trajectory: 100%|█████████████████████████████| 128/128 [00:00<00:00, 27825.80mol/s]
Creating species DataFrames: 100%|█████████████████████████| 128/128 [00:00<00:00, 7443.51it/s]
Running 3D MSD in (['x', 'y', 'z'])
Calculating MSD: 100%|██████████████████████████████████████| 2/2 [00:00<00:00, 19.81species/s]
Finding best time window: 100%|███| 3240/3240 [00:00<00:00, 307664.59window/s, avg|β-1|=0.0469]

Best time window for linear fit: 2020.0 – 10020.0 ps | avg|β-1|: 0.0469

=====================================================================================
Self-Diffusion Coefficients Summary
=====================================================================================
Species  Dimensions  Time Window (ps)  D (10⁻¹¹ m²/s)
-------------------------------------------------------
EMI      3D          2020.0-10020.0    4.130
NSC      3D          2020.0-10020.0    2.712
=====================================================================================



=====================================================================================
            MDTransport: Self-diffusion Coefficients Calculation Finished
                                2026-02-11 10:13:30
                               Elapsed Time: 0:00:00
                              Total Wall Time: 0:00:00
=====================================================================================

asverma@Ashutoshs-iMac LAMMPS %
```

2.2.3 Using `--mda`

It requires a LAMMPS trajectory files in any formats such as `.lammpstrj`, `.xtc`, `.xyz`, etc. and data files as shown in Figure 1.

1. Navigate to `Sample/Trajectories/LAMMPS` in a terminal.
2. Type `python MDTransport.py` and follow screen instruction for the post-processing as shown below or type full command e.g., For MSD calculations: `python MDTransport.py --lam --msd --mda --traj traj.xtc --data data.lmp --t_steps 1 --species EMI 64 NSC 64 --dump_frame 20000`.

```
Last login: Wed Feb 11 10:12:53 on ttys002
asverma@Ashutoshs-iMac ~ % cd /Users/asverma/Downloads/Sample/Trajectories/LAMMPS
asverma@Ashutoshs-iMac LAMMPS % python MDTransport.py
================================================================================
MDTransport: GROMACS & LAMMPS Transport Analysis Tools
Copyright (C) 2026  Ashutosh Kumar Verma, Amey Thorat and Jindal Shah

Oklahoma State University, Stillwater, OK 74078.
This is free software, and you are welcome to redistribute it
under certain conditions. See the GNU GPL for details.
================================================================================
=======================================================
          MDTransport: Validating Inputs
=======================================================

Select analysis mode:
 1. GROMACS
 2. LAMMPS

Enter number: 2

Select LAMMPS input method:
 1. lammpstrj
 2. com file
 3. MDAnalysis
 4. Chemfiles
 5. OVITO

Enter number: 3
Enter LAMMPS trajectory file name or full path (e.g., traj.lammpstrj or traj.xtc): traj.xtc
Enter LAMMPS data file name or full path (e.g., lammps.data): data.lmp

Select one or more analysis tasks:
 1. --ONSG     Onsager Transport Coefficients Calculation
 2. --MSD      Mean Square Displacement & Self-diffusion Coefficients Calculation
 3. --NE       Nernst-Einstein Conductivity Calculation
 4. --SPDA     Spatial Decomposition Analysis of Cross-Correlations
 5. --TRA      Einstein, Self- & Cross-correlation Terms Calculation
 6. --EN       Einstein Conductivity Calculation
 7. --IPL      Ion-Pair Lifetime Calculation
 8. --CAGE     Cage Correlation Lifetime Calculation
 9. --CAGE_P   Cage Population (Cluster) Analysis
10. --RDF      Radial Distribution Function Calculation & Analysis
11. --SMD      Stefan-Maxwell Diffusivity and Conductivity Calculation
12. --VEH      Vehicular Transport Mechanism Analysis

Enter selection(s) (e.g., '1 4', '1-3,5', or '--msd --rdf').
(Enter 'all' to select all tasks)
>> 2

=====================================================================================
SYSTEM TOPOLOGY (LAMMPS)
=====================================================================================
Type     | Count     | Mass          | Atom    | Selection
-------------------------------------------------------------------------------------
1        | 64        | 12.011        | C       | type 1
2        | 128       | 12.011        | C       | type 2
3        | 64        | 1.008         | H       | type 3
4        | 128       | 1.008         | H       | type 4
```

```
5         | 64        | 12.011        | C        | type 5
6         | 192       | 1.008         | H        | type 6
7         | 128       | 14.007        | N        | type 7
8         | 64        | 12.011        | C        | type 8
9         | 128       | 1.008         | H        | type 9
10        | 64        | 12.011        | C        | type 10
11        | 192       | 1.008         | H        | type 11
12        | 64        | 14.000        | N        | type 12
13        | 128       | 32.066        | S        | type 13
14        | 256       | 15.999        | O        | type 14
15        | 128       | 12.011        | C        | type 15
16        | 384       | 18.998        | F        | type 16
======================================================================================

Enter the frame stride (process every N-th frame, press Enter for default 1): 1
N = 1
Enter t_steps for analysis (or press Enter for default 1) (e.g., 1): 1
t_steps = 1 ps
Enter species and counts (e.g., EMI 500 BF 500): EMI 64 NSC 64
Enter dump_frame of LAMMPS trajectory (or press Enter for default 1000) (e.g., 2000): 20000
dump_frame = 20000 steps
Enter start and end frames in ps (or press Enter to use full trajectory) (e.g., 0 2000):
Enable advanced MSD settings (MDAnalysis or Chemfiles required)? (Y/N): N
>> COM-based MSD calculations only.
Enter resnames to computes MSD, separated by spaces (e.g., EMI BF). Press Enter to continue an
d process all species:
Enter directions for MSD analysis (or press Enter for all) (e.g., x y z):


=======================================================
             MDTransport: Input Summary
=======================================================
Analysis Mode                             : LAMMPS
LAMMPS Mode                               : MDAnalysis
lammpstrj file                            : traj.xtc
DATA File                                 : data.lmp
dump_frame                                : 20000 steps
Species/Counts                            : EMI 64 NSC 64
t_steps                                   : 1 ps
N                                         : 1
Analysis Task(s)                          : MSD
Number of cores                           : 8
=======================================================

Proceed with these inputs? (Y/n): Y
Continuing with execution...

=======================================================
   MDTransport: All inputs validated successfully
=======================================================


======================================================================================
                        MDTransport: Starting Data Preparation
                                 2026-02-11 10:14:35
======================================================================================

Processing Snapshots: 100%|██████████████████████████████| 1001/1001 [00:01<00:00, 849.26it/s]
```

```
=======================================================
                 System Properties
=======================================================
Average Volume   : 26.077 nm³
Average Density  : 1594.762 kg/m³
EMI              : 64
NSC              : 64
EMI_charges      : 0.718
NSC_charges      : -0.718
=======================================================



=====================================================================================
                        MDTransport: Data Preparation Finished
                                 2026-02-11 10:14:37
                                Elapsed Time: 0:00:01
=====================================================================================



=====================================================================================
            MDTransport: Starting Self-diffusion Coefficients Calculation
                                 2026-02-11 10:14:37
=====================================================================================

Unwrapping Trajectory: 100%|████████████████████████████| 128/128 [00:00<00:00, 28071.68mol/s]
Creating species DataFrames: 100%|████████████████████████| 128/128 [00:00<00:00, 4922.71it/s]
Running 3D MSD in (['x', 'y', 'z'])
Calculating MSD: 100%|█████████████████████████████████████| 2/2 [00:00<00:00, 18.65species/s]
Finding best time window: 100%|██| 3240/3240 [00:00<00:00, 298310.72window/s, avg|β-1|=0.0469]

Best time window for linear fit: 2020.0 – 10020.0 ps | avg|β-1|: 0.0469

=====================================================================================
Self-Diffusion Coefficients Summary
=====================================================================================
Species  Dimensions  Time Window (ps)  D (10⁻¹¹ m²/s)
-------------------------------------------------------
EMI      3D          2020.0-10020.0    4.130
NSC      3D          2020.0-10020.0    2.712
=====================================================================================



=====================================================================================
            MDTransport: Self-diffusion Coefficients Calculation Finished
                                 2026-02-11 10:14:37
                                Elapsed Time: 0:00:00
                               Total Wall Time: 0:00:01
=====================================================================================

asverma@Ashutoshs-iMac LAMMPS %
```

2.2.4 Using `--chem`

Similar to MDAnalysis, it requires a LAMMPS trajectory files in any formats such as `.lammpstrj`, `.xtc`, `.xyz`, etc. and data files as shown in Figure 1.

1. Navigate to `Sample/Trajectories/LAMMPS` in a terminal.
2. Type `python MDTransport.py` and follow screen instruction for the post-processing as shown below or type full command e.g., For MSD calculations: `python MDTransport.py --lam --msd --chem --traj traj.xtc --data data.lmp --t_steps 1 --species EMI 64 NSC 64 --dump_frame 20000`.

```
Last login: Wed Feb 11 10:13:49 on ttys002
asverma@Ashutoshs-iMac ~ % cd /Users/asverma/Downloads/Sample/Trajectories/LAMMPS
asverma@Ashutoshs-iMac LAMMPS % python MDTransport.py
================================================================================
MDTransport: GROMACS & LAMMPS Transport Analysis Tools
Copyright (C) 2026  Ashutosh Kumar Verma, Amey Thorat and Jindal Shah

Oklahoma State University, Stillwater, OK 74078.
This is free software, and you are welcome to redistribute it
under certain conditions. See the GNU GPL for details.
================================================================================
=======================================================
          MDTransport: Validating Inputs
=======================================================

Select analysis mode:
 1. GROMACS
 2. LAMMPS

Enter number: 2

Select LAMMPS input method:
 1. lammpstrj
 2. com file
 3. MDAnalysis
 4. Chemfiles
 5. OVITO

Enter number: 4
Enter LAMMPS trajectory file name or full path (e.g., traj.lammpstrj or traj.xtc): traj.xtc
Enter LAMMPS data file name or full path (e.g., lammps.data): data.lmp

Select one or more analysis tasks:
 1. --ONSG     Onsager Transport Coefficients Calculation
 2. --MSD      Mean Square Displacement & Self-diffusion Coefficients Calculation
 3. --NE       Nernst-Einstein Conductivity Calculation
 4. --SPDA     Spatial Decomposition Analysis of Cross-Correlations
 5. --TRA      Einstein, Self- & Cross-correlation Terms Calculation
 6. --EN       Einstein Conductivity Calculation
 7. --IPL      Ion-Pair Lifetime Calculation
 8. --CAGE     Cage Correlation Lifetime Calculation
 9. --CAGE_P   Cage Population (Cluster) Analysis
10. --RDF      Radial Distribution Function Calculation & Analysis
11. --SMD      Stefan-Maxwell Diffusivity and Conductivity Calculation
12. --VEH      Vehicular Transport Mechanism Analysis

Enter selection(s) (e.g., '1 4', '1-3,5', or '--msd --rdf').
(Enter 'all' to select all tasks)
>> 2

=====================================================================================
SYSTEM TOPOLOGY (LAMMPS)
=====================================================================================
Type     | Count    | Mass         | Atom   | Selection
-------------------------------------------------------------------------------------
1        | 64       | 12.011       | C      | type 1
2        | 128      | 12.011       | C      | type 2
3        | 64       | 1.008        | H      | type 3
4        | 128      | 1.008        | H      | type 4
```

```
5         | 64        | 12.011      | C       | type 5
6         | 192       | 1.008       | H       | type 6
7         | 128       | 14.007      | N       | type 7
8         | 64        | 12.011      | C       | type 8
9         | 128       | 1.008       | H       | type 9
10        | 64        | 12.011      | C       | type 10
11        | 192       | 1.008       | H       | type 11
12        | 64        | 14.000      | N       | type 12
13        | 128       | 32.066      | S       | type 13
14        | 256       | 15.999      | O       | type 14
15        | 128       | 12.011      | C       | type 15
16        | 384       | 18.998      | F       | type 16
=====================================================================================

Enter the frame stride (process every N-th frame, press Enter for default 1): 1
N = 1
Enter t_steps for analysis (or press Enter for default 1) (e.g., 1): 1
t_steps = 1 ps
Enter species and counts (e.g., EMI 500 BF 500): EMI 64 NSC 64
Enter dump_frame of LAMMPS trajectory (or press Enter for default 1000) (e.g., 2000): 20000
dump_frame = 20000 steps
Enter start and end frames in ps (or press Enter to use full trajectory) (e.g., 0 2000):
Enable advanced MSD settings (MDAnalysis or Chemfiles required)? (Y/N): N
>> COM-based MSD calculations only.
Enter resnames to computes MSD, separated by spaces (e.g., EMI BF). Press Enter to continue an
d process all species:
Enter directions for MSD analysis (or press Enter for all) (e.g., x y z):


=======================================================
             MDTransport: Input Summary
=======================================================
Analysis Mode                              : LAMMPS
LAMMPS Mode                                : Chemfiles
lammpstrj file                             : traj.xtc
DATA File                                  : data.lmp
dump_frame                                 : 20000 steps
Species/Counts                             : EMI 64 NSC 64
t_steps                                    : 1 ps
N                                          : 1
Analysis Task(s)                           : MSD
Number of cores                            : 8
=======================================================

Proceed with these inputs? (Y/n): Y
Continuing with execution...

=======================================================
   MDTransport: All inputs validated successfully
=======================================================


=====================================================================================
                        MDTransport: Starting Data Preparation
                                 2026-02-11 10:15:50
=====================================================================================

Processing Snapshots: 100%|██████████████████████████████| 1001/1001 [00:00<00:00, 1161.26it/s]
```

```
=======================================================
                 System Properties
=======================================================
Average Volume   : 26.077 nm³
Average Density  : 1594.762 kg/m³
EMI              : 64
NSC              : 64
EMI_charges      : 0.718
NSC_charges      : -0.718
=======================================================



=====================================================================================
                        MDTransport: Data Preparation Finished
                                 2026-02-11 10:15:51
                                Elapsed Time: 0:00:01
=====================================================================================



=====================================================================================
            MDTransport: Starting Self-diffusion Coefficients Calculation
                                 2026-02-11 10:15:51
=====================================================================================

Unwrapping Trajectory: 100%|████████████████████████████| 128/128 [00:00<00:00, 20301.41mol/s]
Creating species DataFrames: 100%|████████████████████████| 128/128 [00:00<00:00, 6951.23it/s]
Running 3D MSD in (['x', 'y', 'z'])
Calculating MSD: 100%|█████████████████████████████████████| 2/2 [00:00<00:00, 15.43species/s]
Finding best time window: 100%|██| 3240/3240 [00:00<00:00, 292204.29window/s, avg|β-1|=0.0469]

Best time window for linear fit: 2020.0 – 10020.0 ps | avg|β-1|: 0.0469

=====================================================================================
Self-Diffusion Coefficients Summary
=====================================================================================
Species  Dimensions  Time Window (ps)  D (10⁻¹¹ m²/s)
-------------------------------------------------------
EMI      3D          2020.0-10020.0    4.130
NSC      3D          2020.0-10020.0    2.712
=====================================================================================



=====================================================================================
            MDTransport: Self-diffusion Coefficients Calculation Finished
                                 2026-02-11 10:15:51
                                Elapsed Time: 0:00:00
                               Total Wall Time: 0:00:01
=====================================================================================

asverma@Ashutoshs-iMac LAMMPS %
```

2.2.5 Using `--ovito`

Similar to MDAnalysis and Chemfiles, it requires a LAMMPS trajectory files in any formats such as `.lammpstrj`, `.xtc`, `.xyz`, etc. and data files as shown in Figure 1.

1. Navigate to `Sample/Trajectories/LAMMPS` in a terminal.
2. Type `python MDTransport.py` and follow screen instruction for the post-processing as shown below or type full command e.g., For MSD calculations: `python MDTransport.py --lam --msd --ovito --traj traj.xtc --data data.lmp --t_steps 1 --species EMI 64 NSC 64 --dump_frame 20000`.

```
Last login: Wed Feb 11 10:14:56 on ttys002
asverma@Ashutoshs-iMac ~ % cd /Users/asverma/Downloads/Sample/Trajectories/LAMMPS
asverma@Ashutoshs-iMac LAMMPS % python MDTransport.py
================================================================================
MDTransport: GROMACS & LAMMPS Transport Analysis Tools
Copyright (C) 2026  Ashutosh Kumar Verma, Amey Thorat and Jindal Shah

Oklahoma State University, Stillwater, OK 74078.
This is free software, and you are welcome to redistribute it
under certain conditions. See the GNU GPL for details.
================================================================================
=======================================================
          MDTransport: Validating Inputs
=======================================================

Select analysis mode:
 1. GROMACS
 2. LAMMPS

Enter number: 2

Select LAMMPS input method:
 1. lammpstrj
 2. com file
 3. MDAnalysis
 4. Chemfiles
 5. OVITO

Enter number: 5
Enter LAMMPS trajectory file name or full path (e.g., traj.lammpstrj or traj.xtc): traj.xtc
Enter LAMMPS data file name or full path (e.g., lammps.data): data.lmp

Select one or more analysis tasks:
 1. --ONSG     Onsager Transport Coefficients Calculation
 2. --MSD      Mean Square Displacement & Self-diffusion Coefficients Calculation
 3. --NE       Nernst-Einstein Conductivity Calculation
 4. --SPDA     Spatial Decomposition Analysis of Cross-Correlations
 5. --TRA      Einstein, Self- & Cross-correlation Terms Calculation
 6. --EN       Einstein Conductivity Calculation
 7. --IPL      Ion-Pair Lifetime Calculation
 8. --CAGE     Cage Correlation Lifetime Calculation
 9. --CAGE_P   Cage Population (Cluster) Analysis
10. --RDF      Radial Distribution Function Calculation & Analysis
11. --SMD      Stefan-Maxwell Diffusivity and Conductivity Calculation
12. --VEH      Vehicular Transport Mechanism Analysis

Enter selection(s) (e.g., '1 4', '1-3,5', or '--msd --rdf').
(Enter 'all' to select all tasks)
>> 2

=====================================================================================
SYSTEM TOPOLOGY (LAMMPS)
=====================================================================================
Type     | Count    | Mass         | Atom   | Selection
-------------------------------------------------------------------------------------
1        | 64       | 12.011       | C      | type 1
2        | 128      | 12.011       | C      | type 2
3        | 64       | 1.008        | H      | type 3
4        | 128      | 1.008        | H      | type 4
```

```
5        | 64      | 12.011      | C      | type 5
6        | 192     | 1.008       | H      | type 6
7        | 128     | 14.007      | N      | type 7
8        | 64      | 12.011      | C      | type 8
9        | 128     | 1.008       | H      | type 9
10       | 64      | 12.011      | C      | type 10
11       | 192     | 1.008       | H      | type 11
12       | 64      | 14.000      | N      | type 12
13       | 128     | 32.066      | S      | type 13
14       | 256     | 15.999      | O      | type 14
15       | 128     | 12.011      | C      | type 15
16       | 384     | 18.998      | F      | type 16
=====================================================================================

Enter the frame stride (process every N-th frame, press Enter for default 1): 1
N = 1
Enter t_steps for analysis (or press Enter for default 1) (e.g., 1): 1
t_steps = 1 ps
Enter species and counts (e.g., EMI 500 BF 500): EMI 64 NSC 64
Enter dump_frame of LAMMPS trajectory (or press Enter for default 1000) (e.g., 2000): 20000
dump_frame = 20000 steps
Enter start and end frames in ps (or press Enter to use full trajectory) (e.g., 0 2000):
Enable advanced MSD settings (MDAnalysis or Chemfiles required)? (Y/N): N
>> COM-based MSD calculations only.
Enter resnames to computes MSD, separated by spaces (e.g., EMI BF). Press Enter to continue and
 process all species:
Enter directions for MSD analysis (or press Enter for all) (e.g., x y z):


=======================================================
             MDTransport: Input Summary
=======================================================
Analysis Mode                            : LAMMPS
LAMMPS Mode                              : OVITO
lammpstrj file                           : traj.xtc
DATA File                                : data.lmp
dump_frame                               : 20000 steps
Species/Counts                           : EMI 64 NSC 64
t_steps                                  : 1 ps
N                                        : 1
Analysis Task(s)                         : MSD
Number of cores                          : 8
=======================================================

Proceed with these inputs? (Y/n): Y
Continuing with execution...

=======================================================
   MDTransport: All inputs validated successfully
=======================================================


=====================================================================================
                        MDTransport: Starting Data Preparation
                                 2026-02-11 10:16:58
=====================================================================================

No atom IDs found in trajectory (likely XTC/TRR). Assuming atoms are sorted (1..N).
Processing Snapshots: 100%|██████████████████████████████| 1001/1001 [00:00<00:00, 1434.45it/s]
```

```
=======================================================
                 System Properties
=======================================================
Average Volume   : 26.077 nm³
Average Density  : 1594.762 kg/m³
EMI              : 64
NSC              : 64
EMI_charges      : 0.718
NSC_charges      : -0.718
=======================================================



=====================================================================================
                        MDTransport: Data Preparation Finished
                                 2026-02-11 10:17:01
                                Elapsed Time: 0:00:03
=====================================================================================



=====================================================================================
            MDTransport: Starting Self-diffusion Coefficients Calculation
                                 2026-02-11 10:17:01
=====================================================================================

Unwrapping Trajectory: 100%|█████████████████████████████| 128/128 [00:00<00:00, 15419.79mol/s]
Creating species DataFrames: 100%|███████████████████████| 128/128 [00:00<00:00, 4268.94it/s]
Running 3D MSD in (['x', 'y', 'z'])
Calculating MSD: 100%|██████████████████████████████████████| 2/2 [00:00<00:00, 11.52species/s]
Finding best time window: 100%|███| 3240/3240 [00:00<00:00, 283564.50window/s, avg|β-1|=0.0469]

Best time window for linear fit: 2020.0 – 10020.0 ps | avg|β-1|: 0.0469

=====================================================================================
Self-Diffusion Coefficients Summary
=====================================================================================
Species  Dimensions  Time Window (ps)  D (10⁻¹¹ m²/s)
-------------------------------------------------------
EMI      3D          2020.0-10020.0    4.130
NSC      3D          2020.0-10020.0    2.712
=====================================================================================



=====================================================================================
            MDTransport: Self-diffusion Coefficients Calculation Finished
                                 2026-02-11 10:17:02
                                Elapsed Time: 0:00:00
                               Total Wall Time: 0:00:03
=====================================================================================

asverma@Ashutoshs-iMac LAMMPS %
```
